# The PfRCR complex bridges malaria parasite and erythrocyte during invasion

**DOI:** 10.1038/s41586-023-06856-1

**Published:** 2023-12-20

**Authors:** Brendan Farrell, Nawsad Alam, Melissa N. Hart, Abhishek Jamwal, Robert J. Ragotte, Hannah Walters-Morgan, Simon J. Draper, Ellen Knuepfer, Matthew K. Higgins

**Affiliations:** 1 Department of Biochemistry, University of Oxford, Oxford, UK; 2 Kavli Institute for Nanoscience Discovery, University of Oxford, Oxford, UK; 3 The Royal Veterinary College, Hatfield, UK

**Keywords:** Parasite biology, Cryoelectron microscopy

## Abstract

The symptoms of malaria occur during the blood stage of infection, when parasites invade and replicate within human erythrocytes. The PfPCRCR complex^1^, containing PfRH5 (refs. ^2,3^), PfCyRPA, PfRIPR, PfCSS and PfPTRAMP, is essential for erythrocyte invasion by the deadliest human malaria parasite, *Plasmodium falciparum*. Invasion can be prevented by antibodies^3–6^ or nanobodies^1^ against each of these conserved proteins, making them the leading blood-stage malaria vaccine candidates. However, little is known about how PfPCRCR functions during invasion. Here we present the structure of the PfRCR complex^7,8^, containing PfRH5, PfCyRPA and PfRIPR, determined by cryogenic-electron microscopy. We test the hypothesis that PfRH5 opens to insert into the membrane^9^, instead showing that a rigid, disulfide-locked PfRH5 can mediate efficient erythrocyte invasion. We show, through modelling and an erythrocyte-binding assay, that PfCyRPA-binding antibodies^5^ neutralize invasion through a steric mechanism. We determine the structure of PfRIPR, showing that it consists of an ordered, multidomain core flexibly linked to an elongated tail. We also show that the elongated tail of PfRIPR, which is the target of growth-neutralizing antibodies^6^, binds to the PfCSS–PfPTRAMP complex on the parasite membrane. A modular PfRIPR is therefore linked to the merozoite membrane through an elongated tail, and its structured core presents PfCyRPA and PfRH5 to interact with erythrocyte receptors. This provides fresh insight into the molecular mechanism of erythrocyte invasion and opens the way to new approaches in rational vaccine design.

## Abstract (web summary)

Structural studies show how the PfRCR complex of *Plasmodium falciparum* forms a bridge between erythrocyte and parasite membranes, and how PfCyRPA-binding antibodies neutralize invasion through a steric mechanism, opening the way to new approaches in rational vaccine design.

## Main

Erythrocyte invasion by *Plasmodium falciparum* involves a tightly ordered sequence of events, starting when the merozoite form of the parasite contacts an erythrocyte^10,11^. This is followed by both a strong, actin-dependent deformation of the erythrocyte surface and reorientation of the merozoite to place its apical pole adjacent to the erythrocyte membrane. Discharge of apical organelles releases the machinery required for invasion, including the PfRCR complex. This leads to both a calcium spike at the merozoite–erythrocyte contact site and the formation of a moving junction between the merozoite and erythrocyte. The parasite then actively pulls its way inside the erythrocyte, followed immediately by a series of deformations of the infected erythrocyte and the establishment of the parasite inside a vacuole.

These stages of erythrocyte invasion require multiple host–parasite interactions, many of which are mediated by protein families with redundant functions^10^. However, no strain of *P. falciparum* has yet been found that can invade erythrocytes when the interaction between PfRH5 (refs. ^12,13^) and membrane complexes containing the erythrocyte receptor basigin^14,15^ is prevented. Indeed, both PfRH5 and basigin are targets of invasion-neutralizing antibodies^4,14,16,17^, and immunization with PfRH5 is protective in an *Aotus* model of malaria^12^ and delays the onset of symptoms in a human challenge model^13^. PfRH5 assembles with PfCyRPA and PfRIPR to form the tripartite PfRCR complex^7,8,18^. Both PfCyRPA and PfRIPR are also essential for erythrocyte invasion and are targets of invasion-blocking antibodies^5–7,19–21^. More recently a complex containing two merozoite surface proteins, PfCSS and PfPTRAMP, has been shown to bind to PfRCR and the structure of PfCSS was determined^1^. That RIPR, CSS and PTRAMP interact together was first identified in *Plasmodium knowlesi*, in which these proteins are also essential for invasion^22^. Invasion-neutralizing nanobodies have been identified against both PfCSS and PfPTRAMP^1^, and all five members of PfPCRCR are potential components of a blood-stage malaria vaccine.

Despite the essential function of each component of the PfRCR complex, their roles during invasion are enigmatic. Blocking the function of any member of PfPCRCR halts invasion of erythrocytes at the same step within the invasion process, preventing increase in calcium concentration at the parasite–erythrocyte interface, an event thought to occur before moving junction formation^1^. Several models for PfRH5 function have been proposed, including one that suggests that PfRH5 and PfRIPR undergo substantial conformational changes to insert into the erythrocyte membrane, forming pores^9^, and another that suggests that PfRH5 modulates signalling pathways leading to cytoskeletal changes during invasion^23^. However, neither of these hypotheses is robustly supported. To further understand the role of PfRCR in invasion, we therefore determined its structure at 3.0 Å resolution using cryogenic-electron microscopy (cryo-EM) and assessed the role of PfRH5 and PfRIPR in invasion.

## Structure of the PfRCR complex

**Fig. 1:**
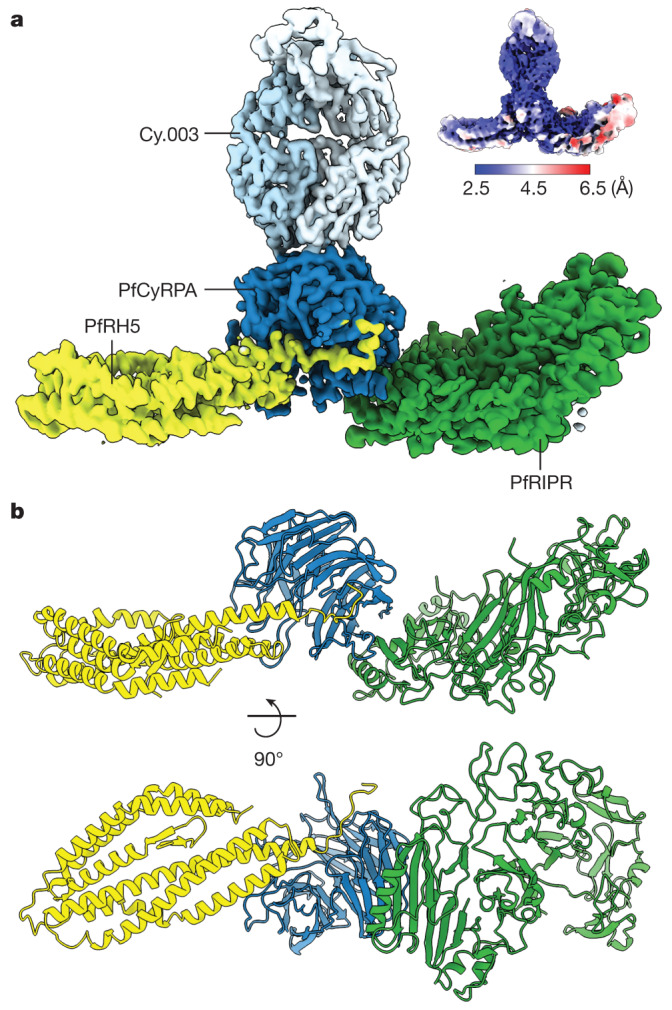
Structure of the PfRCR complex. **a**, Composite map of the PfRCR–Cy.003 complex, following local refinement of the consensus map and postprocessing with DeepEMhancer. Densities corresponding to PfRH5 (yellow), PfCyRPA (dark blue), PfRIPR (green) and Cy.003 (light blue) are highlighted. Inset at top right is the unsharpened consensus map before local refinement, coloured by local resolution. **b**, Structure of the PfRCR complex in cartoon representation, coloured as in **a**. Cy.003 is omitted for clarity.

**Extended Data Fig. 1:**
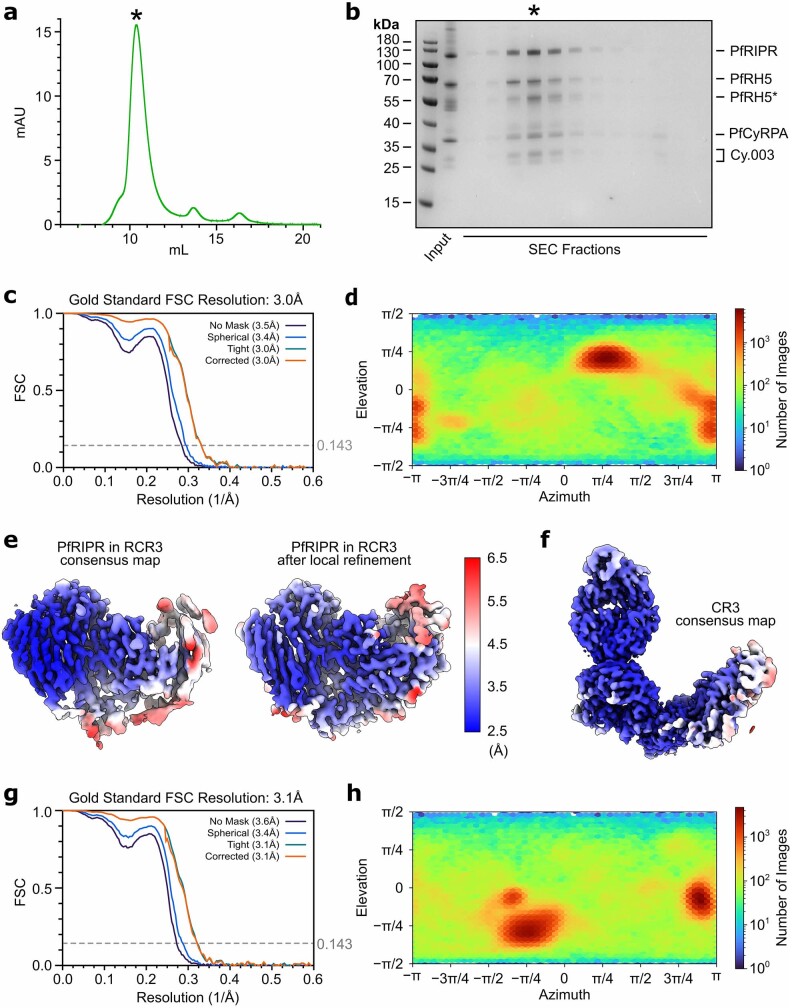
Purification of PfRCR-Cy.003 complex and cryo-EM processing statistics. **a**, Size exclusion chromatography profile and **b**, Reducing SDS-PAGE gel for the reconstituted PfRCR-Cy.003 complex, representative of five independent experiments. The fraction labelled with an asterisk corresponds to the region highlighted on the size exclusion chromatography profile in panel **a** containing the PfRCR-Cy.003 complex. Both full-length PfRH5 and truncated PfRH5 (PfRH5*) were observed in the purified complex. **c**, Gold-standard Fourier shell correlation curves of the consensus PfRCR-Cy.003 reconstruction. **d**, Particle view distribution of the consensus PfRCR-Cy.003 reconstruction. **e**, Map regions corresponding to PfRIPR in the consensus PfRCR-Cy.003 reconstruction and following local refinement, showing improvement in map quality. Both maps are coloured by local resolution and post-processed with DeepEMhancer. **f**, Consensus map of the PfCyRPA-PfRIPR-Cy.003 (CR3) complex, post-processed with DeepEMhancer, and coloured by local resolution as in (**e**). **g**, Gold-standard Fourier shell correlation curves of the consensus PfCyRPA-PfRIPR-Cy.003 reconstruction. **h**, Particle view distribution of the consensus PfCyRPA-PfRIPR-Cy.003 reconstruction. Source Data

**Extended Data Fig. 2:**
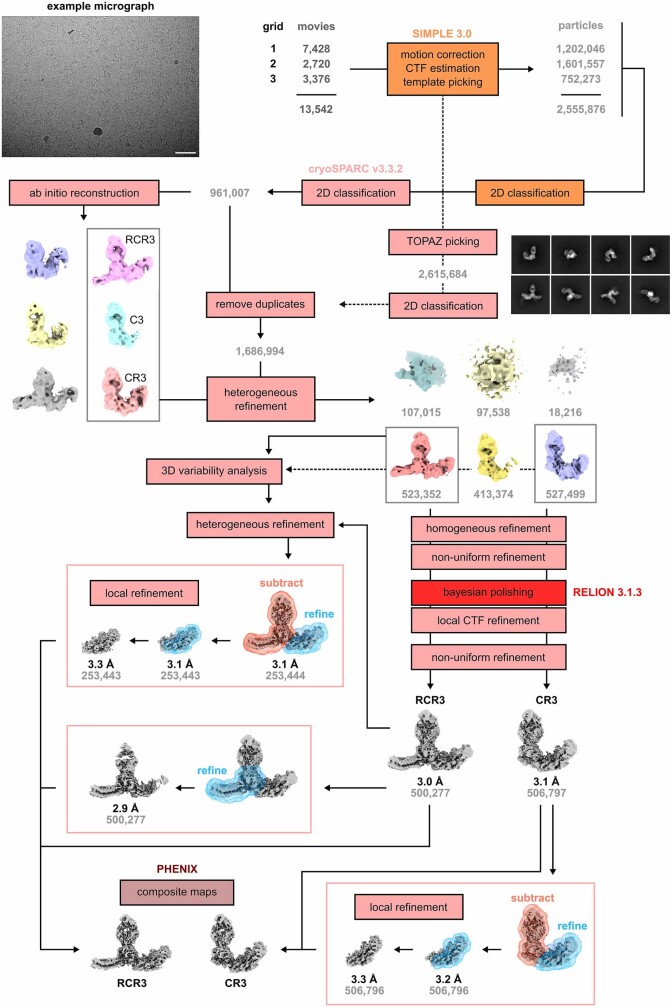
Workflow for cryo-EM data processing. Movies from three grids of the same PfRCR-Cy.003 sample were pre-processed separately in SIMPLE 3.0, followed by template-based particle picking in SIMPLE 3.0 and TOPAZ particle picking in cryoSPARC v3.3.2. After 2D classification and removal of duplicate particles, the cleaned particle stack was subject to heterogeneous refinement using maps for PfRCR-Cy.003 (RCR3), PfCyRPA-PfRIPR-Cy.003 (CR3) and PfCyRPA-Cy.003 (C3) complexes from *ab initio* reconstructions and three decoy volumes. Particle stacks corresponding to RCR3 and CR3 complexes were then separately refined in 3D space, then after Bayesian polishing in RELION 3.1.3 and local CTF refinement in cryoSPARC v3.3.2, subjected to a final non-uniform refinement to obtain consensus maps for each complex. Each map was further locally refined to improve the maps of PfRH5 and PfRIPR respectively using soft masks around the proteins of interest, and with signal subtraction for PfRIPR local refinements. Composite maps were generated using PHENIX. The micrograph shown is representative of 13,542 micrographs. The scale bar corresponds to 50 nm.

Although crystal structures are available for both PfRH5 (refs. ^3,4^) and PfCyRPA^5^, structural information for PfRCR is limited to a cryo-EM map of the complex at around 7 Å resolution^9^. Higher resolution was not achieved in this previous study because the PfRCR complex adopted preferred orientations when vitrified, preventing uniform imaging and reducing the resolution of the subsequent three-dimensional reconstruction. Crystal structures of PfRH5 and PfCyRPA could be docked into this map, providing insight into the architecture of PfRCR, but this yielded neither atomic-level information on the structure of PfRIPR nor the interactions among the PfRCR components. To provide an atomic resolution model of PfRCR we assessed the ability of Fab fragments from monoclonal antibodies, which bind to PfRH5 or PfCyRPA, to reduce the likelihood of PfRCR adopting preferred orientations. A complex of PfRCR bound to the Fab fragment of the invasion-neutralizing anti-PfCyRPA antibody Cy.003 (ref. ^5^) (Extended Data Fig. 1a,b) had a sufficiently uniform particle distribution to generate a consensus cryo-EM map with a global resolution of 3.0 Å (Fig. 1a, Extended Data Figs. 1c,d and 2 and Extended Data Table 1).

**Extended Data Fig. 3:**
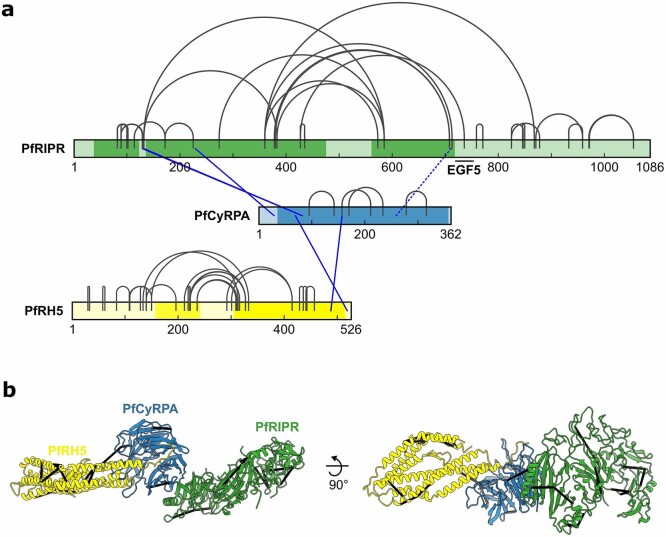
Crosslinking mass spectrometry of the PfRCR complex. **a**, Crosslinks found between PfRIPR, PfCyRPA and PfRH5 after incubating the reconstituted PfRCR complex with the crosslinker DSSO. Inter-protein crosslinks are blue and intra-protein crosslinks are grey. Crosslinks that are inconsistent with the PfRCR complex structure (with a Cα-Cα distance greater than 35 Å) are indicated by dashed lines. The regions of PfRIPR, PfCyRPA and PfRH5 built in the PfRCR structure are highlighted by a darker tone. The location of EGF5 in PfRIPR is indicated to highlight the intra-PfRIPR crosslink supporting the proposed location of this domain in the PfRIPR structure. Prepared using xiNET^50^. **b**, Crosslinks with a Cα-Cα distance less than 35 Å mapped on to the PfRCR complex structure.

Crystal structures of PfRH5 (ref. ^3^) and the PfCyRPA–Cy.003 Fab complex^5^ were docked into this map, confirming that PfCyRPA lies at the centre of PfRCR, bridging PfRH5 and PfRIPR (Fig. 1). This arrangement is also consistent with chemical crosslinks observed in crosslinking mass spectrometry (XL-MS) analysis of the PfRCR complex (Extended Data Fig. 3 and Extended Data Table 2). The region of electron density attributed to PfRIPR in this consensus map was not sufficiently large to accommodate the full-length molecule, suggesting that part of PfRIPR is flexibly attached to the ordered part of PfRCR, and was therefore not resolved. Local-resolution estimation indicated that PfCyRPA–Cy.003 was best resolved in the consensus map and PfRIPR the least well, especially at its PfCyRPA-distal edge (Fig. 1a).

To investigate the cause of the lower local resolution of PfRIPR, and to identify ways to improve map quality, we subjected particles of the PfRCR–Cy.003 complex to three-dimensional variability analysis in cryoSPARC^24^, showing conformational heterogeneity (Supplementary Video 1). Flexibility was observed along the length of PfRCR, with both PfRH5 and PfRIPR rocking relative to PfCyRPA and with further internal motion observed within PfRIPR, both consistent with observed local-resolution estimates (Fig. 1a). We therefore performed local refinement of both PfRH5 and PfRIPR, significantly improving the density for PfRIPR (Extended Data Figs. 1e and 2). A composite map generated by combining these global and local refinements was used to build a model of PfRCR (Fig. 1b and Extended Data Table 1). Chemical crosslinks identified through XL-MS analysis of the PfRCR complex are consistent with this model (Extended Data Fig. 3 and Extended Data Table 2).

**Extended Data Fig. 4:**
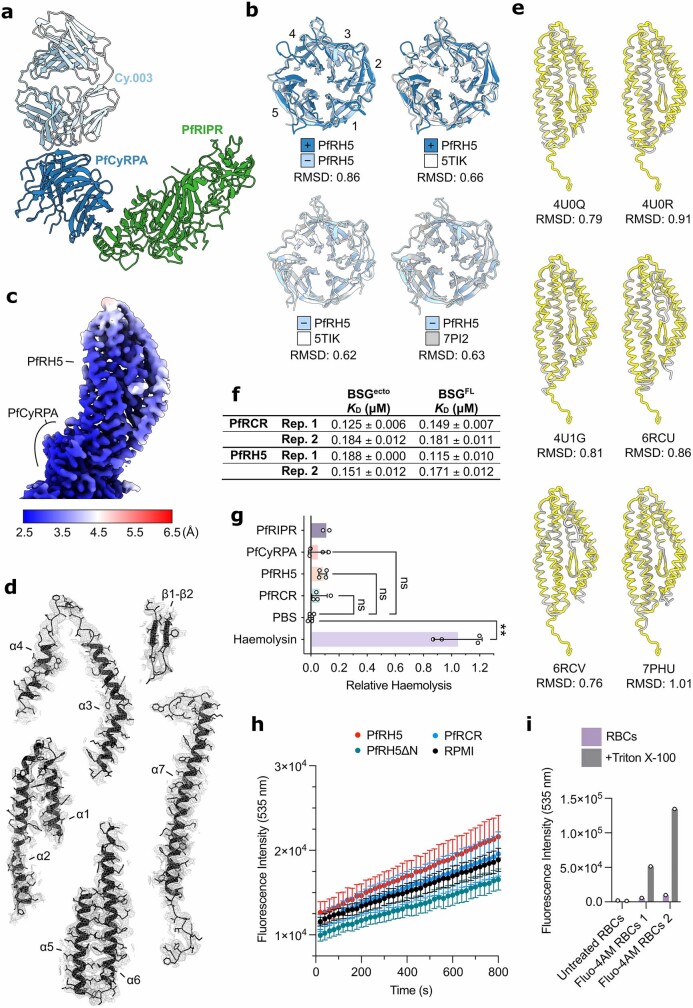
Comparison of PfRH5 and PfCyRPA structures and assessment of PfRH5 function. **a**, Structure of the PfCyRPA-PfRIPR-Cy.003 complex in cartoon representation, with PfRIPR (green), PfCyRPA (blue) and Cy.003 Fab (light blue) highlighted. **b**, Structural alignments of PfCyRPA from PfRCR-Cy.003 (dark blue), PfCyRPA-PfRIPR-Cy.003 (light blue), Cy.003-bound PfCyRPA (grey, PDB ID: 7PI2^5^), and unbound PfCyRPA (white, PDB ID: 5TIK^19^), with backbone RMSDs to each pair given below. Blades 1 through 5 are labelled. **c**, Cryo-EM map of PfRH5 following local refinement, post-processed by DeepEMhancer and coloured by local resolution. **d**, PfRH5 split into secondary structure elements, shown with their corresponding cryo-EM densities using the composite PfRCR-Cy.003 map post-processed with DeepEMhancer. **e**, Structural alignment of PfRH5 from PfRCR-Cy.003 (yellow) with crystal structures (grey) of basigin-bound (PDB ID: 4U0Q)^3^ or Fab-bound PfRH5 (PDB IDs: 4U0R, 4U1G, 6RCU, 6RCV, 7PHU)^3,4^. Backbone RMSDs between PfRCR-Cy.003 and each crystal structure are given below. **f**, Binding affinity measurements for PfRCR and PfRH5 binding to basigin ectodomain (BSG^ecto^) and full-length basigin (BSG^FL^) determined by microscale thermophoresis. Values determined from two independently prepared samples are provided. Each of these was measured three times. **g**, Relative haemolysis following the incubation of red blood cells with 2 µM of either PfPIPR, PfCyRPA, PfRH5, PfRCR or alpha-haemolysin, or with PBS alone. Only haemolysis induced by alpha-haemolysin is significantly greater than observed with PBS alone (p = 0.0016 for PBS vs Haemolysin, whereas p = 0.3207 for PBS vs PfRH5, p = 0.8673 for PBS vs PfRCR, and p > 0.9999 for PBS vs PfCyRPA, determined using a Kruskal-Wallis one-way ANOVA adjusted for multiple comparisons using Dunn’s test. N = 2 for PfRIPR (and therefore no statistical analysis has been performed) and n = 4 for all others. Individual data points, the mean +/− SD are shown. **h**, Raw fluorescence intensity measured at 535 nm after incubation of Fluo-4 loaded red blood cells (RBCs) with PfRH5, PfRH5ΔN, PfRCR, or RPMI alone. The mean +/− SEM for a technical triplicate are shown. **i**, Raw fluorescence intensity measured at 535 nm of untreated and Fluo-4AM treated RBCs, alone or following addition of 0.1% v/v Triton X-100, demonstrating successful loading of Fluo-4 (N = 1 for untreated RBCs and n = 2 for Fluo-4AM treated RBCs). Source Data

Despite isolation of an intact PfRCR–Cy.003 complex by gel filtration (Extended Data Fig. 1a,b), ab initio reconstructions showed that a second complex lacking PfRH5 was also present in the dataset (Extended Data Fig. 2). This was separately refined yielding a consensus map with a global resolution of 3.1 Å (Extended Data Fig. 1f–h). The map of this PfCyRPA–PfRIPR–Cy.003 complex also showed lower local resolution at the region corresponding to PfRIPR (Extended Data Fig. 1f), again consistent with the flexibility observed in three-dimensional variability analysis. We therefore built a model of this PfCyRPA–PfRIPR–Cy.003 complex using a composite map from consensus and local refinements (Extended Data Fig. 4a and Extended Data Table 3).

## Dynamics at the PfRH5–PfCyRPA interface

**Fig. 2:**
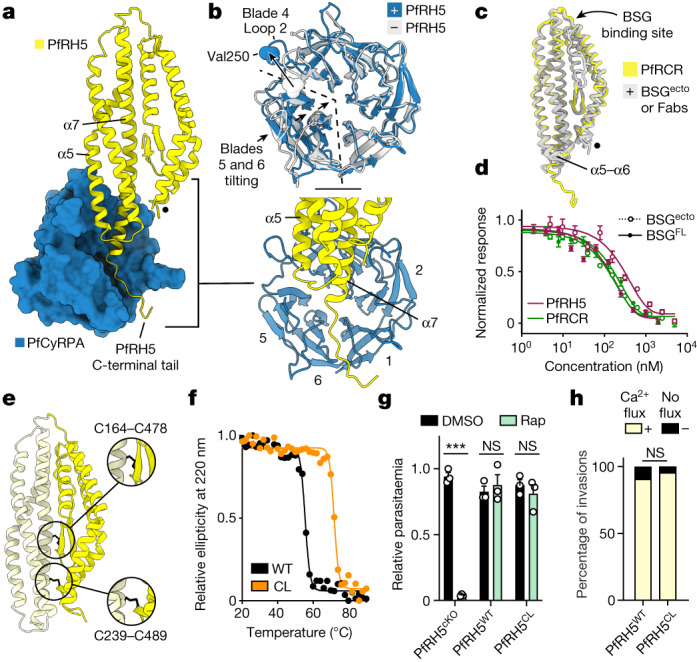
PfRH5 is a rigid component of PfRCR that does not open during invasion. **a**, The interface between PfRH5 (yellow cartoon) and PfCyRPA (blue surface). PfCyRPA blade 1 corresponds to Ile49–Lys95. The internal α2–α3 loop of PfRH5, unresolved in these data, is indicated by a black dot. Inset lower right, the interface between PfRH5 and PfCyRPA, with the blades of PfCyRPA labelled. **b**, Overlay of PfCyRPA from PfRCR–Cy.003- (blue) and Cy.003-bound PfCyRPA (grey; PDB: 7PI2)^5^, showing changes induced by PfRH5 binding. Val250 is shown as spheres. **c**, Overlay of PfRH5 from PfRCR–Cy.003 (yellow) and previous structures of PfRH5 bound to either BSG^ecto^ (PDB: 4U0Q)^3^ or anti-PfRH5 Fab fragments (PDBs: 4U0R, 4U1G, 6RCU, 6RCV and 7PHU)^3–5^ (grey). **d**, Microscale thermophoresis responses showing equivalent binding of BSG^ecto^ (dotted lines) and BSG^FL^ (full lines) to PfRH5 (maroon) and PfRCR (green); *n* = 2 biologically independent samples (one shown), both measured in triplicate. Mean plus or minus s.d. is shown. **e**, Model of the designed PfRH5^CL^ and its disulfide bonds (black), which lock together N-PfRH5 (dark yellow) and C-PfRH5 (light yellow). **f**, PfRH5^CL^ (orange) shows improved thermal stability compared with PfRH5^WT^ (black), as measured by circular dichroism. **g**, Conditional rapamycin (Rap)-induced knockout of PfRH5 (PfRH5^cKO^) reduced parasitaemia (***P < 0.001) but replacement with PfRH5^WT^ or PfRH5^CL^ did not (*P* = 0.599 and *P* = 0.457, respectively, two-tailed unpaired *t*-test); *n* = 3 biologically independent samples, each measured in triplicate. Mean plus or minus s.e.m. is shown. **h**, Percentage of invasion events by WT or PfRH5^CL^ parasites, showing a calcium flux (yellow) or no flux (black). No significant difference was observed (*P* = 0.68, two-tailed Fisher’s exact test); *n* = 41 invasion events for both PfRH5^WT^ and PfRH5^CL^ parasites from three biological replicates. NS, not significant. Source Data

To understand the interface between PfCyRPA and PfRH5 we first assessed the structure of PfRCR–Cy.003, comparing it with the crystal structures of PfRH5 and PfCyRPA—either free or in complex with ligands or antibodies (Fig. 2a,b and Extended Data Fig. 4). PfCyRPA and PfRH5 each have a buried surface area of approximately 1,720 Å^2^ with binding mediated by salt bridges, hydrogen bonds and hydrophobic interactions (Extended Data Table 4). No global conformational changes were observed in PfRH5 when compared with unbound PfRH5, with much of the PfRH5–PfCyRPA interface formed through rigid-body docking of blades 3 and 4 of the PfCyRPA β-propeller against helices α5 and α7 of PfRH5 (Fig. 2a). PfRH5 helix α7 projects towards the centre of the β-propeller of PfCyRPA, and the most substantial change observed in PfRH5 following PfCyRPA binding was an ordering of its C-terminal tail (residues 507–516). These residues follow α7 and were disordered in previous structures. In PfRCR these adopt an elongated conformation and occupy a groove that passes between blades 1 and 6 of PfCyRPA, pointing towards, but not contacting, PfRIPR.

A more substantial conformational change is observed in PfCyRPA when present in the PfRCR complex (Fig. 2b). Whereas blades 1–4 overlay closely with those in the crystal structures of PfCyRPA (root mean squared deviation (r.m.s.d.) 0.63 Å for Cy.003-bound PfCyRPA and 0.66 Å for unbound PfCyRPA (Fig. 2b and Extended Data Fig. 4b)), blades 5 and 6 and the linker between them move closer to the central axis of its β-propeller when in PfRCR, predominantly through a rigid-body tilt. This alters the width of the groove in which lie residues 507–516 of PfRH5. This is accompanied by a substantial and more localized rearrangement in loop 2 of blade 4, which moves by 11.5 Å at Val250 to accommodate helix α5 of PfRH5, with additional changes to loop 2 of each of blades 1–3 and to the linker between blades 3 and 4. To determine whether these changes were induced by PfRH5 binding, we compared the structure of PfCyRPA in PfRCR–Cy.003 with that in the PfCyRPA–PfRIPR–Cy.003 complex (Extended Data Fig. 4b). In this latter complex, PfCyRPA adopts a structure more like that observed in crystal structures (with r.m.s.d. of 0.63 Å for Cy.003-bound PfCyRPA and 0.62 Å for unbound PfCyRPA, versus 0.86 Å for PfCyRPA in PfRCR–Cy.003), indicating that these conformational changes are the result of PfRH5 binding. Notably, in both PfRH5 and PfCyRPA, the epitopes previously found to be the targets of neutralizing antibodies^3–5^ do not change in conformation following complex formation, supporting vaccine immunogen design approaches based on original crystal structures.

## PfRCR retains PfRH5 fold and function

Previous studies have suggested that PfRH5 is flexible, that its binding to basigin is influenced by its assembly into the PfRCR complex and that the N- and C-terminal halves of PfRH5 open to allow it to insert into the erythrocyte membrane to form a pore^9^. To test these hypotheses we started by assessing whether the structure of PfRH5 from our PfRCR–Cy.003 complex is different from previously determined PfRH5 structures. The global rigid-body motions along the length of the PfRCR complex (Supplementary Video 1) result in the distal tips of PfRCR appearing lower in resolution in the consensus PfRCR–Cy.003 cryo-EM map than the core of the complex (Fig. 1a). However, we hypothesized that this was an artefact of single-particle averaging for a flexible molecular assembly. Indeed, following local refinement to partially correct this flexibility, we found that the PfCyRPA-distal tip of PfRH5 in our locally refined map had a local resolution of about 4–5 Å, with higher resolution within its core (Extended Data Fig. 4c). This improved resolution of PfRH5 in the PfRCR complex allowed us to dock and refine a molecular model of PfRH5 more reliably (Extended Data Fig. 4d). Comparison of this refined model with the crystal structures of PfRH5, bound either to basigin^3^ or to monoclonal Fab fragments^3–5^, showed no consistent conformational changes in PfRH5 as a result of being part of PfRCR (with r.m.s.d. for each of less than 1 Å; Fig. 2c and Extended Data Fig. 4e). The only variable regions of PfRH5 across this alignment are the loop-linking helices α5 and α6 and the C-terminal end of α7, which are both within the region of PfRH5 that contacts PfCyRPA. The PfCyRPA-bound conformation is not an outlier when compared with other PfRH5 structures, suggesting that these are intrinsically flexible regions of PfRH5 that become ordered on binding to PfCyRPA. We therefore observed no conformational change in PfRH5 induced by assembly into PfRCR.

The finding that PfRH5 adopts a similar conformation in PfRCR as when bound to basigin^3^ led us to question a previous observation that PfRCR does not bind to the basigin ectodomain (BSG^ecto^)^9^. We therefore measured binding of PfRCR and PfRH5 to either BSG^ecto^ or detergent-solubilized full-length basigin (BSG^FL^) using microscale thermophoresis (Fig. 2d and Extended Data Fig. 4f). Binding was observed in each case, with affinities ranging from around 0.12 μM for PfRH5 binding to full-length basigin to about 0.19 μM for PfRH5 binding to BSG^ecto^, with the affinities for PfRCR binding to either form of basigin falling between these values (Extended Data Fig. 4f). We therefore find that PfRCR and PfRH5 bind to both basigin and its ectodomain with similar affinity, not supporting the hypothesis that assembly into the PfRCR complex changes the basigin-binding properties of PfRH5. Together with structure comparisons, these data show that assembly into the PfRCR complex does not affect the structure of PfRH5, nor its ability to bind to basigin.

## PfRH5 does not disassemble into a pore

**Extended Data Fig. 5:**
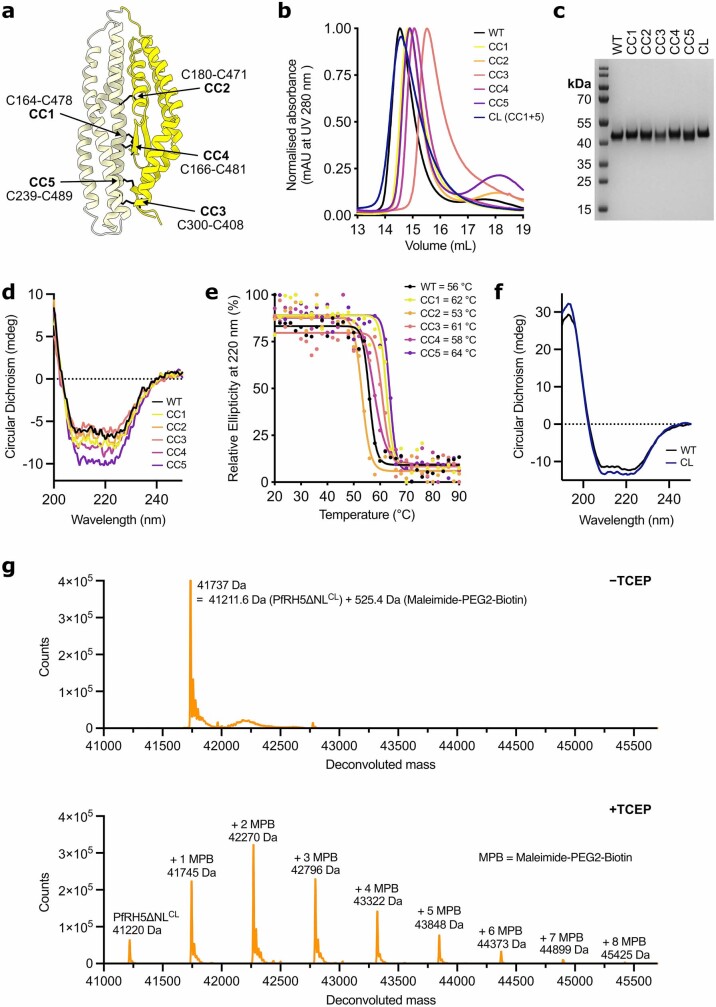
Design, expression and characterisation of cysteine-locked PfRH5. **a**, PfRH5 in cartoon showing the location of the five designed disulphide bonds (CC1 to CC5, black sticks), joining the N-terminal (dark yellow) and C-terminal (light yellow) halves of PfRH5. **b**, Gel filtration traces of purified wild-type PfRH5ΔNL (a construct lacking the unstructured N-terminus and α2-α3 loop of PfRH5^3^) and the cysteine-locked PfRH5 designs (CC1 to CC5) following expression in *Drosophila* S2 cells. **c**, SDS-PAGE gel of purified wild-type and single cysteine-locked variants (CC1 to CC5) and the double cysteine-locked (PfRH5^CL^, which combines crosslinks CC1 and CC5) version of PfRH5ΔNL. CC1 to CC5 were each expressed and purified once, while PfRH5^CL^ is representative of 3 independent samples. **d**, Single circular dichroism traces of wild-type and cysteine-locked variants of PfRH5ΔNL at 20 °C from their respective temperature ramps. **e**, Relative ellipticity at 220 nm versus temperature, and the calculated melting temperature of wild-type and cysteine-locked variants of PfRH5ΔNL. **f**, Circular dichroism traces of purified wild-type and the double cysteine-locked PfRH5ΔNL (PfRH5ΔNL^CL^). **g**, Intact mass spectrometry analysis of PfRH5ΔNL^CL^ following maleimide-PEG-biotin labelling in the absence (top) and the presence of TCEP (bottom). Unlabelled PfRH5ΔNL^CL^ has a theoretical mass of 41211.6 Da and +526 Da mass differences correspond to a maleimide-PEG2-biotin addition to cysteine residues. Source Data

Our structural studies do not rule out the possibility that the N- and C-terminal halves of PfRH5 might come apart during the erythrocyte invasion process, allowing PfRH5 to form a pore that causes a spike in calcium concentration at the merozoite–erythrocyte interface^9^. We therefore directly tested pore formation using a previously described haemolysis assay. In contrast to a previous study^9^, we observed no significant haemolysis in the presence of PfRH5, PfRIPR or PfRCR whereas alpha-haemolysin, a pore-forming toxin, caused considerable haemolysis (Extended Data Fig. 4g). Moreover, we did not observe an increase in calcium concentration within red blood cells following their incubation with PfRH5 or PfRCR (Extended Data Fig. 4h,i), which was described previously^21^. Having observed no evidence of pore formation in these in vitro assays, we next developed a parasite-based assay in which we introduced locking disulfide bonds into the centre of PfRH5 that would prevent the separation of its N- and C-terminal halves, and used a conditional replacement system to introduce these into a *P. falciparum* line to assess their effect on erythrocyte invasion (Fig. 2e–h). We employed a Rosetta^25^-based, structure-guided approach to design five disulfide bonds that we predicted would hold together the N- and C-terminal halves of PfRH5 (Cys164–Cys478 (CC1), Cys180–Cys471 (CC2), Cys300–Cys408 (CC3), Cys166–Cys481 (CC4) and Cys239–Cys489 (CC5)) (Extended Data Fig. 5a). In each case these were introduced into a version of PfRH5 lacking the N terminus and disordered loop (PfRH5ΔNL)^3^, were expressed in *Drosophila* S2 cells and were shown by circular dichroism analysis to adopt the expected secondary structure (Extended Data Fig. 5b–d). We used circular dichroism with a thermal melt to test the stability of these five variants and found that two of the designed disulfide bonds increased the thermal stability of PfRH5ΔNL by over 5 °C, as would be expected following successful formation of a stabilizing disulfide bond (+6 °C for CC1 and +8 °C for CC5; Extended Data Fig. 5e). These two disulfide bonds were then combined to generate a final cysteine-locked PfRH5 design (PfRH5^CL^; Fig. 2e and Extended Data Fig. 5c,f) and this combination increased the thermal stability of PfRH5ΔNL by around 14 °C, indicating that both designed cysteine locks had been formed (Fig. 2f). We further validated the presence of these disulfide bonds in PfRH5^CL^ by mass spectrometry analysis following maleimide-PEG2-biotin labelling^26^, which modifies free sulfhydryl groups but not disulfide-bonded cysteine residues. Non-reduced PfRH5ΔNL^CL^ was modified by the addition of just one maleimide, indicating that it contains only one free cysteine, most probably corresponding to the native single free cysteine, Cys239 (Extended Data Fig. 5g). By contrast, reduced PfRH5ΔNL^CL^ showed a series of species following labelling, containing from zero to eight maleimide modifications, consistent with a total of nine cysteine residues and partial labelling. Taken together, these data confirm that PfRH5^CL^ contains four disulfide bonds (two native and two designed) that are formed under non-reducing conditions.

**Extended Data Fig. 6:**
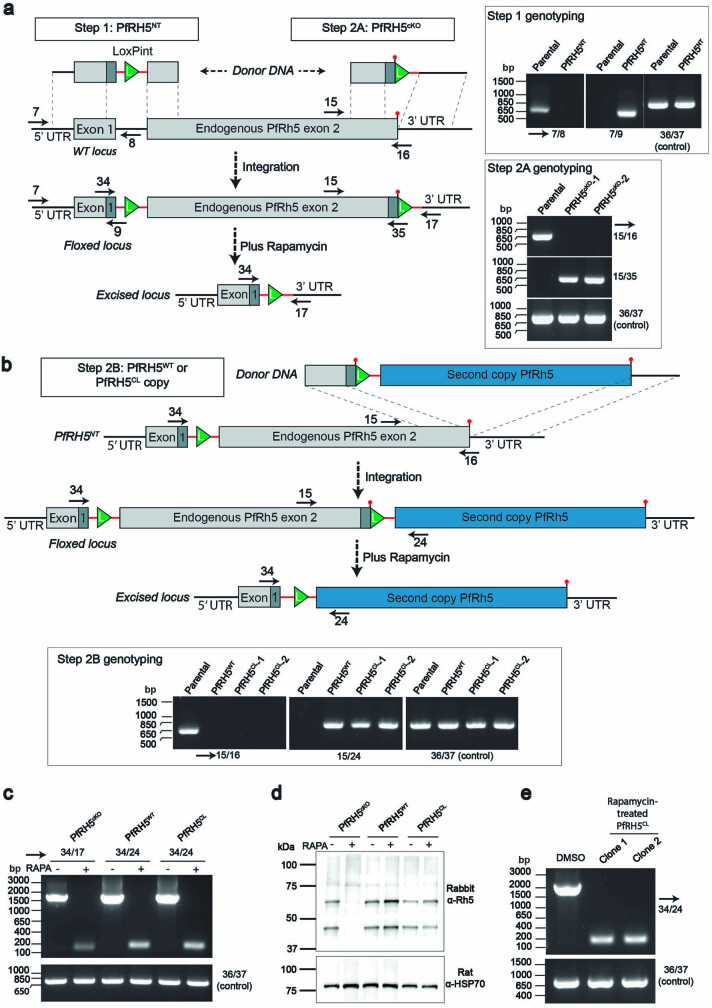
Generation of PfRH5 conditional knockout and complemented parasites. **a**, Schematic illustrating generation of transgenic rapamycin-inducible PfRH5^cKO^ line through the successive introduction of 5’ *LoxPint* (Step 1, generating line PfRH5^NT^) followed by 3’ *LoxPint* site (Step 2 A), resulting in floxed exon 2 of *PfRh5* (line PfRH5^cKO^). Genotyping PCRs are shown (inset on right). One clone of PfRH5^NT^ and two clones of PfRH5^cKO^ parasites were generated. Solid black arrows indicate position of primers used to screen by PCR for integration of *LoxP* sites, which was done once. Primers are numbered as in Supplementary Table 1. **b**, Schematic illustrating the introduction of a second, complementary copy of *PfRH5* (either PfRH5^WT^ or PfRH5^CL^) 3’ to endogenous *PfRH5*. The donor DNA included 548 bp of 3’ end of endogenous *PfRH5* followed by a small recodonised sequence, part of *LoxPint*, the second copy, and finally, 591 bp of endogenous 3’UTR. This design allowed for inducible complementation such that after rapamycin induced excision, only the complementary copy of *PfRH5* was expressed. PCR genotyping of Step 2B is shown in inset. One clone of PfRH5^WT^ and two clones of PfRH5^CL^ parasites were generated. For schematics in both **a** and **b**, green triangles represent *LoxP* DNA sequence; red lines represent *sera2* intron sequence; dark grey shaded boxes represent recodonised pieces of DNA. **c**, Genotyping PCR demonstrating that rapamycin induced excision of endogenous *PfRH5* exon 2. Data shown is representative of two biological repeats. **d**, Anti-PfRH5 western blotting demonstrating ablation of PfRH5 expression for rapamycin treated PfRH5^cKO^ parasites or expression of a complementary copy of *PfRH5* for PfRH5^WT^ and PfRH5^CL^ lines. Hsp70 was used as a loading control. Data shown is representative of three biological repeats. **e**, Rapamcyin treated PfRH5^CL^ parasites could be cloned by limiting dilution and maintained in culture. Primers used in PCR genotyping are listed in Supplementary Table 1 and the expected PCR product sizes in Supplementary Table 2. This was done once. For each set of PCR reactions, control primers 36/37 were used to amplify a 737 bp product from the *PfRON2* locus.

We next generated a transgenic *P. falciparum* line that could be used to induce a conditional replacement of the native PfRH5 gene with the cysteine-locked variant (Extended Data Fig. 6). To achieve this, *LoxP* sites were inserted silently within a synthetic intron^27^ either side of exon 2 of the endogenous PfRH5 gene, generating a conditional knockout line (PfRH5^cKO^) in which the addition of rapamycin would lead to excision of the coding sequence of the gene (Extended Data Fig. 6a). Two further strains were then generated in which a second copy of PfRH5 (either a wild-type (WT) sequence, PfRH5^WT^, or the cysteine-locked variant, PfRH5^CL^) was inserted downstream of the endogenous floxed PfRH5 gene such that the addition of rapamycin would lead to removal of the endogenous PfRH5 sequence and expression of the downstream copy (Extended Data Fig. 6b–e). We then tested the ability of all three lines to invade erythrocytes by treating ring-stage parasites with rapamycin or dimethylsulfoxide (DMSO) as a control, then measuring their resultant parasitaemia (Fig. 2g). The addition of rapamycin to PfRH5^cKO^, which lacks a downstream replacement copy of PfRH5, led to a reduction of over 95% in parasitaemia, confirming the requirement of PfRH5 for invasion. However, in parasite lines with a downstream copy of either PfRH5^WT^ or PfRH5^CL^ the addition of rapamycin did not affect parasitaemia, demonstrating that both were able to complement the deletion of PfRH5 and facilitate effective erythrocyte invasion. We next assessed whether the calcium flux that occurs at the merozoite–erythrocyte junction is also observed when PfRH5 is cysteine locked, and found no significant difference between the fraction of cells in which a calcium flux was observed during invasion events when comparing PfRH5^WT^ and PfRH5^CL^ lines (Fig. 2h and Supplementary Videos 2 and 3). The ability of a PfRH5 variant that is locked through two disulfide bonds across its N- and C-halves to mediate invasion is not compatible with a model in which the PfRH5 structure is required to ‘open’ such that it can insert into the erythrocyte membrane. Instead, this is consistent with PfRH5 adopting a rigid structure throughout the process of erythrocyte invasion with the calcium flux, which occurs downstream of PfRH5, not resulting from a PfRH5-mediated pore.

## The structure of PfRIPR

**Fig. 3:**
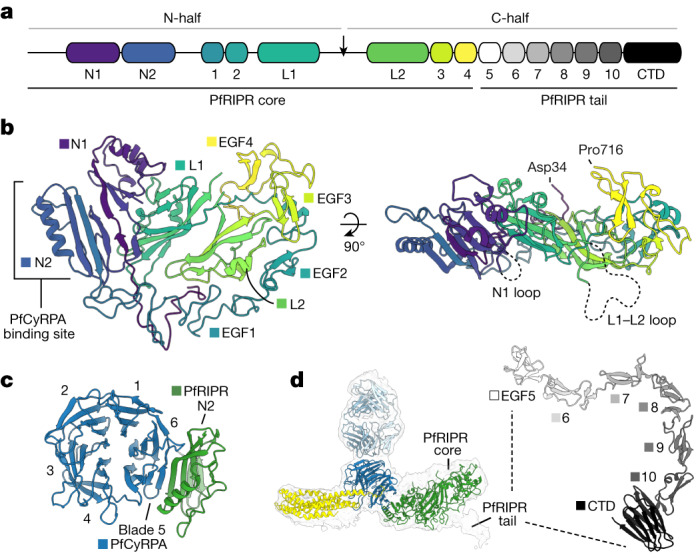
The structure of PfRIPR. **a**, The domain architecture of PfRIPR. The PfRIPR core comprises the N terminus to EGF-like domain 4, and its tail comprises EGF-like domain 5 to the C terminus. In addition to the ten previously known EGF-like domains, PfRIPR contains two N-terminal domains (N1 and N2), two lectin-like domains (L1 and L2) and a CTD. PfRIPR is cleaved at a site in the L1–L2 loop to produce its N- and C-halves, indicated by an arrow. **b**, Structure of the PfRIPR core (residues Asp34–Pro716) coloured sequentially from its N terminus (blue) to C terminus (yellow), as in **a**. The location of the PfCyRPA-binding site on domain N2, and the unresolved N1 loop (residues 124–137) and L1–L2 loop (residues 479–557), are shown. **c**, A view of the interface between PfCyRPA and PfRIPR, illustrating the extended β-sheet between PfCyRPA blade 5 and PfRIPR domain N2, plus some interactions with the helix of N2. Only domain N2 of PfRIPR is shown, for clarity. **d**, The 4.0 Å unsharpened map of the PfRCR–Cy.003 complex containing additional density for the beginning of the PfRIPR tail. The AlphaFold2 prediction of the PfRIPR tail is shown (right), with EGF-like domains and the CTD labelled and coloured in greyscale as in **a**. Source Data

**Extended Data Fig. 7:**
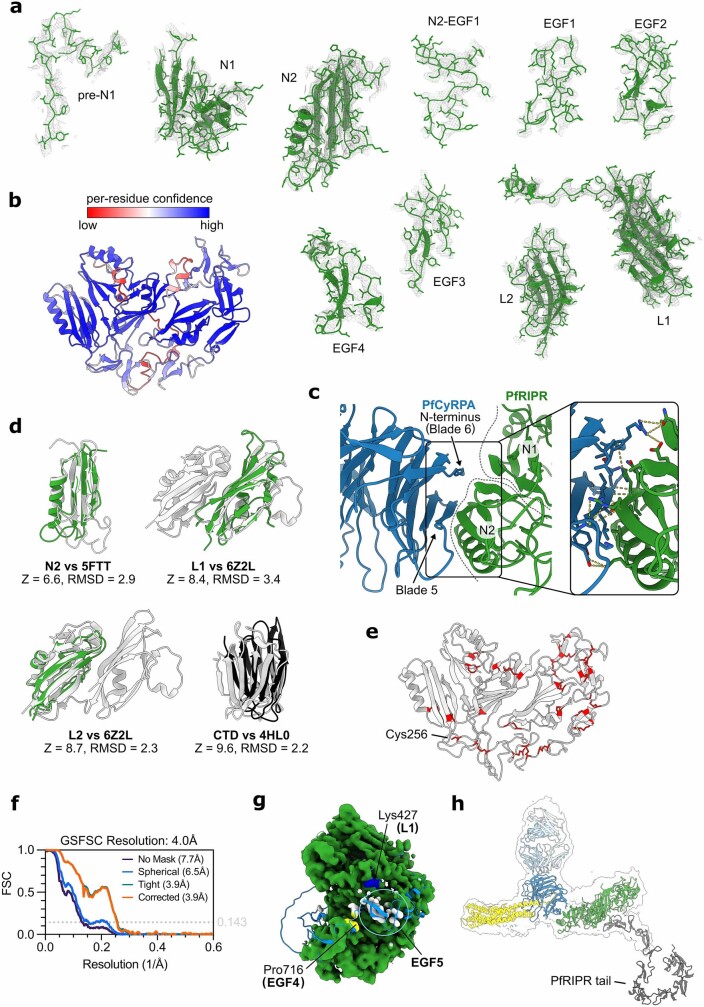
The PfRIPR core and generating a composite model of full-length PfRIPR. **a**, Domains of PfRIPR with their corresponding cryo-EM density using the PfRCR-Cy.003 composite map post-processed with DeepEMhancer. **b**, AlphaFold2 prediction of PfRIPR residues 19–716 (except 484–548) coloured by pLDDT score. The regions of low confidence in this model correlate with those that required de novo building guided by the AlphaFold2 prediction. **c**, The interface between PfRIPR and PfCyRPA comprises backbone and side chain interactions between residues of domains N1 and N2 of PfRIPR (green) and blades 5 and 6 of PfCyRPA (blue). Each are shown as cartoons with side chains of interacting residues (detailed in Extended Data Table 4) shown as sticks in the expanded section. Hydrogen bonds between residues are shown in yellow. **d**, Structural alignment of PfRIPR domains and their structural homologues with their DALI Z scores and RMSD scores. Structural homologues are identified by their PDB codes: 5FTT (lectin domain of Lactrophilin 3), 6Z2L (PfP113) and 4HL0 (Galectin). **e**, The PfRIPR core contains 23 disulphide bonds and one free cysteine (Cys256). Cysteine residues are shown as red sticks. **f**, Gold standard Fourier shell correlation (GSFSC) curves for the PfRCR-Cy.003 map containing additional density for the start of the PfRIPR tail. **g**, The PfRIPR portion of the composite PfRCR-Cy.003 map coloured green where a model of the PfRIPR core has been built. Uninterpretable density (coloured white) is observed in this map beyond domain EGF4 at Pro716 (yellow). An AlphaFold2 model of PfRIPR truncated after EGF5 (residues 20–769, light blue cartoon) predicts that this likely corresponds to EGF5. This is further supported by a chemical crosslink found between Lys736 of domain EGF5 and Lys427 of domain L1 (dark blue) in XL-MS analysis of the PfRCR complex (Extended Data Fig. 3). **h**, To generate a composite model of full-length PfRIPR, the AlphaFold 2 prediction of the PfRIPR tail (residues 717–1086) was docked onto the PfRIPR core using the 4.0 Å PfRCR-Cy.003 map containing additional PfRIPR density as a guide. Source Data

We next used our composite cryo-EM map of PfRCR–Cy.003 to build a molecular model of PfRIPR (Fig. 3). Map resolution was sufficient to build much of the structure de novo (Extended Data Fig. 7a), with molecular models derived from structure prediction, including those from a local installation of AlphaFold2 (ref. ^28^), used to guide building (Extended Data Fig. 7b), particularly in the less well-ordered regions of the map. This resulted in an almost complete model of residues 34–716 of PfRIPR (which has a total length of 1,086 residues), with no density observed for loops comprising residues 124–137 and 479–557 (Fig. 3). Moreover, we did not observe any interpretable density for residues 717–1,086 of PfRIPR, showing that this C-terminal region of PfRIPR is flexibly connected to the PfRCR complex. This is consistent with the internal crosslinks found within PfRIPR from XL-MS analysis of PfRCR, most of which were in the first 700 or so residues (Extended Data Fig. 3).

The structure of PfRIPR allowed us to redefine its domain architecture (Fig. 3a,b). PfRIPR has previously been described as consisting of two halves generated by cleavage of PfRIPR^29^, with these halves remaining associated^7^. Through sequence analysis, PfRIPR was also predicted to contain ten epidermal growth factor (EGF)-like domains, with two in the N-half (EGF1 and EGF2) and eight in the C-half (EGF3–EGF10)^7^. In our PfRIPR structure, the predicted cleavage site that joins the two predicted halves of PfRIPR is found within a large unstructured loop (residues 479–557). However, this loop does not link two separate halves of PfRIPR but instead emerges from within an ordered array of different domains, which ends after domain EGF4. We call this region the PfRIPR core.

The core of PfRIPR adopts a complex new architecture in which eight domains are intertwined (Fig. 3b). The two N-terminal domains, N1 and N2, each consist of a three- or four-stranded β-sheet packing against one or two α-helices. These two domains interact with one another through their β-sheets and it is through domain N2 that PfRIPR binds to PfCyRPA, with the three-stranded β-sheet of N2 forming a continuation of the β-sheet of PfCyRPA blade 5 (Fig. 3c). The interface between PfRIPR and PfCyRPA has a total buried surface area of roughly 1,060 Å^2^, formed predominantly through hydrophilic side chains, with contacts also being made between the long α-helix of N2 and the face of PfCyRPA blade 5, and between domain N1 and the N-terminal β-strand of PfCyRPA, which is found in blade 6 (Extended Data Fig. 7c and Extended Data Table 4). After a short sequence following N2, the first two EGF-like domains of PfRIPR, EGF1 and EGF2 follow, wrapping around one edge of the core. Next are two domains, L1 and L2, which form the centre of the PfRIPR core and around which the other domains fold. Intriguingly, L1 and L2 adopt the same fold as rhamnose-binding lectins and therefore the same fold as PfP113 (ref. ^30^) (Extended Data Fig. 7d), a proposed binding partner of PfRH5 that was previously thought to anchor PfRH5 to the merozoite surface^31^ but which was recently disputed as a surface protein^32^. The large disordered internal loop where PfRIPR is cleaved links L1 and L2. Multiple interdomain contacts within the PfRIPR core, including those between L1 and L2, hold the N- and C-halves of PfRIPR together following loop cleavage. Finally, EGF3 and EGF4 pack against the linker that joins EGF2 and L1, wrapping around the opposite edge of the PfRIPR core. This leaves the C terminus of the PfRIPR core at the side opposite to the PfCyRPA-binding site, and on the opposing face to N1 and the L1–L2 loop. Whereas the PfRIPR core is cysteine rich, containing 23 disulfide bonds and one free cysteine (Cys256), these are all contained within domains and there are no interdomain disulfide bonds (Extended Data Fig. 7e).

Whereas the map of PfRCR–Cy.003 did not contain density of sufficient resolution to allow model building of PfRIPR beyond residue 716, in several two-dimensional classes we could see weaker additional density projecting away from the PfRIPR core (Extended Data Fig. 2). This density could not be improved or extended using two-dimensional classification with a larger particle box size, suggesting that it results from residues flexibly linked to the PfRIPR core. Nonetheless, non-uniform refinement of a subset of particles yielded a map at a global resolution of 4.0 Å (Fig. 3d and Extended Data Fig. 7f), which showed that this extra density projects away from the centre of the PfRIPR core, adjacent to where EGF4 ends at residue 716. This suggested that the remainder of PfRIPR extends away from the PfRIPR core at this location. Indeed, XL-MS analysis identified a crosslink between Lys736 of EGF5 and Lys427 of domain L1, indicating that EGF5 is located approximately in this area (Extended Data Fig. 3 and Extended Data Table 2) and as supported by an AlphaFold2 prediction of PfRIPR up to the end of EGF5 (Extended Data Fig. 7g). To generate a complete composite model of full-length PfRIPR we used AlphaFold2 to predict the structure of residues 717–1,086 of PfRIPR. This predicts an elongated but ordered structure that we call the PfRIPR tail, consisting of EGF-like domains 5–10 followed by a C-terminal domain (CTD) with a galectin-like fold (Fig. 3d and Extended Data Fig. 7d). Guided by the additional density in the 4.0 Å map, we docked this model of the PfRIPR tail onto the PfRIPR core to generate a complete molecular model for PfRIPR (Extended Data Fig. 7h).

## The PfRIPR tail binds PfCSS–PfPTRAMP

**Fig. 4:**
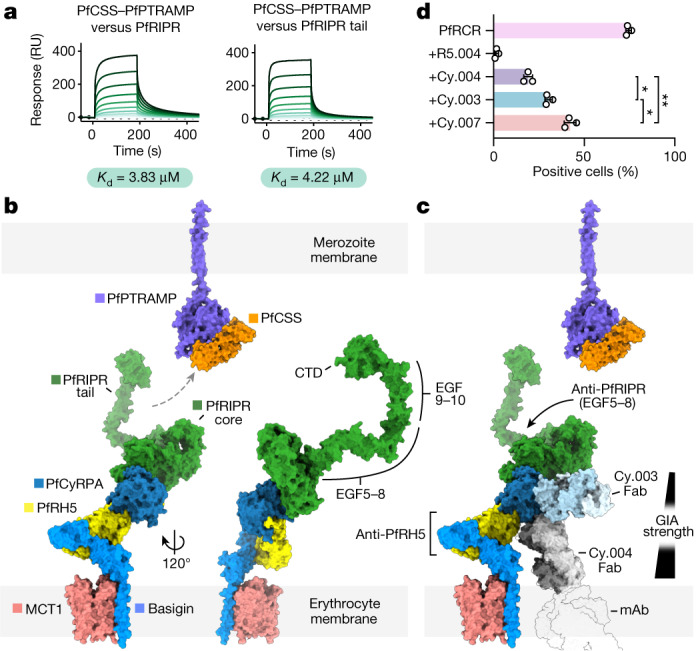
The PfPCRCR complex bridges the parasite and erythrocyte membranes. **a**, The PfCSS–PfPTRAMP complex binds to full-length PfRIPR and its tail with equal affinity, as measured by surface plasmon resonance; *n* = 4 independent experiments (one shown). RU, response units. The binding affinity given is the mean of those determined by steady-state analysis of each independent measurement (Extended Data Fig. 8d). **b**, Composite model of the PfRCR complex on the erythrocyte membrane, illustrating how the tail of PfRIPR projects towards the merozoite membrane where PfCSS–PfPTRAMP is located. EGF-like domains 5–8 and 9–10 and the CTD of PfRIPR are highlighted. The composite PfRCR complex model is aligned onto the structure of basigin-bound MCT1 (PDB: 7CKR^33^). For illustrative purposes only, the AlphaFold2-predicted structure of PfPTRAMP (magenta, AlphaFoldDB: Q8I5M8, residues 26–352) and the crystal structure of PfCSS (orange, PDB: 7UNY^1^) are docked together. **c**, Mapping of growth-inhibitory antibodies targeting PfRCR components in the context of erythrocyte binding. The potency of growth-inhibitory PfCyRPA-targeting antibodies correlates with their proximity to the erythrocyte membrane, illustrated by overlay of Cy.004 Fab-bound PfCyRPA (PDB: 7PHW^5^) onto PfRCR–Cy.003. The approximate location of the Fc portion of the intact monoclonal antibody is shown as a faint silhouette through overlay of the crystal structure of an intact antibody (PDB: 1IGT). Growth-inhibitory PfRIPR-targeting antibodies bind to EGF-like domains 5–8, located distal to the erythrocyte membrane. **d**, The ability of PfRCR to bind to human erythrocytes, either alone or in the presence of twofold molar excess of antibodies R5.004, Cy.003, Cy.004 and Cy.007, expressed as a percentage of PfRCR-positive cells; *n* = 3 independent measurements; mean plus or minus s.d. is shown; *P* = 0.0132 for Cy.003 versus Cy.004, *P* = 0.0418 for Cy.003 versus Cy.007 and *P* = 0.0023 for Cy.004 versus Cy.007 (one-way Brown–Forsythe and Welch analysis of variance adjusted for multiple comparisons with Dunnett T3). Source Data

**Extended Data Fig. 8:**
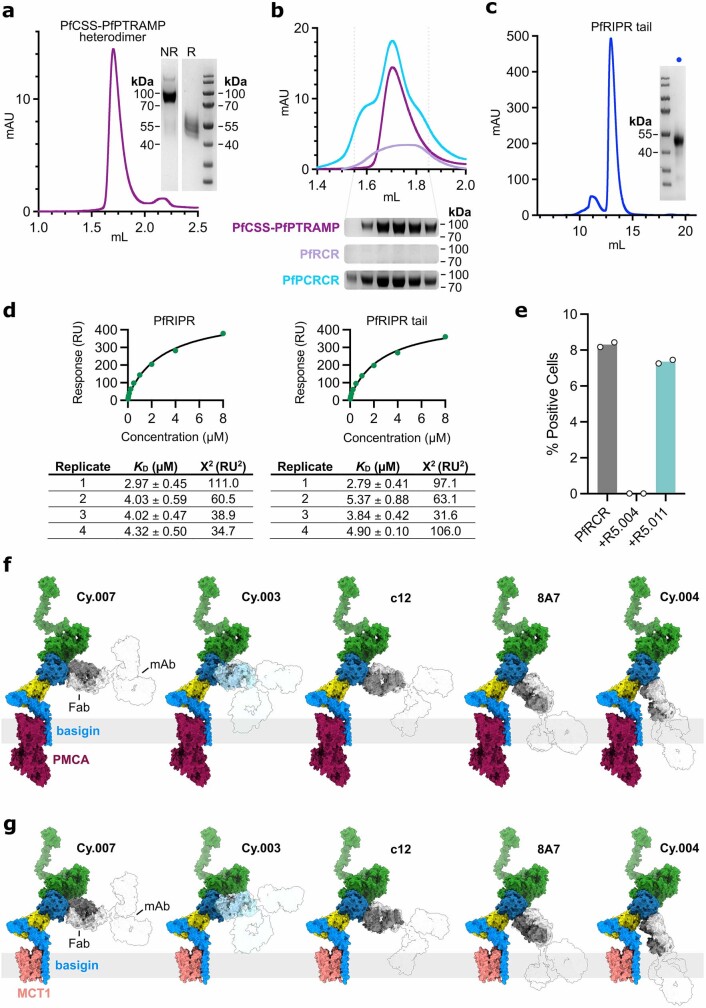
PfRIPR tail interacts with the PfCSS-PfPTRAMP complex, and the mapping of anti-PfCyRPA antibodies. **a**, Gel filtration trace of the PfCSS-PfPTRAMP heterodimer with an SDS-PAGE gel inset right. SDS-PAGE fractions are shown for non-reduced (NR) and reduced (R) samples of the disulphide-linked heterodimer. Representative of three independent experiments. **b**, Gel filtration traces for PfCSS-PTRAMP complex (dark purple), PfRCR (light purple) and the mixture of the two complexes (blue). Underneath non-reducing SDS-PAGE gels show the corresponding fractions for the location of PfCSS-PfPTRAMP from the three experiments. Traces and gels are representative of two independent analyses. **c**, Gel filtration trace of recombinantly expressed PfRIPR tail with reducing SDS-PAGE gel of isolated PfRIPR tail shown inset right, representative of three independent samples. **d**, Representative steady state fit analysis of PfCSS-PfPTRAMP complex binding to full-length PfRIPR and PfRIPR tail by SPR. Binding analysis was completed four times. Inset below are the estimated binding affinity (*K*_D_) and the Chi^2^ fit for each repeat. **e**, FACS-based analysis of the binding of PfRCR complex to human erythrocytes in the presence of PfRH5-targeting antibodies R5.004 and R5.011. In each case, PfRCR was detected using an anti-C-tag nanobody targeting the affinity tag of the complex. Binding of the PfRCR complex is unaffected by the presence of R5.011. N = 2, individual data points, the mean +/- SD are shown. **f**, Composite model showing structural alignment of anti-PfCyRPA antibodies on to the PfRCR complex when bound to PMCA-bound basigin on the erythrocyte surface, with the rest of the antibodies modelled as faint silhouettes. Generated through alignment of the PfRCR-Cy.003 complex, Fab-PfCyRPA crystal structures (PDB IDs: 7PHV, 5EZO, 5TIH and 7PHW)^5,19,20^, an intact IgG crystal structure (PDB ID: 1IGT), and PMCA-bound basigin (PDB ID: 6A69^34^). **g**, As in (**f**) except when PfRCR is bound to MCT1-bound basigin (PDB ID: 7CKR^33^). Source Data

The *P. knowlesi* homologues of RIPR, PkRIPR and, more recently, PfRIPR itself have been shown to bind their respective homologues of the CSS–PTRAMP complex^1,22^. However, the binding interface between these proteins is undefined. We first confirmed that the PfCSS–PfPTRAMP heterodimer could assemble with the PfRCR complex into a five-component complex (Extended Data Fig. 8a,b). Given that our structure shows that PfRIPR comprises two distinct regions, we next asked whether it is the core or the tail of PfRIPR that mediates binding to PfCSS–PfPTRAMP. We immobilized full-length PfRIPR and the PfRIPR tail (Extended Data Fig. 8c) on two separate flow paths of a surface plasmon resonance chip then flowed the PfCSS–PfPTRAMP heterodimer over these surfaces. Both PfRIPR and its tail bound to PfCSS–PfPTRAMP with a similar affinity of about 4 μM (Fig. 4a and Extended Data Fig. 8d), indicating that the PfRIPR tail alone is sufficient for binding to the transmembrane-anchored PfCSS–PfPTRAMP complex. This arrangement positions the complex containing the PfRIPR core, PfCyRPA and PfRH5 such that it can bind to receptors such as basigin on the erythrocyte surface.

## Mechanisms of neutralizing antibodies

Invasion-neutralizing monoclonal antibodies have been identified against each of PfRH5, PfCyRPA and PfRIPR. To understand how these antibodies block the invasion process, we mapped their binding sites onto a model of PfRCR. Because erythrocyte basigin is found in a stable complex with either plasma membrane calcium transporters (PMCAs) or the monocarboxylate transporter (MCT1)^15^, we combined our structure of PfRCR with that of the PfRH5–basigin complex^3^ and with structures of basigin–MCT1 (ref. ^33^) and basigin–PMCA^34^ to generate composite models of the PfRCR complex on the erythrocyte surface (Fig. 4b and Extended Data Fig. 8f,g). Whereas there will be some flexibility in the presentation of the PfRCR complex due to hinge movements in basigin, modelling predicts that PfRCR will project away from the erythrocyte surface, with PfRH5 closest to the erythrocyte and with the PfRIPR tail projecting towards the merozoite surface. This arrangement is consistent with surface plasmon resonance binding data (Fig. 4a), which indicates that the PfRIPR tail mediates binding to PfCSS–PfPTRAMP.

Neutralizing antibodies targeting PfRH5 and PfCyRPA are proposed to act by sterically blocking the approach of PfRCR to the erythrocyte membrane, preventing it from binding to basigin and any potentially unknown surface receptors^5,6^. Indeed, the most effective PfRH5-binding neutralizing antibodies block PfRH5 from binding to basigin–PMCA and basigin–MCT1 complexes^15^. There is no known erythrocyte-binding partner for PfCyRPA, and the mechanism by which invasion-blocking, anti-PfCyRPA antibodies act is uncertain. Here we find that, when aligned onto the composite model of PfRCR–basigin–MCT1 and PfRCR–basigin–PMCA, the degree to which these antibodies project towards the erythrocyte membrane correlates with their growth-inhibitory activity^5^ (Fig. 4c and Extended Data Fig. 8f,g). Aligning the crystal structure of PfCyRPA bound to the strongly neutralizing antibody Cy.004 (ref. ^5^) onto PfRCR places the Fab fragment of Cy.004 projecting towards the erythrocyte surface, where it would sterically clash with the membrane when part of an intact monoclonal antibody. By contrast, the less-neutralizing antibodies Cy.003, Cy.007, 8A7 and c12 project towards the erythrocyte membrane to a lesser degree. The invasion-blocking activity of these antibodies^5^ thus correlates with the degree to which they project towards the erythrocyte membrane.

To test the hypothesis that PfCyRPA-binding antibodies sterically block binding of PfRCR to erythrocytes, we developed a flow cytometry-based assay. To detect binding of PfRCR to erythrocytes we first used a fluorescently labelled anti-C-tag nanobody that targets the affinity purification tag of the complex (Supplementary Fig. 2a,b). We observed that PfRCR bound to human erythrocytes and that the anti-PfRH5 neutralizing antibody R5.004 (ref. ^4^) blocked this binding but that the non-neutralizing antibody R5.011 (ref. ^4^) did not (Extended Data Fig. 8e). We therefore used R5.011 as the primary detection antibody in a second round of flow cytometry measurements, allowing signal amplification (Supplementary Fig. 2c). Here, following incubation of erythrocytes with PfRCR, we observed that around 74% of these were positively labelled (Fig. 4d). As before, R5.004, which directly prevents PfRH5 from binding to basigin^4^, ablated erythrocyte binding. Each of the growth-neutralizing, PfCyRPA-targeting antibodies—Cy.003, Cy.004 and Cy.007 (ref. ^5^)—also reduced binding of PfRCR to erythrocytes (Fig. 4d and Supplementary Fig. 2d). Moreover, Cy.004, the most growth-neutralizing of these three antibodies^5^, caused the greatest reduction in PfRCR binding, consistent with it projecting most directly towards the erythrocyte membrane as predicted from composite modelling (Fig. 4c). This supports a model in which PfCyRPA-targeting antibodies inhibit growth by sterically blocking the binding of PfRCR to the erythrocyte surface.

There are currently few studies detailing growth-inhibitory antibodies that target PfRIPR. The most effective known polyclonal and monoclonal antibodies targeting PfRIPR bind to EGF-like domains 5–8 in the PfRIPR tail^6,7,21^. How these antibodies block the invasion process will require further study but, because the tail of PfRIPR binds to PfCSS–PfPTRAMP, it is possible that they may affect processes such as receptor engagement on the merozoite surface rather than on the erythrocyte surface. Our knowledge of the structure of PfRIPR and how it assembles into the PfPCRCR complex will now allow future studies to assess the mechanisms of action of these PfRIPR tail-targeting antibodies. Future studies may also identify growth-inhibitory antibodies that bind the PfRIPR core, which have thus far been missed owing to small antibody panel sizes. Our structure will now enable a programme of structure-guided vaccine design based around PfRIPR to specifically induce and analyse such antibodies. For example, structure-guided approaches can be used to produce individual PfRIPR domains, resurfacing them to facilitate recombinant expression out of the context of the rest of PfRIPR. These could be used to raise antibodies towards specific regions of PfRIPR that may point towards the erythrocyte membrane, towards basigin or towards the PfCSS–PfPTRAMP complex on the merozoite surface. This will allow rational probing of the growth-neutralizing effects of antibodies targeting different surfaces of PfRIPR, showing its antigenic cartography and allowing rational design of immunogens that specifically elicit the most effective antibodies.

## Conclusions

Our cryo-EM structure of PfRCR has allowed us to build an atomic model of PfRIPR, showing it to consist of a complex multidomain core flexibly attached to an elongated tail and containing domains that adopt the same fold as galectin- and rhamnose-binding lectins. We demonstrate the conformations of PfRH5, PfCyRPA and PfRIPR when part of PfRCR and define their interactions at the molecular level. We find that PfRH5 does not differ substantially in structure when integrated into the PfRCR complex, and that disulfide locking of the two halves of PfRH5 together has no impact on erythrocyte invasion, indicating that PfRH5 does not open to insert into the erythrocyte membrane as part of the invasion process. Calcium fluxes that occur during invasion are also unaffected by locking of PfRH5. Additionally, we find that the tail of PfRIPR binds to the PfCSS–PfPTRAMP complex, which is anchored at the merozoite surface, suggesting that PfRCR bridges both erythrocyte and parasite membranes during invasion. The mapping of growth-neutralizing antibodies onto the structure of erythrocyte-bound PfRCR supports a model in which antibodies that target PfRH5 and PfCyRPA sterically prevent PfRCR from binding to the erythrocyte membrane, which we confirm using a fluorescent activated cell sorting-based assay. By contrast, the most effective PfRIPR-targeting, invasion-neutralizing antibodies may act towards the parasite membrane. These studies inform and open new future avenues to understanding the molecular mechanisms of erythrocyte invasion and the rational design of blood-stage malaria vaccines.

## Methods

### Protein expression and purification

PfRH5 (residues E26–Q256, with substitutions C203Y (of the 7G8 *P. falciparum* strain) and T216A and T299A (to remove potential N-linked glycosylation sites), a BiP secretion sequence and a C-terminal C-tag), was expressed from a stable S2 cell line^35^ (ExpreS^2^ion Biotechnologies) in EX-CELL 420 Serum Free Medium (Sigma-Aldrich). After 3–4 days, culture supernatants were harvested and 0.45 µm filtered then incubated with CaptureSelect C-tagXL resin (Thermo Scientific). Beads were washed using 20–30 column volumes of Tris buffered saline (TBS) (25 mM Tris pH 7.5, 150 mM NaCl) and bound proteins eluted with C-tag elution buffer (25 mM Tris pH 7.5, 2 M MgCl_2_). Eluted proteins were further purified by gel filtration using an S200 Increase 10/300 column with a running buffer of HEPES buffered saline (HBS) (20 mM HEPES pH 7.5, 150 mM NaCl). PfRH5ΔN^3^ was also expressed from a stable S2 cell line and was purified as for full-length PfRH5 except that HBS containing 5% v/v glycerol was used as a running buffer for gel filtration.

PfCyRPA (residues D29–E362, with substitutions S147A, T324A and T340A (to remove potential N-linked glycosylation sites), a mammalian secretion sequence and a C-terminal C-tag) was transiently expressed using Expi293F cells with the Expi293 Expression System Kit (Thermo Fisher) as recommended by the manufacturer. Culture supernatants were harvested and 0.45 µm filtered, diluted 1:1 in TBS and then incubated with CaptureSelect C-tagXL resin. Purification then proceeded as for PfRH5.

PfRIPR (residues D21–N1086, with substitutions N103Q, N144Q, N228Q, N303Q, N334Q, N480Q, N498Q, N506Q, N526Q, N646Q, N647Q, N964Q and N1021Q (to remove potential N-linked glycosylation sites), a BiP secretion sequence and a C-terminal C-tag) was expressed from a stable S2 cell line as for PfRH5. After 3–4 days, culture supernatants were harvested and 0.45 µm filtered before being concentrated and buffer exchanged into 50 mM Tris pH 7.5 and 150 mM NaCl by tangential flow filtration with a stack of three 100 kDa Omega Cassettes (PALL Corporation). The exchanged supernatant was loaded onto a 1 ml prepacked CaptureSelect C-tagXL column (Thermo Scientific) equilibrated with TBS. Following washing with 30–50 column volumes of TBS, bound proteins were eluted with C-tag elution buffer. Eluted proteins were exchanged into HBS using a PD-10 desalting column (Cytiva) then either further purified by gel filtration as for PfRH5 or used directly for PfRCR complex preparation.

PfRIPR tail (residues D717–N1086, with substitutions N964Q and N1021Q (to remove potential N-linked glycosylation sites), a BiP secretion sequence and a C-terminal Avi-C-tag) was also expressed from a stable S2 cell line. PfRIPR tail was purified as for full-length PfRIPR except that filtered culture supernatants were exchanged into TBS by tangential flow filtration with a stack of three 5 kDa Omega Cassettes (PALL Corporation). Eluted proteins were further purified by gel filtration using an S200 Increase 10/300 column into HBS.

The PfCSS–PfPTRAMP heterodimer was obtained by coexpression of PfCSS (residues Q21–K290, with a secretion sequence and C-terminal His6-tag) and PfPTRAMP (residues C30–T307, with a secretion sequence and C-terminal His6-tag) using FreeStyle 293-F cells (Thermo Fisher) in FreeStyle F17 Expression Medium supplemented with l-glutamine and 1× MEM non-essential amino acids (Gibco). Six days following transfection, culture supernatants were harvested, 0.45 µm filtered then incubated with Super Ni-NTA resin (Generon) equilibrated in HBS. Following washing with 20 column volumes of HBS supplemented with 20 mM imidazole, bound proteins were eluted with HBS supplemented with 300 mM imidazole then further purified by gel filtration using an S200 Increase 10/300 column into HBS.

Cy.003, Cy.004, Cy.007 (ref. ^5^), R5.004 and R5.011 (ref. ^4^) were transiently expressed in Expi293F cells. Culture supernatants were harvested and 0.45 µm filtered before loading onto a 1 ml HiTrap Protein G HP column (Cytiva) pre-equilibrated in PBS (no. 79382, Sigma-Aldrich). The column was washed with PBS and bound proteins were eluted using 5 ml of 0.1 M glycine pH 2.5 into 1 ml of 1 M Tris pH 8.0. Eluted monoclonal antibodies (mAbs) were exchanged into PBS.

To prepare Fab fragments of Cy.003, the mAb was cleaved using Immobilized Papain (no. 20341, Thermo Scientific). Following cleavage, Fc and Fab fragments were separated using a 1 ml HiTrap rProtein A prepacked column (Cytiva) and the unbound fraction containing Cy.003 Fab was exchanged into PBS.

To express full-length human basigin, a synthetic gene (UniProt ID P35613-2) was cloned into pFastBac with a C-terminal His6-tag for expression in Sf9 insect cells using the Bac-to-Bac Baculovirus Expression System (Thermo Fisher). Following transformation of DH10Bac cells, bacmids were isolated by isopropanol precipitation and used to transfect Sf9 cells at a cell density of 1 million per ml in Sf-900 II serum-free medium (Gibco). First-generation baculoviruses (P1) were amplified to produce second-generation baculoviruses (P2), which were then used to induce expression of full-length basigin by addition at 1% v/v to Sf9 cells at roughly 2.5–3.0 million cells per ml. After 48 h, cells were harvested then resuspended in lysis buffer (25 mM Tris pH 8.0, 150 mM NaCl, 10% glycerol) supplemented with cOmplete EDTA-free protease inhibitors (Roche). Cells were lysed using a Dounce homogenizer followed by sonication on ice (60% amplitude, 3 s pulse, 9 s rest for 1.5 min). Lysed homogenate was clarified by centrifugation at 3,000*g* for 20 min, then the supernatant further spun at 100,000*g* for 45 min at 4 °C to isolate membranes. Following resuspension in lysis buffer, membranes were solubilized with 1.2% dodecyl-β-d-maltoside (DDM) for 1 h at 4 °C. Following centrifugation at 100,000*g* for 45 min at 4 °C, the supernatant with incubated with Ni-NTA resin (Qiagen) at 4 °C for 1 h and then the resin was washed (25 mM Tris pH 8.0, 300 mM NaCl, 10% glycerol, 0.02% DDM and 15 mM imidazole). Bound proteins were eluted using 25 mM Tris pH 8.0, 150 mM NaCl, 10% glycerol, 0.02% DDM and 400 mM imidazole then further purified on an S200 Increase 10/300 column into buffer containing 20 mM HEPES pH 7.2, 150 mM NaCl and 0.02% DDM:cholesteryl hemisuccinate (10:1 w/v ratio). Approximately 100–200 µg of full-length basigin was obtained from 100 ml of Sf9 cell culture.

Basigin ectodomain was expressed and purified as reported previously^3^.

### Structure determination using cryo-EM

To prepare a complex of PfRH5–PfCyRPA–PfRIPR–Cy.003 Fab for cryo-EM analysis, purified proteins were incubated together at an equimolar ratio in HBS for 5 min at room temperature. Approximately 250 µg of complex was prepared. Following incubation, the mixture was subject to gel filtration using an S200 Increase 10/300 GL column equilibrated in HBS. Fractions containing the complex were concentrated with a 100 K Amicon Ultra centrifugal unit at 6,000*g* and 4 °C.

Cryo-EM grids were prepared with an FEI Vitrobot Mark IV (Thermo Fisher) at 4 °C and 100% humidity; 3 µl of complex at 0.2 mg ml^−1^ was applied to Au-Flat 1.2/1.3 grids (Protochips) that had been glow-discharged at 15 mA for 60 s. Following incubation for 5 s, grids were blotted for 1–4 s then plunged into liquid ethane.

Grids were imaged using an FEI Titan Krios operating at 300 kV and equipped with a Gatan BioQuantum energy filter (20 eV) and K3 direct electron detector. Data collection was automated using fast acquisition mode in EPU (Thermo Fisher). Images were acquired at a nominal ×58,149 magnification corresponding to a calibrated pixel size of 0.832 Å per pixel (0.416 Å per super-resolution pixel), at a dose rate of 16.32 electrons Å^−^^2 ^s^−1^ and total exposure time of 3 s with 40 frames. This resulted in a total dose of 48.97 electrons Å^−^^2^. Images were acquired using a 100 µm objective aperture with defocus range of −1.0 to −3.0 µm in 0.25 µm increments. Data were collected from three grids in back-to-back sessions, all prepared with the same sample. A total of 13,524 movies were acquired (7,428 from grid 1, 2,720 from grid 2 and 3,376 from grid 3).

### Image processing

Movies were motion corrected and contrast transfer function parameters estimated on-the-fly in SIMPLE 3.0 (ref. ^36^). Datasets from each session were preprocessed individually. Micrographs were first template picked using templates from a previous pilot data collection and picked particles (1,202,046 from grid 1, 1,601,557 from grid 2 and 752,273 from grid 3, yielding a total of 2,555,876 particles) were extracted (box size of 416 pixels) and subject to two-dimensional classification separately. Following exclusion of particles from poorly defined two-dimensional classes, particles from all three sessions were exported to cryoSPARC v.3.3.2. Here, particles were subject to further rounds of two-dimensional classification and particle clean-up, yielding a combined total of 961,077 particles. These were used for ab initio reconstruction into six classes, which showed that the dataset contained three major species: the PfRCR–Cy.003 complex, a complex lacking PfRH5 (PfCyRPA–PfRIPR–Cy.003) and a complex of mostly PfCyRPA and Cy.003 alone.

Before further refinement, micrographs were repicked using the cryoSPARC implementation of TOPAZ^37^, yielding a total of 2,615,684 particles. These were subject to rounds of two-dimensional classification to remove bad particles then combined with the previous SIMPLE-picked particles. Following removal of duplicates, a final particle stack of 1,686,994 unique particles was obtained. These particles were subject to heterogeneous refinement using volumes for complexes PfRCR–Cy.003, PfCyRPA–PfRIPR–Cy.003 and PfCyRPA–Cy.003, plus three decoy volumes. This separated the particles into PfRCR–Cy.003 (523,352 particles), PfCyRPA–PfRIPR–Cy.003 (527,499 particles), PfCyRPA–Cy.003 (413,374 particles), decoy 1 (107,015 particles), decoy 2 (97,538 particles) and decoy 3 (18,216 particles). Further homogeneous, then non-uniform refinement of PfRCR–Cy.003 and PfCyRPA–PfRIPR–Cy.003 particles yielded maps of 3.2 Å for PfRCR–Cy.003 and 3.3 Å for PfCyRPA–PfRIPR–Cy.003. Following Bayesian polishing of particles in RELION 3.1.3 using default settings^38^ and local per-particle contrast transfer function refinement in cryoSPARC, a final non-uniform refinement yielded consensus maps of 3.0 Å for PfRCR–Cy.003 (500,277 particles) and 3.1 Å for PfCyRPA–PfRIPR–Cy.003 (506,797 particles). Anisotropy analysis of these consensus maps using three-dimensional FSC^39^ and Fourier shell occupancy^40^ analyses showed that the consensus PfRCR–Cy.003 map had a sphericity of 0.91 (ref. ^39^) with an anisotropy transition zone^40^ of 2.7–3.5 Å, and the PfCyRPA–PfRIPR–Cy.003 map had a sphericity of 0.89 (ref. ^39^) with an anisotropy transition zone^40^ of 2.7–3.8 Å.

In both maps the region corresponding to PfCyRPA and Cy.003 was better resolved than the rest of the complex, and PfRIPR was more poorly resolved. Therefore, particles for PfRCR–Cy.003 and PfCyRPA–PfRIPR–Cy.003 were downsampled twofold and individually subjected to three-dimensional variability analysis in cryoSPARC^24^ with three orthogonal principal modes. The motion of each complex was visualized in Chimera^41^ by exporting the output of two-dimensional variability analysis as a volume series containing 20 frames. This indicated that both complexes showed some continuous conformational heterogeneity, manifesting as a pivoting of PfRH5 and PfRIPR. This was largest in the third principal mode analysed for PfRCR–Cy.003 which showed that a portion of PfRIPR had become unresolved over the volume series whereas the portion in contact with PfCyRPA remained mostly unchanged.

The density for PfRH5 and PfRIPR in PfRCR–Cy.003 was improved by local refinement. PfRH5 was locally refined using a soft mask around PfRH5–PfCyRPA. Before local refinement of PfRIPR, a more homogeneous subset of particles was obtained using heterogeneous refinement and volume outputs from three-dimensional variability analysis. This subset (253,444 particles) was signal subtracted such that it contained signal for PfRIPR only and was then subject to local refinement, first using a soft mask around PfRIPR then around a subportion of PfRIPR, guided by the motions observed in three-dimensional variability analysis. This yielded a map for PfRIPR with a reported global resolution of 3.3 Å, sphericity^39^ of 0.76 and anisotropy transition zone^40^ of 2.8–3.9 Å. The same procedure was used for PfRIPR in PfCyRPA–PfRIPR–Cy.003 except that the full particle stack (506,797 particles) was used.

Some two-dimensional classes indicated that there was additional density for PfRIPR that was not resolved in these maps. To visualize this extra region, a subset of particles (62,817) was processed by non-uniform refinement using the PfRCR–Cy.003 volume low-pass filtered to 30 Å as a reference. This yielded a map showing a small region of additional density projecting from the middle of the PfRIPR core, with a reported global resolution of 4.0 Å. Refinement with wider masks around this region did not result in more of PfRIPR being resolved.

The local resolution of unsharpened maps was estimated using cryoSPARC. Composite maps for PfRCR–Cy.003 and PfCyRPA–PfRIPR–Cy.003 were generated from consensus and local refinement maps in PHENIX^42^. Maps were postprocessed using the default parameters of DeepEMhancer^43^ to aid model building. All maps were rendered in ChimeraX^44^.

### Model building and refinement

To aid model building of the PfRCR–Cy.003 complex, the crystal structures of PfRH5 (PDB ID: 4U0Q, chain C)^3^, the PfCyRPA–Cy.003 Fab complex (PDB ID: 7PI2, chains D–F)^5^ and an AlphaFold2 (ref. ^28^) (v.2.1.1)-predicted model of PfRIPR (20–716, except residues 484–548) were docked into the PfRCR–Cy.003 composite map as starting models using ChimeraX. These were manually rebuilt with COOT and ISOLDE^45^ in iterative cycles. The C-terminal tail of PfRH5 was built de novo. Regions of PfRIPR (the N terminus, from domain N2 to the end of EGF2, and EGFs 3 and 4) were lower resolution and less interpretable than the remainder of the composite map. The AlphaFold2 prediction of PfRIPR was used to guide building in these areas to place structural elements. To build the PfCyRPA–PfRIPR–Cy.003 complex, the crystal structure of the PfCyRPA–Cy.003 Fab complex (PDB ID: 7PI2, chains D–F)^5^ and PfRIPR from the PfRCR–Cy.003 structure were rigid-body fitted into the PfCyRPA–PfRIPR–Cy.003 composite map as starting models then manually rebuilt using COOT. The region corresponding to PfRIPR in the PfCyRPA–PfRIPR–Cy.003 map was less well resolved than in the PfRCR–Cy.003 counterpart, especially around the periphery of PfRIPR. For this reason, following docking, the PfRIPR model from PfRCR–Cy.003 was largely unedited with no changes made to some regions (for example, the N terminus and around residues 661–667). In each case, models were refined in PHENIX using global minimization and secondary structure restraints against their respective composite maps (which had not been postprocessed).

The model of PfRCR–Cy.003 comprises PfRIPR residues 34–716 (except 124–137 and 479–557), PfCyRPA residues 33–358, PfRH5 residues 159–516 (except 242–303), Cy.003 light-chain residues 23–229 and Cy.003 heavy-chain residues 21–245 (except 156–166). The model of PfCyRPA–PfRIPR–Cy.003 comprises PfRIPR residues 34–716 (except 124–137 and 479–558), PfCyRPA residues 34–358 (except 69–73, 124–127, 245–249 and 319–323), Cy.003 light-chain residues 23–229 and Cy.003 heavy-chain residues 21–245 (except 156–166).

To aid with map interpretation in which density was not continuous for PfRIPR beyond Pro716, an AlphaFold2 model of PfRIPR truncated after EGF5 (residues 20–769) was generated. Docking this prediction into the PfRCR–Cy.003 map suggested that the remaining density probably corresponded to EGF5. In addition, a model of the tail of PfRIPR (residues 717–1,086, comprising EGF5 to its C terminus) was separately predicted. This was manually docked into the second 4.0 Å PfRCR–Cy.003 map, showing additional density for the tail of PfRIPR, to generate a composite model of full-length PfRIPR.

To identify structural homologues of PfRIPR domains, the built structure of the PfRIPR core (residues 34–716) and the AlphaFold2-predicted structure of the PfRIPR tail (residues 717–1,086) were analysed by DALI^46^, searching against the PDB25 database.

### XL-MS of the PfRCR complex

Chemical crosslinking was performed on two separate 100 pmol aliquots (100 μl at 0.2 mg ml^−1^) of PfRCR in 100 mM phosphate buffer pH 7.4; 2 µl of 5 mM DSSO (Thermo Fisher) in 10% DMSO and 100 mM phosphate buffer pH 7.0 was then added for 1 h at room temperature and quenched with 8 µl of 5% (v/v) of hydroxylamine in water (Sigma-Aldrich). Next, 1 μl of 200 mM TCEP was added for 1 h at 55 °C and free thiol groups were alkylated with 1 μl of 380 mM Iodoacetamide (Thermo Fisher) for 30 min at room temperature in the dark. Sequential double digestions were performed with Sequencing Grade Modified Trypsin (Promega) and Sequencing Grade Chymotrypsin (Roche) at an enzyme:substrate weight ratio of 1:25 for 3 h, and then overnight at 37 °C in 100 mM phosphate buffer pH 7.4. Digests were diluted 1:4 with water containing 5 vol% DMSO and 0.1 vol% formic acid before nanoscale liquid chromatography–mass spectrometry (LC–MS) analysis.

Digested protein samples were subjected to nanoscale LC–MS analysis^47^ using a Reprosil-Pur C18-AQ trapping column (20 mm length × 100 µm internal diameter, 5 µm particle size, 200 Å pore size) and a Reprosil-Pur C18-AQ analytical column (30 cm length × 50 µm internal diameter, 3 µm particle size, 125 Å pore size), both packed in-house. A 10 µl sample was loaded onto the trapping column at 3 µl min^−1^ of solvent A (0.1 vol% formic acid in water) for 10 min. The trapping column was then switched in line with the analytical column and gradients applied from 8 to 43% of solvent B (acetonitrile + 0.1 vol% formic acid) at 125 nl min^−1^. The column effluent was subjected to electrospray ionization at a spray tip voltage of 2.1 kV and heated capillary temperature of 200 °C.

Mass spectra were acquired in an Orbitrap Fusion Lumos mass spectrometer (Thermo Fisher Scientific) operated in data-dependent acquisition mode. Full mass spectrometry scans were acquired with an Orbitrap readout (*m*/*z* scan range 350–1,500 Th, mass resolution 120,000 full-width at half-maximum and normalized automatic gain control (AGC) target of 100%). Collision‐induced dissociation (CID) fragmentation spectra (employing 30% CID energy) from multiply charged (2+ to 8+) precursor ions were acquired with an Orbitrap readout at 15,000 full-width at half-maximum mass resolution, with ion injection time limited to 600 ms and a normalized AGC target of 100%. MS3 scans, acquired to identify the peptide partners of DSSO-cross-linked dipeptides, were triggered only following recognition of a 31.9721 Da (±10 ppm) mass difference for a mass spectral doublet in the MS2 scan having a partner intensity range of 10–100% (ref. ^48^). Both doublet partners were subjected to CID fragmentation (employing 35% CID energy) at a maximum injection time of 600 ms, normalized AGC target of 200% and ion trap readout for the MS3 scan.

Proteome Discoverer 2.5 (Thermo Fisher Scientific) containing the XlinkX search node was used to process LC–MS data and identify crosslinked peptides. Dynamic modifications (oxidation of methionine and deamidation of asparagine) and static carbamidomethyl modification of cysteine residues were included in the search. The XlinkX search node was used, with DSSO defined as a mass spectrometry-cleavable crosslinker on lysine, serine, threonine and tyrosine residues^49^. In addition, dead-end dynamic modifications on these residues as hydrolysed or amidated DSSO were included. Mass tolerances for MS1, MS2 and MS3 scans were set to 10 ppm, 50 ppm and 0.5 Da, respectively. The false discovery rate threshold for the XlinkX Validator was set to 1%. Identified crosslinks were visualized with xiNET^50^ to validate and aid building of the PfRCR–Cy.003 cryo-EM structure using a Cα–Cα distance threshold of 35 Å (ref. ^51^), mapped and measured using ChimeraX.

### Microscale thermophoresis

Full-length basigin and BSG^ecto^ were fluorescently labelled with Alexa Fluor 488 using an Alexa Fluor 488 protein-labelling kit (Thermo Fisher) as recommended. Excess dye was removed by gel filtration on an S75 Increase 10/300 column using a buffer containing 20 mM HEPES pH 7.2 and 150 mM NaCl for BSG^ecto^, or with the same buffer also containing 0.02% DDM:cholesteryl hemisuccinate (10:1 w/v ratio) for full-length basigin. To measure the binding of PfRH5 and PfRCR to BSG^ecto^, a twofold dilution series of PfRH5 or PfRCR (concentration range 8 µM to 3.91 nM) was prepared in 20 mM Tris pH 8.0, 200 mM NaCl, 1 mg ml^−1^ salmon sperm DNA and 0.01% Tween-20. Basigin ectodomain was held at constant 0.25 µM throughout the dilution series. To measure the binding of PfRH5 and PfRCR to full-length basigin, a similar twofold dilution series of PfRH5 and PfRCR (concentration range 2 µM to 0.12 nM) was prepared in 20 mM Tris pH 8.0, 200 mM NaCl, 1 mg ml^−1^ salmon sperm DNA and 0.02% DDM. Full-length basigin was held at a constant concentration of 0.1 µM. The sample was incubated for 10 min and centrifuged at 10,000*g* for 10 min, then the supernatant was transferred to Monolith NT.114 series premium capillaries (NanoTemper). Experiments were performed at 25 °C on a Monolith NT.115. Because significant variations in raw fluorescence were observed for both PfRH5 and PfRCR measurements against full-length basigin (at over 2 µM) and basigin ectodomain (at over 5 µM) during data collection, data from these concentrations were excluded from analysis. Binding experiments were performed in triplicate for two separately prepared samples. Data were analysed using software v.1.5.41 (NanoTemper).

### Surface plasmon resonance

PfRIPR and PfRIPR tail were immobilized onto separate flow paths of a CM5 Series S Sensor Chip (Cytiva) using the standard amine-coupling protocol, yielding approximately 8,000 response units for PfRIPR and around 2,000 for PfRIPR tail. The PfCSS–PfPTRAMP heterodimer complex was prepared at either 8.0 or 7.7 µM in SPR buffer (20 mM HEPES pH 7.5, 150 mM NaCl, 0.01% Tween-20), then a twofold serial dilution series was prepared in the same buffer. SPR traces were recorded on a T200 Biacore instrument (Cytiva) in SPR buffer at 25 °C, at a flow rate of 30 µl min^−1^ and either 150 or 180 s injections. Experiments were performed four times with four independent dilution series, and binding affinities estimated by steady-state analysis using T200 Biacore Evaluation Software (Cytiva).

### Design and expression of cysteine-locked PfRH5

The Rosetta Disulfidize^52^ protocol was used to design cysteine locks in PfRH5. Both wild-type and thermally stabilized PfRH5 crystal structures (PBD ID: 6RCO^4^ and 5MI0 (ref. ^53^)) were used for design simulations. Following the introduction of cysteine locks, the designed models were relaxed using the Rosetta FastRelax^54^ protocol. The top-scoring models based on both ‘total_score’ and ‘dslf_fa13’^55^ were manually inspected, and five disulfide cysteine-lock designs connecting the N- and C-terminal halves of PfRH5 were selected for experimental validation. The Rosetta Disulfidize and Rosetta FastRelax scripts used for model design are provided in Source Data. The selected disulfide locks (CC1, CC2, CC3, CC4 or CC5) were introduced into PfRH5ΔNL (a recombinant construct of PfRH5 lacking its N terminus and the α2–α3 internal loop, with the substitution C203Y (of the 7G8 *P. falciparum* strain))^3^, then wild-type PfRH5ΔNL and the cysteine-locked designs were expressed from stable S2 cell lines, as described above for full-length PfRH5. Culture supernatants were harvested after 3–4 days, 0.45 µm filtered and incubated with CaptureSelect C-tagXL resin (Thermo Scientific). Beads were washed using ten column volumes of either PBS or TBS (25 mM Tris pH 7.5, 150 mM NaCl) and bound proteins eluted with five column volumes of C-tag elution buffer (25 mM Tris pH 7.4, 2 M MgCl_2_). Eluted proteins were further purified by size exclusion chromatography using an S75 Increase 10/300 column with a running buffer of PBS or TBS (25 mM Tris pH 7.5, 150 mM NaCl). Following biophysical characterization of cysteine-lock designs CC1–CC5, a final cysteine-locked version of PfRH5 (PfRH5^CL^) was generated through combination of cysteine locks CC1 and CC5. These were introduced into PfRH5ΔNL, then expressed and purified as for other single-cysteine-lock versions.

### Circular dichroism of cysteine-locked PfRH5

Circular dichroism spectra of wild-type PfRH5ΔNL and single-cysteine-lock designs (CC1–CC5) were recorded at 50 μg ml^−1^ in PBS buffer at 200–250 nm wavelength with a temperature ramp increasing by increments of 2 °C from 20 to 90 °C. PfRH5ΔNL and double-cysteine-locked PfRH5ΔNL^CL^ were buffer exchanged into 10 mM sodium phosphate pH 7.5 and 50 mM NaF with Zeba Spin Desalting Columns, followed by circular dichroism spectra recorded at 75 μg ml^−1^ and 190–250 nm with temperature ramp increasing by increments of 2 °C from 20 to 90 °C. A Jasco J-815 Spectropolarimeter was used for all measurements. Data analysis was performed using GraphPad Prism v.8.4.3.

### Maleimide-PEG2-biotin labelling of cysteine-locked PfRH5

PfRH5ΔNL^CL^ (10 μg) was buffer exchanged into denaturing buffer (6 M guanidine hydrochloride, 100 mM sodium acetate pH 5.5) using a 0.5 ml 7 K MWCO Zeba Spin Desalting Column (Thermo Scientific, catalogue no. 89882) with incubation at 37 °C for 30 min. EZ-Link maleimide-PEG2-biotin stock (Thermo Scientific, catalogue no. 21901BID) was prepared at 15 mg ml^−1^ in DMSO and diluted to 3 mg ml^−1^ with 100 mM sodium acetate pH 5.5 immediately before use. Denatured PfRH5ΔNL^CL^ was labelled with a 150× molar excess of maleimide-PEG2-biotin at room temperature for 1 h. Excess maleimide-PEG2-biotin was removed by performing buffer exchange into the denaturing buffer using a 0.5 ml 7 K MWCO Zeba Spin Desalting Column. The above steps were carried out with and without the addition of 5 mM TCEP (Thermo Scientific, catalogue no. 77720) during the denaturation step. The extent of maleimide-PEG2-biotin labelling was then assessed by intact mass analysis using mass spectrometry.

### Intact mass analysis of maleimide-labelled, cysteine-locked PfRH5

Reversed-phase chromatography was performed in-line before mass spectrometry using an Agilent 1100 high-performance liquid chromatography system (Agilent Technologies). Concentrated protein samples were diluted to 0.02 mg ml^−1^ in 0.1% formic acid, and 50 µl was injected onto a 2.1 × 12.5 mm^2^ Zorbax 5 µm 300SB-C3 guard column housed in a column oven set at 40 °C. The solvent system used consisted of 0.1% formic acid in ultrahigh-purity water (Millipore) (solvent A) and 0.1% formic acid in methanol (LC–MS grade, Chromasolve) (solvent B). Chromatography was performed as follows: initial conditions were 90% A and 10% B and a flow rate of 1.0 ml min^−1^. After 15 s at 10% B, a two-stage linear gradient from 10% to 80% B was applied over 45 s and then from 80 to 95% B over 3 s. Elution then proceeded isocratically at 95% B for 72 s followed by equilibration at initial conditions for a further 45 s. Protein intact mass was determined using a 1969 MSD-ToF electrospray ionization orthogonal time-of-flight mass spectrometer (Agilent Technologies). The instrument was configured with the standard electrospray ionization source and operated in positive-ion mode. The ion source was operated with capillary voltage 4,000 V, nebulizer pressure 60 pounds per square inch gauge, drying gas 350 °C and drying gas flow rate 12 l min^−1^. The instrument ion optic voltages were as follows: fragmentor 250 V, skimmer 60 V and octopole RF 250 V.

### Modelling of PfPCRCR–antibody complexes

For visualization of PfRCR in the context of the erythrocyte surface, structure-based alignments were used to dock PfRCR onto membrane-bound basigin using crystal and cryo-EM-derived structures, either determined in this study or previously published. To achieve this, the model of PfRIPR (derived from the PfRIPR core determined here by cryo-EM and AlphaFold2 prediction of the PfRIPR tail, docked onto one another as detailed above) was first aligned onto the PfRCR–Cy.003 complex cryo-EM structure to generate a full-length model of PfRCR. Next, the crystal structure of PfRH5 bound to BSG^ecto^ (PDB ID: 4U0Q)^3^ was aligned onto the PfRH5 component of PfRCR. This basigin–PfRCR–Cy.003 composite model was then aligned onto either MCT1-bound basigin (PDB ID: 7CKR)^33^ or PMCA-bound basigin (PDB ID: 6A69)^34^ using the D2 domain of basigin as a target. To assess the location of neutralizing PfCyRPA epitopes in this erythrocyte-bound context of the PfRCR complex, crystal structures of PfCyRPA-bound Cy.004 (PDB ID: 7PHW)^5^, Cy.007 (PDB ID: 7PHV)^5^, 8A7 (PDB ID: 5TIH)^19^ and c12 (PDB ID: 5EZO)^20^ were aligned onto the PfRCR complex using PfCyRPA as a target. Structural alignments were performed in COOT or ChimeraX.

### Flow cytometry analysis of PfRCR binding to erythrocytes

Erythrocytes (O^+^ or O^−^) were washed twice in PBS containing 1% w/v bovine serum albumin (PBS/BSA), then 50 µl aliquots containing approximately 10 million cells were prepared. PfRCR was prepared by mixing equimolar amounts of PfRH5ΔN^3^, PfCyRPA and PfRIPR in PBS/BSA followed by incubation at room temperature for 30 min. Where the blocking activity of anti-PfRH5 or anti-PfCyRPA monoclonal antibodies was being assayed, these were included at twofold molar excess during this incubation period. Following this, aliquots of erythrocytes were centrifuged at 1,000*g* for 1 min, the supernatant removed and cells resuspended with the prepared protein complexes and incubated for 1 h at room temperature on a roller. Following this incubation period, red blood cells were recovered by spinning as before then washed twice in PBS/BSA. To quantify the binding of PfRCR to erythrocytes, samples were stained in one of two ways as detailed below, then washed three times with PBS/BSA followed by dilution to approximately 6 million cells per ml before analysis with a S3e Cell Sorter operated with ProSort Software v.1.6.0.12 (Bio-Rad). For each sample, binding was performed in a volume of 50 µl and 50,000 events were recorded. Data were analysed using FlowJo v.10.9 (Becton Dickinson). Erythrocytes were gated by plotting forward-scatter area against side-scatter area, then singlets were identified by plotting forward-scatter area against forward-scatter height. Positively labelled erythrocytes corresponding to those with bound PfRCR were identified by plotting forward-scatter area against Alexa Fluor 488 area, with positive-gate placement guided by unstained red blood cells and those incubated with detection antibodies/nanobodies only (Supplementary Fig. 2). The number of positive cells is expressed as a percentage of the number of singlets recorded. Statistical analyses were performed using GraphPad Prism v.9.2.0.

To verify that the non-neutralizing anti-PfRH5 antibody R5.011 (ref. ^4^) could be used to quantify PfRCR binding to erythrocytes without reducing PfRCR binding, we first studied PfRCR that had been labelled using the CaptureSelect Alexa Fluor 488 Anti-C-tag Conjugate (no. 7213252100, Thermo Scientific). Following incubation of erythrocytes with 2 µM PfRCR, alone or in the presence of 4 µM anti-PfRH5 antibodies R5.004 or R5.011 (ref. ^4^), cells were washed and incubated with CaptureSelect Alexa Fluor 488 Anti-C-tag Conjugate for 1 h at room temperature in the dark. Erythrocyte binding was then quantified as above, measured in duplicate.

To assay the ability of anti-PfCyRPA antibodies to block PfRCR binding to erythrocytes, these were incubated with either 400 nM PfRCR alone or in the presence of 800 nM anti-PfCyRPA antibodies Cy.003, Cy.004 or Cy.007 (ref. ^5^). The anti-PfRH5 antibody R5.004, which blocks PfRH5 binding to basigin^4^, was used as a positive control. Following incubation with protein complexes, erythrocytes were washed and stained by incubation with the monoclonal anti-PfRH5 antibody R5.011 as a primary antibody, followed by washing once then incubation with goat anti-human IgG cross-adsorbed Alexa Fluor 488 secondary antibody (no. A11013, Invitrogen). Primary and secondary antibodies were each used at 10 µg ml^−1^ in PBS/BSA for 1 h at room temperature in the dark. Erythrocyte binding was then quantified as above, measured in triplicate. Because Cy.004 binds to PfCyRPA in a calcium-dependent manner^5^, cells were supplemented with 1 mM CaCl_2_ during protein incubation with this antibody. As a control, PfRCR alone was incubated with red blood cells both in the presence and absence of 1 mM CaCl_2_, with no significant change in the number of positive cells observed.

### Haemolysis assay

Red blood cells (100 µl) were washed twice by dilution in 10 ml of PBS followed by spinning at 500*g* for 5 min, then diluted to approximately 4 million cells per ml in PBS. Next, 200 µl of diluted cells was aliquoted into wells of a 96-well plate then spun once more at 500*g* for 5 min and the supernatant discarded. Cells were then resuspended in either 200 µl of PBS containing 2 µM PfRH5, 2 µM PfCyRPA, 2 µM PfRIPR and 2 µM PfRCR or 2 µM alpha-haemolysin (Sigma) for 24 h at 37 °C. In addition, PBS containing 1% v/v Triton X-100 or PBS alone was used as positive and negative control, respectively. Following incubation, cells were spun at 500*g* for 5 min then 50 µl of each solution transferred to a new 96-well plate. Absorbance at 405 nm was used to assess the degree of haemolysis using a microplate reader (Tecan). For each protein sample group, background signal observed with PBS alone was subtracted then haemolysis reported relative to complete cell lysis, as in the positive control (1% Triton X-100). Data were collected four times independently for all samples except for PfRIPR, which was collected in duplicate. Statistical analyses were performed using GraphPad Prism v.9.2.0.

### Calcium flux assay

Erythrocytes (O^+^) were loaded with Fluo-4 through their incubation with 6 µM Fluo-4AM in RPMI (supplemented with 0.5% w/v AlbuMAX (Gibco) and 4 g l^−1^ glucose) for 1 h at 37 °C. Following washing and resting of these Fluo-4-loaded erythrocytes for 15 min, recombinant protein (PfRH5, PfRH5ΔN or PfRCR) was added to the cells at 2 µM in RPMI then fluorescence (excitation 488 nm, emission 535 nm) was measured over 800 s in black-bottomed, 384-well plates in a CLARIOstar microplate reader with software v.5.6.0 R2 (BMG Labtech). RPMI alone was used as a negative control. Measurements were recorded in technical triplicate. To verify the loading of Fluo-4 into erythrocytes, lysis was induced by the addition of 0.1% v/v Triton X-100 in RPMI and fluorescence measured as above. Untreated erythrocytes were used as a control.

### *P. falciparum* culture and transfection

Blood-stage *P. falciparum* parasites were cultured in human erythrocytes (UK NHS Blood and Transplant) at 3% haematocrit with custom RPMI-1640 medium supplemented with 2 mM l-glutamine, according to previously established methods^56^. All parasites used in this study were derived from *P. falciparum* line *p230p*DiCre, generated in strain 3D7 (ref. ^44^). Parasites were synchronized by purification of schizont stages using a Percoll gradient, facilitating reinvasion, followed by sorbitol treatment of newly formed ring stages.

Transfections were performed as previously described^57^. Transgenic parasites were further cloned by limiting dilution. For induction of dimerizable, Cre-mediated excision of floxed DNA, early-ring-stage parasites were treated with 10 nM rapamycin or DMSO as control. Parasite samples for PCR and immunoblot analysis were collected around 40–42 h following rapamycin treatment.

### Generation and genotyping of transgenic *P. falciparum* parasites

CRISPR–Cas9 guide RNA sequences targeting either the 5' region (guide 1: 5'GCTATATAAACATATTTACG-3' and guide 2: 5'-TTTGAATTTACTATATGTAC-3') or the 3' region of the *PfRh5* open reading frame (5'-TTGTCATTTCATTGTGTAAG-3') were identified using EuPaGDT (http://grna.ctegd.uga.edu/). Each guide was cloned into vector pDC2-Cas9-hDHFRyFCU^57^, generating plasmids pDC2-Cas9-5'guide1, pDC2-Cas9-5'guide2 and pDC2-Cas9-3'guide. All primers used to generate DNA repair plasmids, along with templates and expected PCR product sizes, are listed in Supplementary Tables 1 and 2.

A DNA repair template designed to replace the endogenous *PfRh5* intron with a synthetic *SERA2 LoxP* intron (*LoxPint*)^27^ was synthesized with 300–350-base-pair homology regions spanning the *PfRh5* 5′ untranslated region (UTR) and coding regions on either side of the endogenous intron. Homology region 1 (amplified with primers 1 and 2) started at 5'-GGTAAATGTAGGATTGTTCT-3' and ended at 5'-ATAATGGTCAAAATTAATTT-3', and homology region 2 (amplified with primers 3 and 4) spanned 5'-GATTAAGTTTTGAAAATGCA-3' to 5'-ATCCACATTTTTATAGTCTT-3'. Both homology arms, and the intervening *SERA2 LoxPint* module (amplified with primers 5/6), were stitched together using overlapping extension PCR (using primers 1 and 6, followed by primers 1 and 4). The final PCR product was inserted into pGEM-T Easy (Promega), linearized using NcoI/SpeI and mixed with plasmid pDC2-Cas9-5' guide1 and pDC2-Cas9-5' guide2 before transfection, generating line PfRH5^NT^.

A DNA repair template for insertion of a *LoxP* sequence plus half of the *SERA2* intron (5'-ATAACTTCGTATAGCATACATTATACGAAGTTATTATATATGTATATATATATATATTTATATATTTTATATTCTTTTAG (*LoxP* sequence underlined)) directly after the *PfRh5* stop codon was synthesized with a 300–350-base-pair homology region spanning the coding and 3' UTR regions on either side of the *PfRh5* C-term Cas9 cut site. Homology region 1 (amplified with primers 10 and 11) started at 5'-GAATTGAATATCATACAAAA and ended at 5'-GTAAGTGGTTTATTTTTTTT. Homology region 2 (amplified with primers 12 and 13) started at 5'-AATGACAAAACATGGTATGT and ended at 5'-CAAGTACGAGCATCCGGAAC. Part of the *LoxPint* module was amplified with primers 14 and 6. All three PCR products were subsequently fused using overlapping extension PCR with primers 10 and 6 followed by 10 and 13. The final PCR product was inserted into pGEM-T Easy (Promega), generating plasmid p*PfRh5*_C-term_*LoxP*. Finally this plasmid was linearized with EcoRI, mixed with pDC2-Cas9-3'guide and transfected into line PfRH5^NT^, generating line PfRH5^cKO^.

For generation of a parasite line containing an inducible mutant *PfRh5* gene, a second copy of *PfRh5* was designed for integration 3' to the floxed, endogenous *PfRh5* copy. Following rapamycin-induced excision of the floxed endogenous locus, the downstream *Rh5* copy would be expressed. For this, a recodonized *PfRh5* sequence was synthesized (GeneArt) starting 3' to the endogenous intron. This recodonized sequence was then flanked by a 5' homologue region (spanning the last 546 base pairs of the endogenous PfRh5 sequence and half of the *LoxPint* module described above) and a 3' homologue region spanning 595 base pairs of the *PfRh5* 3' UTR. The 5' homologue region was synthesized by overlapping extension PCR (using primers 21 and 22, 10 and 23, followed by 21 and 23) and inserted into the GeneArt-generated plasmid using SacII/AflII (homologue region 1 started at 5'-CTTTCATGTTACAATAATAA and ended at 5'-GTAAGTGGTTTATTTTTTTT). The 3' homologue region was also assembled by overlapping extension PCR (using primers 13, 18, 19 and 20 followed by 18 and 20), starting at 5'-AATGACAAAACATGGTATGT and ending at 5'-TGATATAAATGAAGCGTTGA. The final PCR product was inserted into the plasmid via MfeI/SalI, generating the final plasmid, p*PfRh5*_WT_sec_copy. Finally the plasmid was digested using NcoI/NotI, mixed with pDC2-Cas9-3'guide and transfected into the transgenic PfRH5^NT^ parasite line, generating line PfRH5^WT^.

For generation of a parasite line expressing a locking-cysteines *PfRh5* mutant on rapamycin-induced excision of the endogenous floxed *PfRh5 gene*, plasmid p*PfRh5*_WT_sec_copy was modified to introduce the following mutations: L164C, E239C, M478C and H489C. Mutations L164C and E239C were introduced with primers via overlapping extension PCR (using primers 25 and 26, 27 and 28, followed by 25 and 29) using p*PfRh5*_WT_sec_copy as template. The final PCR product was cloned into plasmid p*PfRh5*_WT_sec_copy using AflII/NdeI, yielding plasmid p*PfRh5*_lockingCyst_sec_copy_A. Likewise, mutations M478C and H489C were also introduced by PCR amplification (using primers 30 and 31, 32 and 33, then 30 and 33), followed by cloning of this DNA fragment into plasmid p*PfRh5*_lockingCyst_sec_copy_A using BamHI/MfeI restriction enzymes, yielding plasmid p*PfRh5*_lockingCyst_sec_copy_B. This plasmid was digested using NcoI/NotI, mixed with pDC2-Cas9-3'guide and transfected into the transgenic PfRH5^NT^ parasite line, generating line PfRH5^CL^.

All plasmid DNA sequences were verified by Sanger sequencing. Positions of diagnostic primers used to genotype transgenic parasites are shown in schematics in Extended Data Fig. 6a,b. Diagnostic primer sequences along with expected PCR product sizes are listed in Supplementary Tables 1 and 2. A positive-control PCR reaction using primers 36 and 37 to amplify a 737-base-pair product from the *PfRON2* locus was also included in each set of diagnostic PCRs.

The Qiagen DNeasy Blood and Tissue kit was used for all genomic DNA extractions. All diagnostic PCR analyses were performed using GoTaq Green (Promega) under the following conditions: 5 min at 95 °C, 33 cycles of 30 s at 95 °C, 30 s at 55 °C then 1 min at 30 s kb^−1^ and 64 °C. For amplification of fragments for construct synthesis, CloneAmp HiFi polymerase (Takara) was used. A typical reaction was run with 32 cycles of 5 s at 98 °C, 15 s at 55 °C and 10 s kb^−1^ at 68 °C.

### Immunoblotting

Synchronized schizonts were harvested by Percoll gradient centrifugation, washed in RPMI-1640 without AlbuMax and lysed in SDS sample buffer containing 100 mM dithiothreitol before protein separation on precast Bis-Tris polyacrylamide gels (MPAGE, Merck) and transfer to nitrocellulose membranes by electroblotting. Blots were blocked overnight in 5% milk in PBS with 0.2% Tween-20 and subsequently incubated with either rat anti-PfHSP70 (1:1,000)^58^ or rabbit anti-PfRh5 (1:5,000)^59^, followed by either goat anti-rat horseradish peroxidase (HRP, Sigma) or goat anti-rabbit HRP (Bio-Rad). Detection using enhanced chemiluminescence was carried out using Immobilon Western Chemiluminescent HRP Substrate (Millipore).

### Parasite growth assay

To determine the growth rate of mutant parasites relative to wild-type parasites, ring-stage DMSO and rapamycin-treated cultures were adjusted to a parasitaemia of roughly 0.8% and 2% haematocrit and grown in a gassed chamber at 37 °C. A starting parasitaemia was taken when parasites reached the schizont stage of the same cycle (cycle 0), and again after a further 40 h or so with parasites at schizont stage in cycle 1. Parasitaemia was measured following fixation of cells with 4% paraformaldehyde and 0.1% glutaraldehyde (Sigma) in PBS for 1 h at room temperature, followed by incubation for 1 h at 37 °C with SYBR Green I (Life Technologies).

For flow cytometry analysis, a LSR Fortessa X-20 with BD FACSDiva Software v.9.0 was used with a 530/30 filter, counting 30,000 singlet events per sample. Gating for erythrocytes was achieved by plots of forward-scatter area against side-scatter area (gate P1). Doublet discrimination required gating of forward-scatter area against forward-scatter width (gate P2), followed by side-scatter area against side-scatter width (gate P3). A SYBR Green-stained, uninfected erythrocyte sample was used as a negative control. Gating of SYBR Green-infected erythrocytes was achieved by plotting side-scatter area against Alexa Fluor 488 area using the 530/30 standard filter (gate P4). Parasitaemia was determined by the number of cells identified in gate P4 as a percentage of those in gate P3 (Supplementary Fig. 3). Data were analysed using FlowJo v.10. GraphPad Prism v.9.0.0 was used for statistical analysis (two-tailed, unpaired *t*-test) and graph generation.

### Live-cell imaging of calcium fluxes

For calcium flux assays, erythrocytes were first incubated with 5 μM Fluo-4AM (Invitrogen) in IMDM medium containing 2.5 mM CaCl_2_ and 5 mM Na-pyruvate for 1 h. Cells were subsequently washed three times and then allowed to rest at 37 °C for 30 min. Purified schizonts were added to labelled erythrocytes in a 10–15% parasitaemia and 3% haematocrit culture before dilution to 0.3% haematocrit in the same medium. Cultures were loaded into poly-l-lysine-coated μ-Slide VI 0.4 (Ibidi) channel slides and transferred to a Nikon Ti E inverted-microscope chamber prewarmed to 37 °C. Samples were imaged using a ×60 oil-immersion objective and an ORCA Flash 4.0 CMOS camera (Hamamatsu), at a rate of one frame per second. Videos were acquired and processed using the NIS Advanced Research software package. A total of 41 invasion events were recorded for both PfRH5^WT^ and PfRH5^CL^ parasites, using parasites derived from three biological replicates. All statistical analysis was performed using Prism v.9.0.

### Reporting summary

Further information on research design is available in the Nature Portfolio Reporting Summary linked to this article.

## Online content

Any methods, additional references, Nature Portfolio reporting summaries, source data, extended data, supplementary information, acknowledgements, peer review information; details of author contributions and competing interests; and statements of data and code availability are available at 10.1038/s41586-023-06856-1.

### Supplementary information

Supplementary Information Supplementary Figs. 1–3 and Tables 1–4.

Reporting Summary

Peer Review File

Supplementary Video 1 Three-dimensional variability analysis of the PfRCR complex.

Supplementary Video 2 Wild-type *P. falciparum* merozoites invading erythrocytes preloaded with Fluo-4AM. Video imaged at one frame per second; scale bar, 5 μm; red arrows indicate invasion events.

Supplementary Video 3 Cysteine-locked PfRH5 *P. falciparum* merozoites invading erythrocytes preloaded with Fluo-4AM. Video imaged at one frame per second; scale bar, 5 μm; red arrows indicate invasion events.

### Source data

Source Data Figs. 2–4 and Extended Data Figs. 1, 4, 5, 7 and 8

## Figures and Tables

**Extended Data Table 1:**
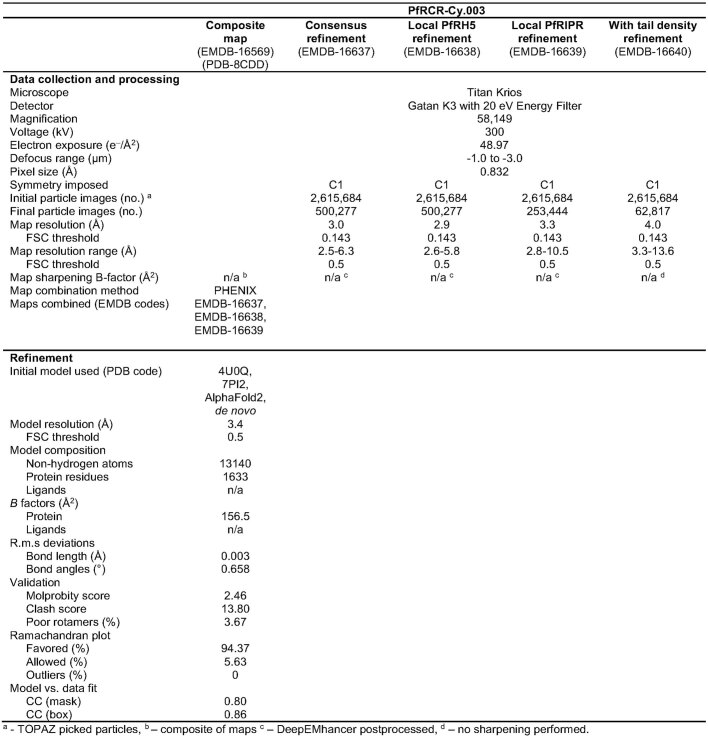
Cryo-EM data collection, refinement, and validation statistics of PfRCR-Cy.003

**Extended Data Table 2:**
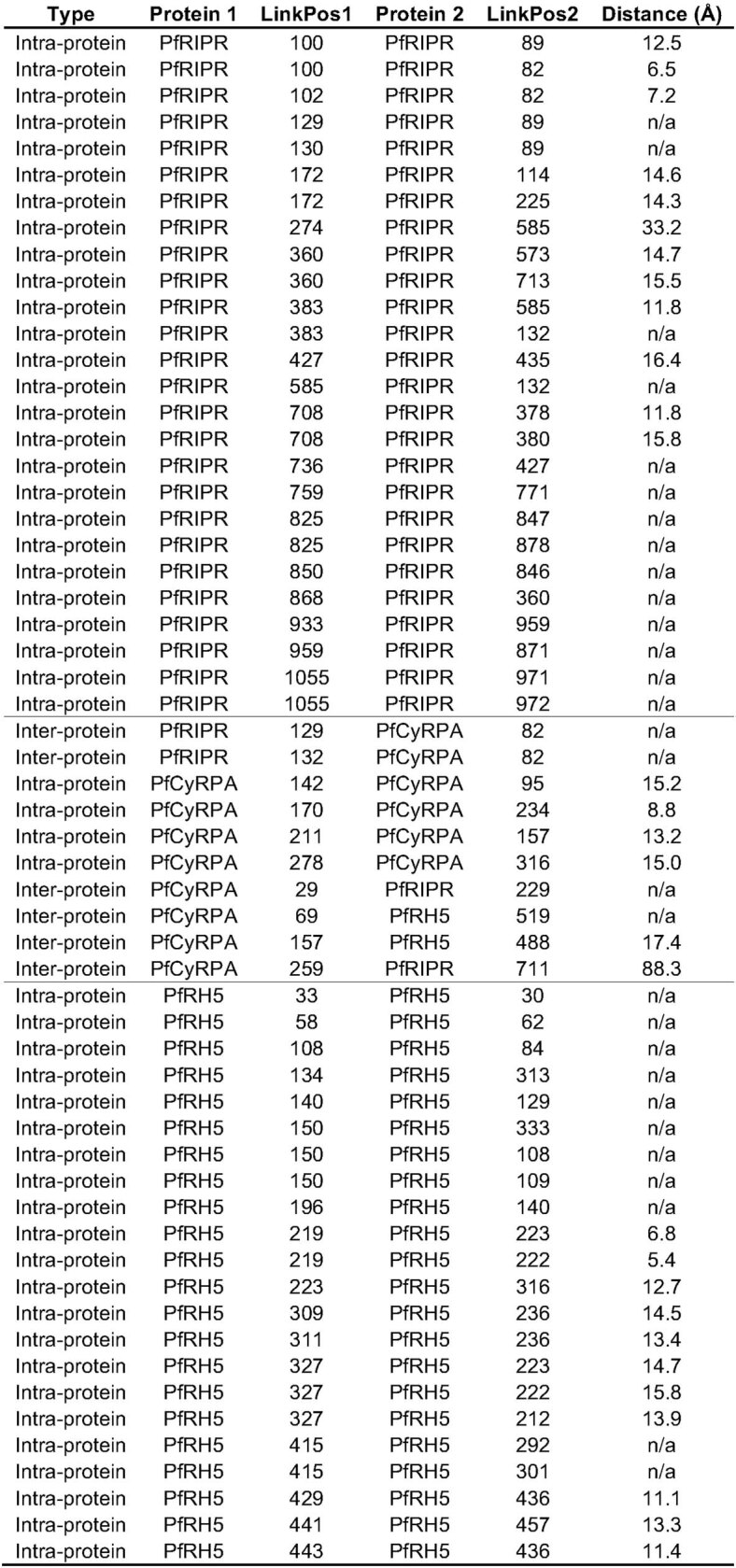
PfRCR mass spectrometry crosslinks

Crosslinks found between PfRIPR, PfCyRPA and PfRH5 after incubating the PfRCR complex with the crosslinker DSSO. LinkPos1 and LinkPos2 refer to the residues crosslinked from each protein. Where possible, the Cα-Cα distance measured between residues in the PfRCR structure is given.

**Extended Data Table 3:**
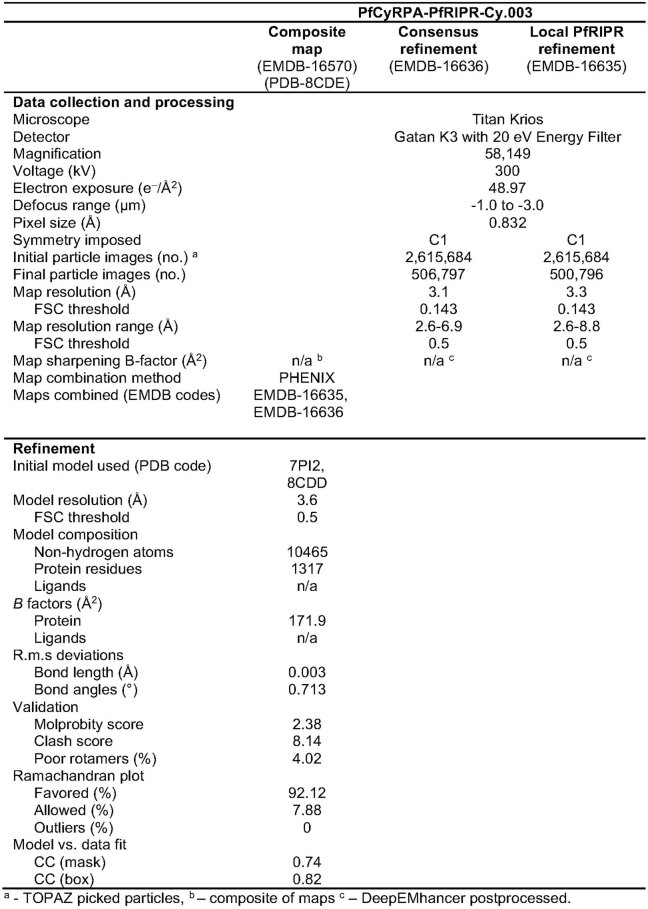
Cryo-EM data collection, refinement, and validation statistics for PfCyRPA-PfRIPR-Cy.003

**Extended Data Table 4:**
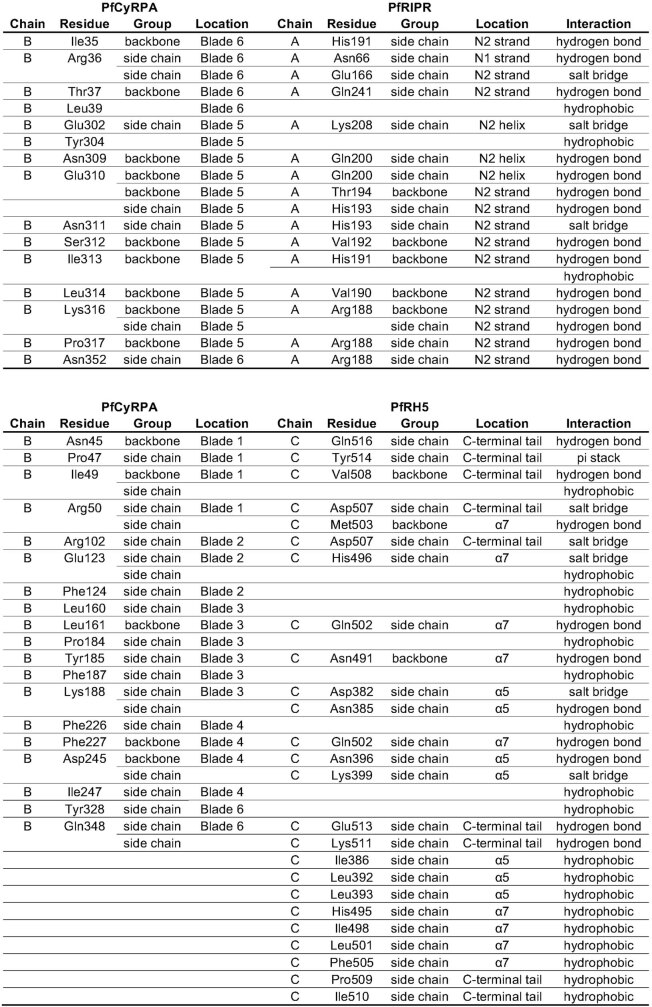
Interface residues of the PfRCR complex

## Data Availability

Cryo-EM maps for PfRCR–Cy.003 are available from the Electron Microscopy Data Bank under accession codes EMDB-16569, EMDB-16637, EMDB-16638, EMDB-16639 and EMDB-16640, and its coordinates from the Protein Data Bank under accession code 8CDD. Cryo-EM maps for PfCyRPA–PfRIPR–Cy.003 are available under accession codes EMDB-16570, EMDB-16636 and EMDB-16635, and its coordinates from the Protein Data Bank under accession code 8CDE. In this study, previously published structures have been used for structural analysis; these can be found in the Protein Data Bank under accession codes 1IGT, 4HL0, 4U0Q, 4U0R, 4U1G, 5EZO, 5FTT, 5TIH, 5TIK, 6A69, 6RCU, 6RCV, 6Z2L, 7CKR, 7PHU, 7PHV, 7PHW, 7PI2 and 7UNY. Uncropped gels and source data for all graphs generated in this study are provided in Supplementary figures and Source data, respectively. The Rosetta Disulfidize and Rosetta FastRelax scripts used to design the cysteine-locked version of PfRH5 in this manuscript are also provided in Source data. All other data are available from the authors on request.

## Code availability

Rosetta scripts used in this study and coordinates for composite molecular models are provided in Source data.
