# Supplementary material for: The PfRCR complex bridges malaria parasite and erythrocyte during invasion
Source: Nature. 2023 Dec 20;625(7995):578–84. doi: 10.1038/s41586-023-06856-1 (PMC10794152; doi:10.1038/s41586-023-06856-1)
Supplement: Supplementary file 1 — Supplementary Figs. 1–3 and Tables 1–4. [file 41586_2023_6856_MOESM1_ESM.pdf]

nature portfolio

https://doi.org/10.1038/s41586-023-06856-1

Supplementary information

The PfRCR complex bridges malaria parasite and erythrocyte during invasion

In the format provided by the authors and unedited

Supplementary Figure 1: Uncropped gels.

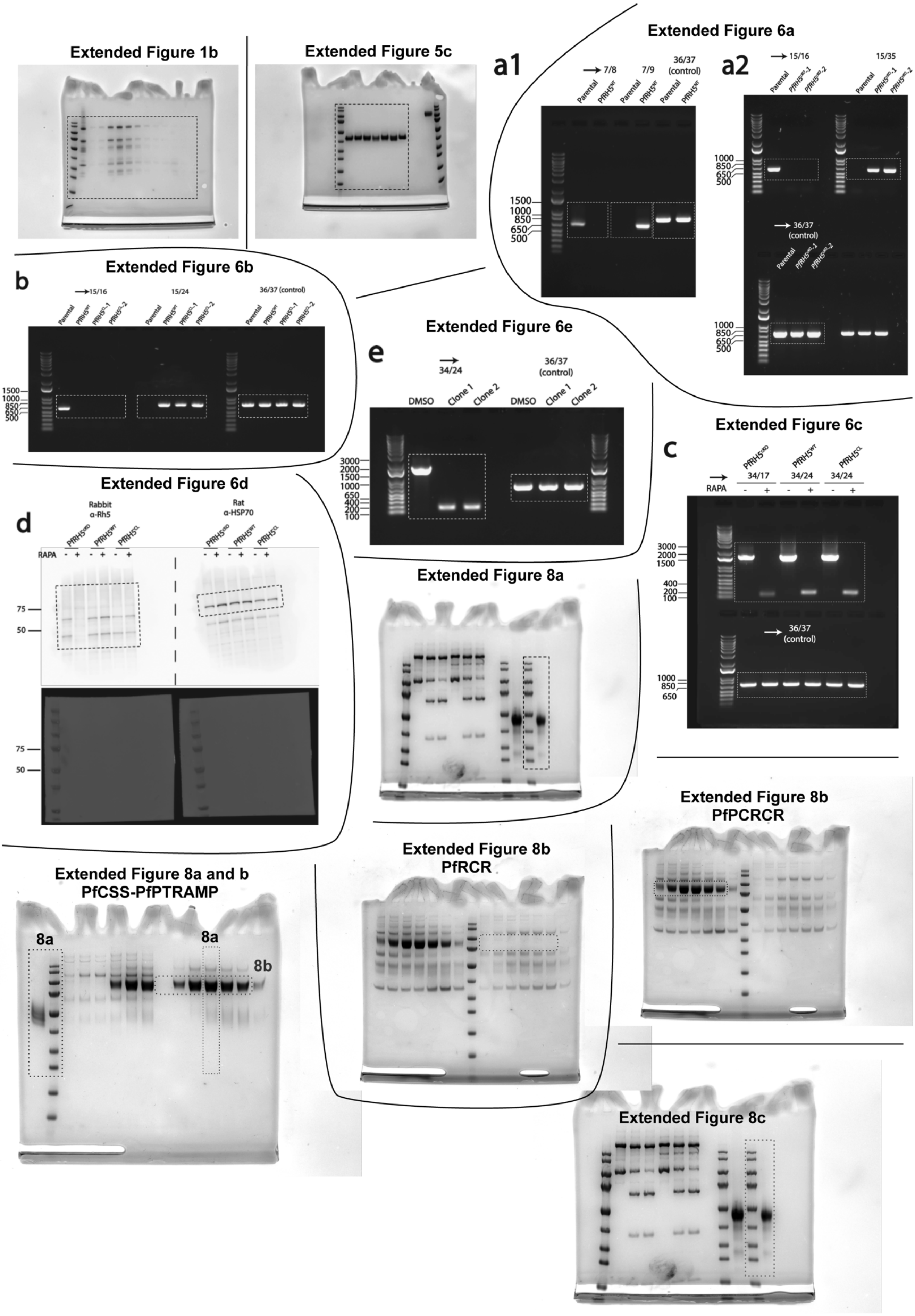

Supplementary Figure 2: FACS gating for PfRCR erythrocyte binding assay.

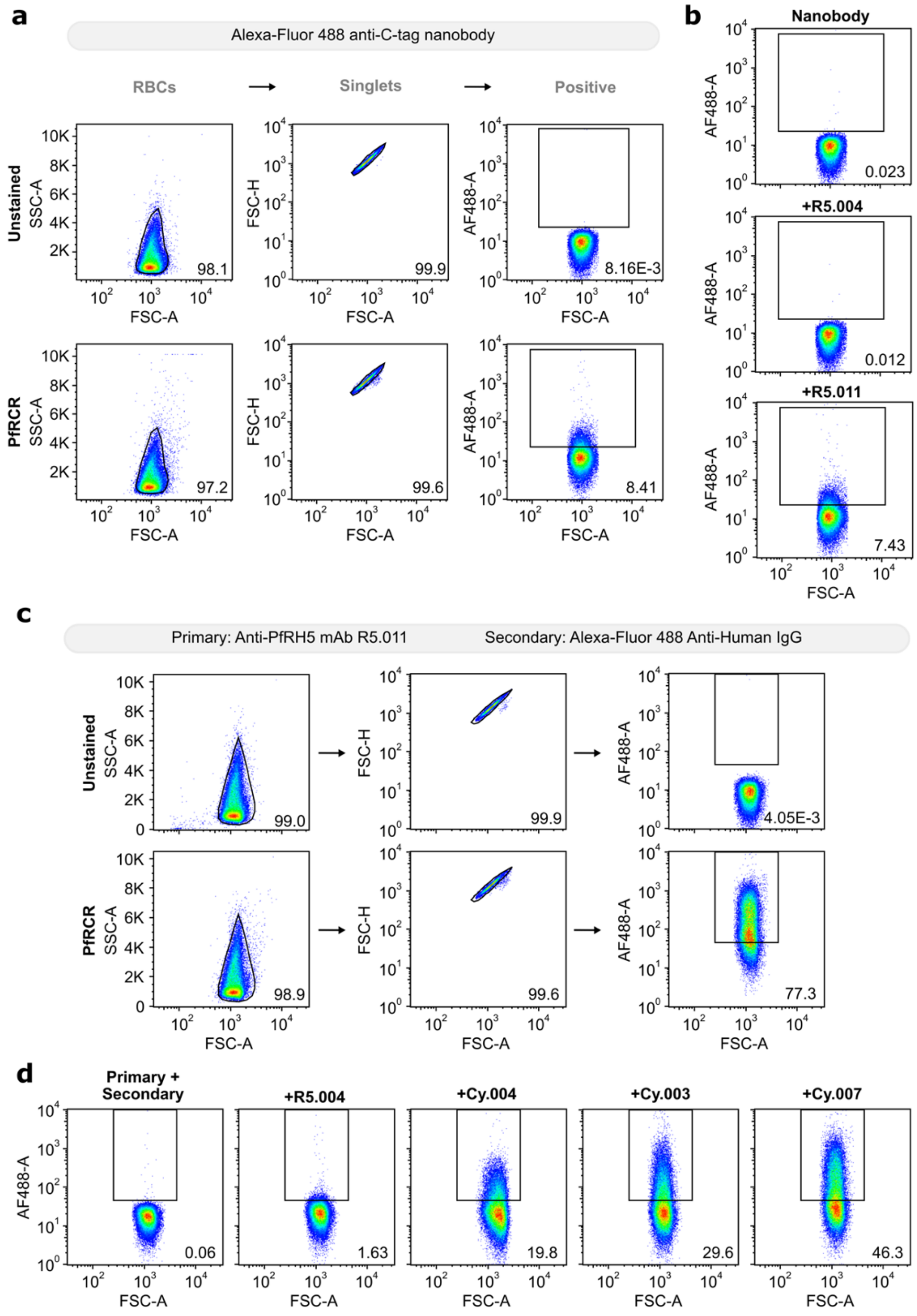

a, Representative scatter plots for the PfRCR erythrocyte binding assay by flow cytometry showing the gating strategy when using the Alexa-Fluor 488 anti-C-tag detection method. Plots for unstained cells and those incubated with PfRCR are shown. Red blood cells (RBCs) were gated using forward scatter area (FSC-A) vs side scatter area (SSC-A), then singlets gated using FSC-A vs forward scatter height (FSC-H). Finally, PfRCR positive cells were gated by plotting FSC-A vs Alexa-Fluor 488 area (AF488-A) and using the gate shown. **b**, Representative scatter plots showing gating of PfRCR positive cells for erythrocytes incubated with anti-C-tag nanobody alone, PfRCR + R5.004, and PfRCR + R5.011. The same gating strategy was used as in (a). The gating strategy in (a) and (b) were used for data presented in Extended Data Figure 8e. **c**, Representative scatter plots for PfRCR erythrocyte binding showing the gating strategy used when using the R5.011 then Alexa-Fluor 488 anti-human IgG detection method. Plots for unstained cells and those incubated with PfRCR are shown. Gating was performed as in (a) using the gates shown. **d**, Representative scatter plots showing gating of PfRCR positive erythrocytes following incubation with primary and secondary antibodies only, with PfRCR + R5.004, PfRCR + Cy.004, PfRCR + Cy.003, and PfRCR + Cy.007. In all plots, the number of gated cells in each plot is shown as a percentage in the bottom right corner of each plot. This gating strategy was used for data presented in Figure 4d.

Supplementary Figure 3: FACS gating used to analyse the proportion of SYBR-positive, parasite infected erythrocytes vs uninfected erythrocytes within a given sample.

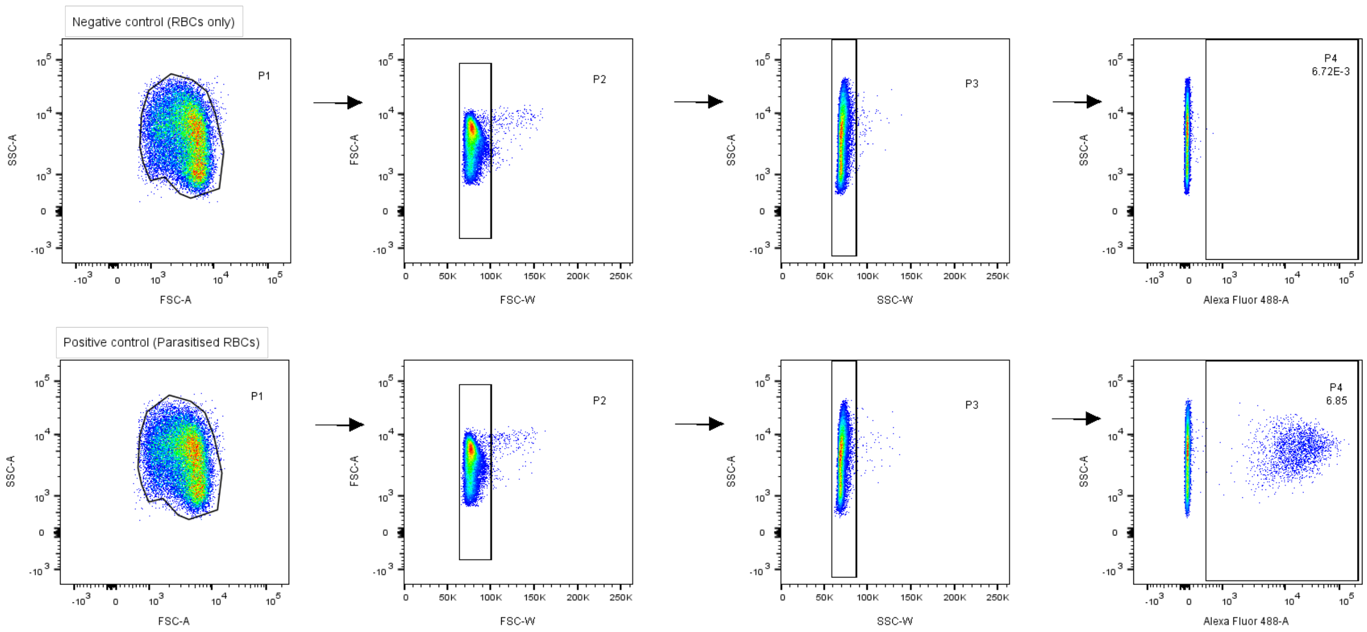

Top panels indicate an erythrocyte (RBC) only, negative control. Bottom panels depict a representative sample containing parasitised erythrocytes. For each set of panels, the percentage of SYBR positive cells is indicated under gate P4. Staining and gating strategies are described in full in the materials and methods section. This gating strategy was used for data presented in Figure 2g.

Supplementary Table 1: Primer numbers and sequences used in this study.

Primer name	Primer sequence
1	ACAATGGTAAATGTAGGATTGTTCTACATAAATG
2	TTCAGGATGAATAAGTGGATGTAGATAATGGTCAAAATTAATTTTTTTTTTATTCTTATCATTCTATTATGTGAC
3	TGTATATATATATATATTTATATATTTTATATTCTTTTAGGATTAAGTTTTGAAAATGCAATAAAAAAAACGAAG
4	ATCCACATTTTTATAGTCTTCATTATTTTTCACATC
5	CTACATCCACTTATTCATCCTGAACCGTAAATAAAAAAAATAATATACAATAACTTCGTATAGCATACATTATAC
6	CTAAAAGAATATAAAATATATAAATATATATATATATACATATATAATAACTTCGTATAATGTATGC
7	TCAAAAATATATACACACATGCATATTACGGTG
8	GTCATATGACCTCGTAAATATGTTTATATAGCACG
9	ACGGTTCAGGATGAATAAGTGGATG
10	AACAGAATTGAATATCATACAAAAATAATAAACGATAAAAC
11	AAGTTATCTACTGGGTAAGTGGTTTATTTTTTTTATATGTTTGAAAATATTCCATTTTAATTG
12	TATATATATATATTTATATATTTTATATTCTTTTAGAATGACAAAACATGGTATGTATATGA
13	GATACAAGTACGAGCATCCGGAAC
14	AACCACTTACCCAGTAGATAACTTCGTATAGCATACATTATACGAAGTTATTATATATG
15	AATATGTATGGATATGAAAAATTATGGTACAAACC
16	TCATATACATACCATGTTTTGTCATTTCATTGTG
17	CTTCGTATAATGTATGCTATACGAAGTTATCTACTGG
18	TTTATATTCTTTTAGAATGACAAAACAATTGGTATGTATATGA
19	TCCGGATGCTCGTACTTGTATCTTACC
20	TCGACCTGCAGGCGGCCGCGAATTCTGATATAAATGAAGCGTTGAATCTTTTATCGG
21	CCGCATCCGCGGCCATGGCTTTCATGTTACAATAATAATTTCTG
22	TTTAGTTTTATCGTTTATTATTTTTGTATGATATTCAATTCTG
23	TCGAAGCTTAAGCCTAAAAGAATATAAAATATATAAATATATATATATATACATATATAATAACTTCGTATAATGTATGC
24	AAGGGTCAAGTTGTTCTCCTGG
25	CTTTTAGGCTTAAGCTTCGAGAACG
26	CCTCCTTCTCCTG**ACA**AATGTCTATTGAGTTCGCTATATTGTAGTTACTC
27	ACATT**TGT**CAGGAGAAGGAGGGTCACTTAGAC
28	TAATGTCATATGGGTG**ACA**AAGCTTCTTAATTGTAGCTATAAGGTCG
29	TAATGTCATATGGGTGACAAAGCTTCTTAATTG
30	CAAGGATTTAAGTGACATGACTAACATATTGC
31	TCCTTACTGTAAAAAGTGTTGTAAAG**ACA**CTGCCTAAGGTGG
32	**TGT**CTTTACAACACTTTTTACAGTAAGGAGAAG**TGT**CTTAACAACATTTTCC
33	TACATACCAATTGTCATTGAGTTAAAGGCTTG
34	CTACATCCACTTATTCATCCTGAACC
35	TCATATACATACCATGTTTTGTCATTCTAAAAG
36	GCAATGAAATATGGTGTATCATTAACATCTGC
37	ATTCTTATTTTCGCGTATGATATGTAATGGTAGC

Supplementary Table 2: PCR reactions used for synthesizing homologous repair donor DNA plasmids and genotyping transgenic parasites.

PCR Product/target locus	Fwd primer	Rev primer	Product size (bp)	Template(s)
Generation of *PfRh5* N-term *LoxP* donor DNA plasmid (white)/ genotyping of transgenic line (green)				
HR1	1	2	343	Pf 3D7 gDNA
HR2	3	4	390	Pf 3D7 gDNA
LoxPint	5	6	129	SERA2 LoxPint
HR1-LoxPint fusion	1	6	448	HR1 + LoxPint PCR products
Full length insert	1	4	798	HR1-LoxPint + HR2 PCR products
'WT' locus	7	8	675	gDNA from transfection
Integrated locus	7	9	585	gDNA from transfection
Generation of *PfRh5* C-term LoxP donor DNA plasmid (white)/ genotyping of transgenic line (green)				
HR1	10	11	325	Pf 3D7 gDNA
HR2	12	13	390	Pf 3D7 gDNA
LoxPint	14	6	97	SERA2 LoxPint
HR1-LoxPint fusion	10	6	398	HR1 + LoxPint PCR products
Full length insert	10	13	752	HR1-LoxPint + HR2 PCR products
'WT' locus	15	16	627	gDNA from transfection
Integrated locus	15	17	631	gDNA from transfection
Generation of *PfRh5* WT second copy donor DNA plasmid (white)/ genotyping of transgenic line (green)				
HR2 fragment 1	18	13	371	PfRh5 C-term LoxP plasmid
HR2 fragment 2	19	20	296	Pf 3D7 gDNA
HR2 full length	18	20	645	HR2.1 + HR2.2 PCR products
HR1 fragment 1	21	22	300	PfRh5 C-term LoxP plasmid
HR1 fragment 2	10	23	411	Pf 3D7 gDNA
HR1 full length	21	23	666	HR1.1 + HR1.2 PCR products
WT locus	15	16	627	gDNA from transfection
Integrated locus	15	24	740	gDNA from transfection
Generation of *PfRh5* locking cysteines second copy donor DNA plasmid (white)/ genotyping of transgenic line (green)				
Fragment 1.1	25	26	448	PfRh5 WT second copy plasmid
Fragment 1.2	27	28	249	PfRh5 WT second copy plasmid
Fragment 1 full length	25	29	676	Fragments 1.1 + 1.2
Fragment 2.1	30	31	330	PfRh5 WT second copy plasmid
Fragment 2.2	32	33	163	PfRh5 WT second copy plasmid
Fragment 2 full length	30	33	464	Fragments 2.1 + 2.2
WT locus	15	16	627	gDNA from transfection
Integrated locus	15	24	740	gDNA from transfection
Diagnostic PCRs to detect rapamycin induced excision of floxed *PfRh5*				
PfRh5 cKO excised/non excised locus	34	35	1752 if non-excised; 155 if excised	gDNA from Rap/DMSO treated parasites
PfRh5 cKO + WT second copy excised/non excised locus	34	24	1785 if non-excised; 188 if excised	gDNA from Rap/DMSO treated parasites
PfRh5 cKO + locking cysteines second copy excised/non excised locus	34	24	1785 if non-excised; 188 if excised	gDNA from Rap/DMSO treated parasites
Positive control for diagnostic PCRs				
PfRON2 locus (positive control)	36	37	737	gDNA from transgenic parasites

Supplementary Table 3: Calculation of change in parasitaemia of mutant PfRH5 parasites over single cycle.

	PfRH5WT		PfRH5CL		PfRH5cKO	
**Assay 1**	**DMSO**	**Rap**	**DMSO**	**Rap**	**DMSO**	**Rap**
replicate 1 fold growth	6.34351071	6.57157389	7.51534752	5.80636304	9.38549495	0.26717461
replicate 2 fold growth	6.00381609	5.49126171	5.64993511	6.11689276	6.45046377	0.25975587
replicate 3 fold growth	5.39136319	5.36979135	6.66103704	7.56830652	6.82321213	0.32933334
average fold growth	5.91289666	5.81087565	6.60877322	6.49718744	**7.55305695**	0.28542127
**Relative parasitaemia**	78.284815	76.93409024	87.4979927	86.0206335	**100**	3.77888412
**Assay 2**						
replicate 1 fold growth	6.24770677	6.30998922	5.90156716	5.05312023	7.10700477	0.34238764
replicate 2 fold growth	5.62865207	5.7909814	5.74481518	5.15368275	6.66942217	0.39823733
replicate 3 fold growth	5.89238928	6.69766908	6.60099089	5.05872363	6.53879032	0.39849705
average fold growth	5.92291604	6.26621324	6.08245774	5.08850887	6.77173909	0.37970734
**Relative parasitaemia**	78.41746831	82.96261079	80.5297482	67.3701907	89.6556075	5.02720081
**Assay 3**						
replicate 1 fold growth	7.12599916	7.81737713	7.41549455	6.93862817	7.00005323	0.28170973
replicate 2 fold growth	7.10758512	6.7100046	7.41792987	6.52093775	5.81555905	0.23210405
replicate 3 fold growth	6.37655076	8.82631796	6.64740974	6.8460229	8.19053688	0.23746203
average fold growth	6.87004501	7.78456656	7.16027805	6.76852961	7.00204972	0.25042527
**Relative parasitaemia**	90.95714564	103.0651114	94.799736	89.613115	92.7048448	3.31554855

Supplementary Table 4: Summary data of comparison between relative parasitaemia of DMSO and Rap treated lines.

Line	Mean of DMSO	Mean of Rap	SEM of DMSO	SEM of Rap	Significant difference between DMSO and Rap (two-tailed, unpaired t-test)?	P-value	t statistic	df	CI
PfRH5cKO	94.12	4.041	3.068872338	0.51113859	Yes	0.000008	28.95	4	81.44 to 98.72
PfRH5WT	82.55	87.65	4.202175814	7.89966194	No	0.599128	0.5701	4	-29.92 to 19.74
PfRH5CL	87.61	81	4.119765643	6.89400957	No	0.456857	0.8228	4	-15.69 to 28.91

**Line**	PfRH5cKO	PfRH5WT	PfRH5CL
**Mean of DMSO**	94.12	82.55	87.61
**Mean of Rap**	4.041	87.65	81
**SEM of DMSO**	3.068872338	4.202175814	4.119765643
**SEM of Rap**	0.511138591	7.899661941	6.89400957
**Significant difference between DMSO and Rap (two-tailed, unpaired t-test)?**	Yes	No	No
**P-value**	0.000008	0.599128	0.456857
**t statistic**	28.95	0.5701	0.8228
**df**	4	4	4
**CI**	81.44 to 98.72	-29.92 to 19.74	-15.69 to 28.91

Note that statistical significance tests whether parasitaemia differs for each transgenic cell line ($PfRH5^{cKO}$, $PfRH5^{WT}$ or $PfRH5^{CL}$) independently, when treated with DMSO or Rapamycin. Therefore, no adjustments have been made for multiple comparisons.
