# Supplementary material for: The PfRCR complex bridges malaria parasite and erythrocyte during invasion
Source: Nature. 2023 Dec 20;625(7995):578–84. doi: 10.1038/s41586-023-06856-1 (PMC10794152; doi:10.1038/s41586-023-06856-1)
Supplement: Supplementary file 2 — Reporting Summary [file 41586_2023_6856_MOESM2_ESM.pdf]

nature portfolio

Corresponding author(s): Matthew K. Higgins

Last updated by author(s): 20-10-2023

Reporting Summary

Nature Portfolio wishes to improve the reproducibility of the work that we publish. This form provides structure for consistency and transparency in reporting. For further information on Nature Portfolio policies, see our Editorial Policies and the Editorial Policy Checklist.

Statistics

For all statistical analyses, confirm that the following items are present in the figure legend, table legend, main text, or Methods section.

n/a	Confirmed	
☐	☒	The exact sample size (*n*) for each experimental group/condition, given as a discrete number and unit of measurement
☐	☒	A statement on whether measurements were taken from distinct samples or whether the same sample was measured repeatedly
☐	☒	The statistical test(s) used AND whether they are one- or two-sided<br>*Only common tests should be described solely by name; describe more complex techniques in the Methods section.*
☒	☐	A description of all covariates tested
☒	☐	A description of any assumptions or corrections, such as tests of normality and adjustment for multiple comparisons
☐	☒	A full description of the statistical parameters including central tendency (e.g. means) or other basic estimates (e.g. regression coefficient) AND variation (e.g. standard deviation) or associated estimates of uncertainty (e.g. confidence intervals)
☐	☒	For null hypothesis testing, the test statistic (e.g. *F*, *t*, *r*) with confidence intervals, effect sizes, degrees of freedom and *P* value noted<br>*Give P values as exact values whenever suitable.*
☒	☐	For Bayesian analysis, information on the choice of priors and Markov chain Monte Carlo settings
☒	☐	For hierarchical and complex designs, identification of the appropriate level for tests and full reporting of outcomes
☒	☐	Estimates of effect sizes (e.g. Cohen's *d*, Pearson's *r*), indicating how they were calculated

Our web collection on statistics for biologists contains articles on many of the points above.

Software and code

Policy information about availability of computer code

Data collection	Software used for data collection are commercially available or openly accessible. Cryo-EM data were collected using EPU version 2.9.0.1519 (Thermo Fisher). Flow cytometry to measure parasite parasitaemia was collected using BD FACSDiva™ Software version 9, and for erythrocyte binding assays using ProSort™ Software (Bio-Rad). SPR data were collected using T200 Biacore Software version 2.0 (GE Healthcare). CD data were collected using Spectra Manager Version 2 (Jasco). Live cell imaging of parasite invasion for calcium flux assays was acquired using the NIS Advanced Research software package (Nikon). In-plate calcium flux assay data were acquired using CLARIOstar® software version v5.6.0 R2. Design and modeling of cysteine-locked versions of PfRH5 were performed using Rosetta version 2019.35.
Data analysis	Data analysis was performed as described in the methods section using commercially available or openly accessible software. Software used for cryo-EM data processing (SIMPLE 3.0, cryoSPARC v3.3.2, RELION 3.1.3, and DeepEMhancer v1.0.0) are standard and freely available to academic users. Model building and refinement was performed with COOT version ccp4-8.0, ISOLDE v1.5 and PHENIX v1.20.1-4487. A local installation of open source AlphaFold v2.1.1 was used for structure prediction. Chimera v1.16 and ChimeraX v1.4 and 1.5 were used for structure visualisation. GraphPad Prism version 8.4.3 and 9.2.0 was used to generate graphs and for statistical tests. T200 Biacore Evaluation software v1.0 is provided with the Biacore SPR machine and is standard in the field. Proteome Discoverer 2.5 and XlinkX node (Thermo Fisher) were used for cross-linking mass spectrometry analysis, and xiNET (https://https://crosslinkviewer.org/) was used for visualisation of crosslinks. Flow cytometry to measure parasite parasitaemia and erythrocyte binding was analysed using FlowJo version 10. Live cell imaging was processed using the NIS Advanced Research software package (Nikon).

For manuscripts utilizing custom algorithms or software that are central to the research but not yet described in published literature, software must be made available to editors and reviewers. We strongly encourage code deposition in a community repository (e.g. GitHub). See the Nature Portfolio guidelines for submitting code & software for further information.

March 2021

Data

Policy information about availability of data

All manuscripts must include a data availability statement. This statement should provide the following information, where applicable:

- Accession codes, unique identifiers, or web links for publicly available datasets
- A description of any restrictions on data availability
- For clinical datasets or third party data, please ensure that the statement adheres to our policy

Cryo-EM maps for PfRCR-Cy.003 are available from the Electron Microscopy Data Bank under accession codes EMDB-16569, EMDB-16637, EMDB-16638, EMDB-16639 and EMDB-16640, and its coordinates from the Protein Data Bank under accession code 8CDD. Cryo-EM maps for PfCyRPA-PfRIPR-Cy.003 are available under accession codes EMDB-16570, EMDB-16636 and EMDB-16635, and its coordinates from the Protein Data Bank under accession code 8CDE. In this study, previously published structures have been used for structural analysis; these can be found in the Protein Data Bank under accession codes 1IGT, 4HL0, 4U0Q, 4U0R, 4U1G, 5EZO, 5FTT, 5TIH, 5TIK, 6A69, 6RCU, 6RCV, 6Z2L, 7CKR, 7PHU, 7PHV, 7PHW, 7PI2 and 7UNY. Uncropped gels and source data for all graphs generated in this study are provided in Supplementary Figures and “source_data.xlsx” respectively. The "Rosetta Disulfidize" and "Rosetta FastRelax" scripts used to design cysteine-locked version of PfRH5 in this manuscript are also provided in "source_data.xlsx".

Human research participants

Policy information about studies involving human research participants and Sex and Gender in Research.

Reporting on sex and gender	N/A
Population characteristics	N/A
Recruitment	N/A
Ethics oversight	N/A

Note that full information on the approval of the study protocol must also be provided in the manuscript.

Field-specific reporting

Please select the one below that is the best fit for your research. If you are not sure, read the appropriate sections before making your selection.

☒ Life sciences ☐ Behavioural & social sciences ☐ Ecological, evolutionary & environmental sciences

For a reference copy of the document with all sections, see nature.com/documents/nr-reporting-summary-flat.pdf

Life sciences study design

All studies must disclose on these points even when the disclosure is negative.

Sample size	Sample sizes are described in figure legends and methods. No statistical method was used to predetermine sample size. Quantitative experiments were typically repeated in technical triplicate. Sample sizes for each experiment were chosen to be consistent with the field norms. For flow cytometry experiments, we collected at least three biological replicates, each with three technical replicates for each data point. FACS analyses to determine parasitaemia counts measured the numbers of parasite-infected cells in a total of 30,000 red blood cells. Since parasitaemia values ranged from 0.5-7.5%, this was considered sufficient to provide statistically robust measurements of parasitaemia. For imaging, at least 40 events resulting from 20-25 schizont egresses were analysed, as per field norms (e.g., https://doi.org/10.1038/s41467-023-40357-z) and to obtain data sufficient to observe statistical differences for control experiments.
Data exclusions	For both PfRH5 and PfRCR binding analyses by microscale thermophoresis, traces from concentrations greater than 2 µM for full-length basigin measurements and greater than 5 µM for basigin ectodomain measurements were excluded due to significant variations in raw fluorescence. As is standard for cryo-EM workflows, particles not contributing to classes of interest were excluded from the final reconstructions.
Replication	The number of repeats for each relevant experiment are given in figure legends and the methods. Typically, experiments were performed in independent technical triplicates. Flow cytometry data assaying erythroctye binding, detected via the Alexa-Fluor 488 anti-C-tag nanobody, was performed in technical duplicate. SPR analysis of PfCSS-PfPTRAMP binding to PfRIPR was performed four times. Plasmodium experiments were replicated both through technical replicates and 3 independent biological replicates. All attempts showed similar results.
Randomization	As is standard for cryo-EM workflows, particles were split randomly into two halves and refined separately, as is automatically implemented in cryoSPARC. Randomization of other experiments was not relevant to this study as no subjective judgements were required about which data to include, exclude, or measure.
Blinding	The investigators were not blinded to the group allocation during the experiment and/or when assessing the outcome, as analysis were

Blinding	performed on quantitative endpoints that are not subject to investigator bias.

Reporting for specific materials, systems and methods

We require information from authors about some types of materials, experimental systems and methods used in many studies. Here, indicate whether each material, system or method listed is relevant to your study. If you are not sure if a list item applies to your research, read the appropriate section before selecting a response.

Materials & experimental systems

n/a	Involved in the study
☐	☒ Antibodies
☐	☒ Eukaryotic cell lines
☒	☐ Palaeontology and archaeology
☒	☐ Animals and other organisms
☒	☐ Clinical data
☒	☐ Dual use research of concern

Methods

n/a	Involved in the study
☒	☐ ChIP-seq
☐	☒ Flow cytometry
☒	☐ MRI-based neuroimaging

Antibodies

Antibodies used	- Rabbit anti-Rh5 (polyclonal, primary antibody). This is described in PMID: 22186897 and 3872115 and was validated using immunofluorescence and Western blotting against lines with and without PfRH5, as well as by growth-inhibition assays.<br>- Rat anti-PfHSP70 (polyclonal, generated against recombinant P. falciparum HSP70 and described in 10.1126/sciadv.abe5396). Rat anti-PfHSP70 has been validated by western blot, using P. falciparum parasite lysates (10.1126/sciadv.abe5396).<br>-Goat anti-Rat HRP (Sigma, A9037; secondary antibody)<br>-Goat anti-rabbit HRP (BioRad, 172-1019; secondary antibody)<br>-R5.004 and R5.011 (anti-PfRH5 monoclonal antibodies, PMID 31204103). These were validated by immunofluorescence, Western bot, protein crystallography and growth-inhibition of Plasmodium falciparum in human erythrocytes, as described in PMID 31204103.<br>-Cy.003, Cy.004 and Cy.007 (anti-PfCyRPA monoclonal antibodies). These were validated in growth-inhibition assays and by protein crystallography in PMID 35177602.<br>-Goat anti-Human IgG (H+L) Cross-Adsorbed AlexaFluor™ 488 Secondary Antibody (Invitrogen, A11013, secondary antibody)<br>-CaptureSelect™ AlexaFluor™ 488 Anti-C-tag Conjugate (Thermo Scientific, 7213252100, nanobody)
Validation	Commercially available antibodies were validated by suppliers. Rabbit anti-RH5 has been characterized previously by IFA and immunoblot. Anti PfHSP70 rat antiserum was validated by immunoblot. R5.004, R5.011, Cy.003, Cy.004 and Cy.007 have been characterised by SPR and X-ray crystallography (PMIDs 31204103 and 35177602).

Eukaryotic cell lines

Policy information about cell lines and Sex and Gender in Research

Cell line source(s)	Commercial Freestyle™ 293-F cells (Thermo Fisher) and Expi293F™ cells (Thermo Fisher) were used for recombinant protein expression. Stable S2 cell lines for recombinant protein expression were generated in this study or reported previously (Hjerrild, K. A. et al. Production of full-length soluble Plasmodium falciparum RH5 protein vaccine using a Drosophila melanogaster Schneider 2 stable cell line system. Sci. Rep. 6, 30357 (2016); doi: 10.1038/srep30357). Native untransfected S2 cell lines are commercially available (ExpreS2ion Biotechnologies). Commercially available Sf9 insect cells (Thermo Fisher) were also used for recombinant protein expression. All Plasmodium cell lines are derived from P.falciparum 3D7, which is the first lab-adapted Plasmodium clone. The parasite clone originated from Dr David Walliker, Edinburgh University.
Authentication	Cell lines were not authenticated except for recombinant expression of the target protein. The first published Plasmodium falciparum genome (PMID: 12368864) is based on P. falciparum clone 3D7. We validated the 3D7 clone used by whole genome sequencing.
Mycoplasma contamination	All cell lines (Plasmodium and those used for recombinant expression) were not tested for mycoplasma contamination.
Commonly misidentified lines<br>(See ICLAC register)	3D7 is the routinely used Plasmodium falciparum lab strain for which the first whole genome sequence was available; it is not listed in ICLAC.

Flow Cytometry

Plots

Confirm that:

☒ The axis labels state the marker and fluorochrome used (e.g. CD4-FITC).

☒ The axis scales are clearly visible. Include numbers along axes only for bottom left plot of group (a 'group' is an analysis of identical markers).

☒ All plots are contour plots with outliers or pseudocolor plots.

☒ A numerical value for number of cells or percentage (with statistics) is provided.

Methodology

Sample preparation	For measurement of parasitaemia:<br>Parasitised and non-parasitised erythrocytes were fixed with 4% paraformaldehyde, 0.1% glutaraldehyde (Sigma) in PBS for 1 h at RT, followed by incubation for 1 h at 37°C with SYBR Green I (Life Technologies). Subsequently, 1µl of cells was resuspended in 2ml PBS prior to FACS readout.<br><br>For erythrocyte binding assays:<br>Red blood cells (O+ or O−) were washed twice in PBS containing 1% w/v bovine serum albumin (PBS/BSA), then 50 µl aliquots containing approximately 10 million cells were prepared. Following incubation with proteins for 1 hour at room temperature, cells were washed twice in PBS/BSA. To quantify the binding of PfRCR to erythrocytes, samples were stained in one of two ways as detailed below, then washed three times with PBS/BSA followed by dilution to approximately 6 million cells/ml before analysis. Staining procedure 1: to verify that the non-neutralising anti-PfRH5 antibody R5.011 could be used to quantify PfRCR binding to erythrocytes without impeding complex binding we first studied PfRCR labelled using the CaptureSelect™ AlexaFluor™ 488 Anti-C-tag Conjugate (7213252100, Thermo Scientific), incubated with cells for 1 hour at room temperature in the dark. Staining procedure 2: incubation with the monoclonal anti-PfRH5 antibody R5.011 as a primary antibody, followed by washing once, then incubation with a Goat anti-Human IgG (H+L) Cross-Adsorbed AlexaFluor™ 488 Secondary Antibody (A11013, Invitrogen). Primary and secondary antibodies were each used at 10 µg/ml in PBS/BSA for 1 hour at room temperature in the dark.
Instrument	BD LSR Fortessa X-20 Cell analyzer (for parasitaemia measurement) and Bio-Rad S3e Cell Sorter (for erythrocyte binding assays).
Software	To collect data, BD FACSDiva Software v9.0 (for parasitaemia measurement) and ProSort™ Software v1.6.0.12 (for erythrocyte binding assays) was used. Data were subsequently analysed using FlowJo v10.
Cell population abundance	For parasitaemia measurement:<br>Thirty thousand singlet events for each sample.<br><br>For erythrocyte binding assays:<br>Fifty thousand events were recorded for each sample.
Gating strategy	For parasitaemia measurement:<br>Gating for erythrocytes was achieved by plots of forward scatter area against side scatter area (gate = P1). Doublet discrimination required gating of forward scatter area against forward scatter width (gate = P2) followed by side scatter area against side scatter width (gate = P3). A SYBR Green-stained uninfected RBC sample was used as a negative control. Gating of SYBR Green positive, infected erythrocytes was achieved by plotting side scatter area against Alexa Fluor 488 area using 530/30 standard filter (gate = P4). Parasitaemia was determined by the number of cells identified in gate P4 as a percentage of those in gate P3.<br><br>For erythrocyte binding assays:<br>Erythrocytes were gated by plotting forward scatter area against side scatter area, then singlets identified by plotting forward scatter area against forward scatter height. Positively labelled erythrocytes corresponding to those with bound PfRCR were identified by plotting forward scatter area against Alexa-Fluor 488 area, with the positive gate placement guided by unstained red blood cells and those incubated with detection antibodies/nanobodies only. The number of positive cells is expressed as a percentage of the number of singlets recorded.

☒ Tick this box to confirm that a figure exemplifying the gating strategy is provided in the Supplementary Information.
