# Supplementary material for: The PfRCR complex bridges malaria parasite and erythrocyte during invasion
Source: Nature. 2023 Dec 20;625(7995):578–84. doi: 10.1038/s41586-023-06856-1 (PMC10794152; doi:10.1038/s41586-023-06856-1)
Supplement: Supplementary file 3 — Peer Review File [file 41586_2023_6856_MOESM3_ESM.pdf]

Peer Review File

Manuscript Title: The PfRCR complex bridges malaria parasite and erythrocyte during invasion

Reviewer Comments & Author Rebuttals

Reviewer Reports on the Initial Version:

Referees' comments:

Referee #1 (Remarks to the Author):

The PfCRCR is one of the important structures in merozoite invasion of red cells. It consists of five parasite molecules that bind to Basigin on the red cell. There have been many players in the story. To begin, Tom Wellems and Alan Cowman identified RH5 and showed that the molecule cannot be knocked out and is critical for invasion. Next comes Simon Draper who identified RH5 as a ideal molecule for a vaccine because it has few polymorphisms, unlike another candidate AMA1. A number of people were responsible for the discovery of how it binds merozoites as it has no transmembrane or GPI anchor. I will not try to give primacy, but one paper by Alan Cowman (Scally et al.) identified the five components that have a transmembrane merozoite attachment through PTRAMP. They also identified the five components for attachment and presented the structure for CSS. Cowman as well as Simon Draper are involved in identifying monoclonal antibodies to block invasion. It is an important story that will continue to evolve as in the present paper. Finally, the identification of Basigin as the red cell receptor of RH5 was by Gavine Wright using ALEXA, a tool for identifying low affinity binding.
There are two terms used in the paper: PfPCRCR that describes the five components of this complex – RH5, CyRPA, RIPR. CSS and PTRAMP and PfRCR that includes three components RH5. RIPPR and CyRPA called PRCR. The structure of these three are the focus of the paper and is defined at a high resolution. The structure of CSS and PTRAMP are not studied. In a paper by SW Scally et al. identified the structure of CSS as a six cys related to the domains on PfS48/45 and PfS230. The present paper does not mention the identification of the structure of CSS and should be included. In addition, the paper by Scally et al. gives many important characters of the structure.
The focus of the paper is on the details of the structure of PfRCR for which they have high resolution data to describe how these three components fit together. From this structure, they speculate on how antibodies will block binding of RH5 to basigin on the red cell. It also identifies which component is flexible, namely the C-terminal residues 717 to 1086 and as a consequence this region remains undefined.
A previous structure of CyRPA bound to RH5 was at low resolution 7.17 Angstrom (Wong et al.) and the present paper identifies no confirmational changes in RH5 induced by binding to CyRPA. The paper showed that the RH5 when it binds to Basigin does not change structure.
The tail of RIPR binds to CSS and PTRAMP that forms a connection between the red cell (RH5 and Basigin) and the merozoite (PTRAMP). A previous paper (Scally et al. Fig. 4, g) had shown high affinity binding of RH5/RIPR/CyRPA to red cells, presumably through its receptor Basigin on the red cell (Scally et al.
In thinking about this paper and the Scally et al. paper, the two papers tell the story of this important connection between the red cell and the merozoite, one with crystallography and one with a study of the components expressed and put together.

Referee #2 (Remarks to the Author):

The invasion of erythrocytes by Plasmodium falciparum merozoites requires specific receptor-ligand interactions. A key interaction, which is essential for invasion, involves the binding of the P. falciparum rhoptry protein PfRH5 with the RBC receptor Basigin. PfRH5 is part of a five-protein complex, referred to as the PfPCRCR complex, which includes PfRH5, PfCyRPA, PfRIPR, PfCSS and PfTRAMP. The precise mechanistic role of the PfRH5-Basigin interaction in the invasion process is not known. Some suggested roles include triggering a rise in calcium in the target RBC, insertion of PfRh5 in the RBC membrane following conformational changes induced by receptor-binding to form pores in the target cell and modulation of signaling pathways in RBCs causing cytoskeletal changes required for invasion. However, none of these hypotheses are supported with convincing experimental evidence. In this manuscript, the authors have used cryo-electron microscopy to determine the structure of the PfRH5-PfCyRPA-PfRIPR (PfRCR) complex bound to an inhibitory anti-PfCyRPA monoclonal antibody (mAb Cy.003) to 2.89 A^o resolution. This is a substantial improvement on the previous structure of the PfRCR complex at 7 A^o.

The structure of the PfRCR complex reported here confirms the previous report that PfCyRPA lies at the core of the complex and bridges PfRh5 and PfRIPR. This arrangement was also confirmed by chemical cross-linking experiments. The manuscript also reports that whereas the C-terminal region of PfRIPR in PfRCR is conformationally flexible, the interaction of PfRH5 with PfCyRPA in the complex is rigid with no conformational changes in either PfRH5 or PfCyRPA following assembly of the complex. The authors have also used a molecular genetic approach to replace wild type PfRh5 with a mutant in which the N and C-terminal regions of PfRH5 are locked in position by disulfide bonds. Deletion of PfRH5 is lethal for P. falciparum blood stages. However, gene replacement of the wild type PfRH5 gene with a mutant PfRH5 gene that expresses the rigid 'locked in' PfRH5 does not affect parasite growth. These observations confirm that PfRH5 is essential for blood stage growth but rule out the necessity for extensive conformational changes in PfRH5 following receptor-binding as previously proposed. Moreover, quantitative binding studies using microscale thermophoresis assays showed that PfRH5 and PfRCR bind the ectodomain of Basigin with similar binding affinity, which challenges the previously suggested hypothesis that assembly of PfRH5 in the PfRCR complex results in higher binding affinity for Basigin. The authors do not address the hypothesis that proposes that PfRH5 binding to Basigin leads to a rise in calcium in target RBCs resulting in cytoskeletal changes that are essential for invasion. The authors present a structure of the PfRCR complex at significantly higher resolution (2.89 A^o) than previously reported which enables a clearer view of the three parasite proteins in the PfRCR complex and how they interact with each other. The manuscript also presents structural analysis of the interaction mAbs against PfCyRPA and proposes hypotheses to explain their binding inhibitory activity.

The following specific points need to be addressed by the authors:
1. The structure of the PfRCR complex resolved to 2.9 A^o presented here is a great achievement and shows significantly greater detail compared to previously reported structures of the complex. The results confirm the overall organization of the components within the PfRCR complex as reported earlier. Does this higher resolution structure of the PfRCR complex provide any novel insights into the mechanistic role played by this complex in the process of RBC invasion? The authors should present such novel hypotheses regarding PfRCR function and validate them experimentally.

2. The observations based on structural, molecular-genetic and protein-protein interaction studies provide clear evidence against several hypotheses about the functional role of PfRH5 in RBC invasion that were proposed earlier. One key hypothesis is, however, not addressed. Does the interaction of PfRH5 trigger a rise in cytosolic calcium in RBCs? The authors should address this question experimentally using both recombinant PfRH5 as well as PfRCR complex and RBCs labeled with calcium sensitive fluorophores such as Fluo-4AM.

3. The manuscript also presents the structure of anti-PfCyRPA mAb Cy.003 bound to the PfRCR complex. Based on the structure of Cy.003 bound to the PfRCR complex, the authors propose that Cy.003 blocks PfRCR binding by steric hinderance. The authors should test this hypothesis. The

authors can test if Cy.003 blocks the binding of PfRCR with Basigin on RBCs (by FACS) and to recombinant Basigin (by SPR).

4. The authors state that better understanding of the structure of the PfRCR complex will inform development of novel immunogens based on its components for use as vaccines. The authors should provide an example of how such novel immunogens based on the improved understanding of the structure of PfRCR complex can be designed. Which antigen and what region of the antigen do the authors propose to target? How will such specific antibodies be generated by immunization?

Referee #4 (Remarks to the Author):

The manuscript by Farrell and colleagues describes the high resolution cryo-EM structure of the pfRCR complex which was previously shown essential for erythrocyte invasion by malaria-causing Plasmodium falciparum merozoites. The authors define the architecture of a multi-protein complex and the interaction between its components pfRH5, pfCyRPA and pfRIPR by cryo-EM. The authors updated the potential function of pfRH5 as a structural protein as opposed to the previous hypothesis of it being a channel. Furthermore, the authors suggested a model for the action of invasion-neutralizing antibodies based on a steric clashes.

The manuscript contains high quality biochemical and structural data which certainly advance the understanding of the arrangement of the complex. However, the manuscript somehow reveals limited insights into the function of the complex. If pfRCR is just a receptor and acts as a linker between the erythrocytes and merozoites then perhaps a full complex should be analyzed structurally and functionally (molecular simulations and/or biophysics).

Major comments:
1. The authors claim high resolution of their cryo-EM maps, in the range of 3 A with local resolution reaching 2.5 A. The analysis looks overall well done, however the density in the blue areas of higher local resolution in the Extended Data Fig.1e,f does not look like 2.5 A. This is perhaps because the maps are not locally sharpened? As these are the main structures of the manuscript, the authors should demonstrate locally sharpened maps and a gallery of EM density, similarly to what is done in the other figures.
2. I find the conclusion that PfRH5 does not open to get inserted into the erythrocyte membrane to form a pore based on the structures not entirely convincing. The authors solved the structure in several conditions and in complex with PfRCR and a Fab and got mostly ordered structures in all the cases and were even able to build/refine an atomic model into the density. This, however, does not mean that it cannot form a pore in different conditions or in presence of additional factors. The cross-linking experiments, on the other hand, are convincing. The section about pfRH5 should be re-written with the conclusion about the conformational change in the end of the section, not in the beginning
3. I do not understand the hypothesis for the mechanism of action of invasion-neutralizing antibodies. The authors suggest that the antibodies vs pfRH5 and pfCyRPA prevent the binding of pfRCR to basigin and the other potential receptors. But then figure 4C and ext. figure 8e,f show the complex of pfRCR and basigin. Figure 4c shows a minor clash of Cy.004 with the putative erythrocyte membrane, however I do not believe that it would be sterically prohibitable – a small rotation of the complex will leave enough room for the interactions to happen on the membrane.
4. The models presented in Figure 4 are poorly described, the generation of the models should be documented and the models should be deposited to a publicly available depository such as the pdb. Furthermore, some additional validation of the model should be performed such as the stability in a bilayer and / or ability to bind pfCSS with the potential interface.
Minor comments:
1. In my opinion there is no need to go into details why the previous structures could not achieve resolution higher than ~7 A. Structural analysis in the current manuscript obviously provides much

higher resolution and the maps also seem to slightly suffer from preferred orientation (by looking at the angular distribution and the validation reports).
2. I encourage the authors to consider if the second digit in reporting the resolution and the third digit in reporting RMSDs are meaningful.
3. From the figure 2B it is hard to appreciate an up to 11.5 A move of the blades 5 and 6. It would be useful to mark Val250 on the structure.
4. The interface between pfRIPR and pfCyRPA looks rather limited comparing to the i.e. pfCyRPA/pfRH5 interface, it would be interesting to characterize it in more detail.

Author Rebuttals to Initial Comments:

Referee 1 (blood stage malaria, mechanisms of cell invasion)

The PfCRCR is one of the important structures in merozoite invasion of red cells. It consists of five parasite molecules that bind to Basigin on the red cell. There have been many players in the story. To begin, Tom Wellems and Alan Cowman identified RH5 and showed that the molecule cannot be knocked out and is critical for invasion. Next comes Simon Draper who identified RH5 as a ideal molecule for a vaccine because it has few polymorphisms, unlike another candidate AMA1. A number of people were responsible for the discovery of how it binds merozoites as it has no transmembrane or GPI anchor. I will not try to give primacy, but one paper by Alan Cowman (Scally et al.) identified the five components that have a transmembrane merozoite attachment through PTRAMP. They also identified the five components for attachment and presented the structure for CSS. Cowman as well as Simon Draper are involved in identifying monoclonal antibodies to block invasion. It is an important story that will continue to evolve as in the present paper. Finally, the identification of Basigin as the red cell receptor of RH5 was by Gavine Wright using ALEXA, a tool for identifying low affinity binding.

There are two terms used in the paper: PfPCRCR that describes the five components of this complex – RH5, CyRPA, RIPR. CSS and PTRAMP and PfRCR that includes three components RH5. RIPPR and CyRPA called PRCR. The structure of these three are the focus of the paper and is defined at a high resolution. The structure of CSS and PTRAMP are not studied. In a paper by SW Scally et al. identified the structure of CSS as a six cys related to the domains on PfS48/45 and PfS230. The present paper does not mention the identification of the structure of CSS and should be included. In addition, the paper by Scally et al. gives many important characters of the structure.

The focus of the paper is on the details of the structure of PfRCR for which they have high resolution data to describe how these three components fit together. From this structure, they speculate on how antibodies will block binding of RH5 to basigin on the red cell. It also identifies which component is flexible, namely the C-terminal residues 717 to 1086 and as a consequence this region remains undefined.

A previous structure of CyRPA bound to RH5 was at low resolution 7.17 Angstrom (Wong et al.) and the present paper identifies no confirmational changes in RH5 induced by binding to CyRPA. The paper showed that the RH5 when it binds to Basigin does not change structure.

The tail of RIPR binds to CSS and PTRAMP that forms a connection between the red cell (RH5 and Basigin) and the merozoite (PTRAMP). A previous paper (Scally et al. Fig. 4, g) had shown high affinity binding of RH5/RIPR/CyRPA to red cells, presumably through its receptor Basigin on the red cell (Scally et al.

In thinking about this paper and the Scally et al. paper, the two papers tell the story of this important connection between the red cell and the merozoite, one with crystallography and one with a study of the components expressed and put together.

We thank the reviewer for their positive assessment of our work and its important place in the literature on this fascinating protein complex and leading vaccine candidate.

We have added an additional mention of the PfCSS structure in line 39, to complement the mention in line 1222 in the legend to Figure 4, thereby highlighting this achievement. As knowledge of the structure of PfCSS does not affect any of the conclusions of this study, and in view of the word limit, we do not feel that there is an appropriate place in the manuscript to discuss it in further detail.

Referee 2 (Plasmodium surface molecules, malaria vaccines)

The invasion of erythrocytes by *Plasmodium falciparum* merozoites requires specific receptor-ligand interactions. A key interaction, which is essential for invasion, involves the binding of the P. falciparum rhoptry protein PfRH5 with the RBC receptor Basigin. PfRH5 is part of a five-protein complex, referred to as the PfPCRCR complex, which includes PfRH5, PfCyRPA, PfRIPR, PfCSS and PfTRAMP. The precise mechanistic role of the PfRH5-Basigin interaction in the invasion process is not known. Some suggested roles include triggering a rise in calcium in the target RBC, insertion of PfRh5 in the RBC membrane following conformational changes induced by receptor-binding to form pores in the target cell and modulation of signaling pathways in RBCs causing cytoskeletal changes required for invasion. However, none of these hypotheses are supported with convincing experimental evidence. In this manuscript, the authors have used cryo-electron microscopy to determine the structure of the PfRH5-PfCyRPA-PfRIPR (PfRCR) complex bound to an inhibitory anti-PfCyRPA monoclonal antibody (mAb Cy.003) to 2.89Å resolution. This is a substantial improvement on the previous structure of the PfRCR complex at 7Å.

The structure of the PfRCR complex reported here confirms the previous report that PfCyRPA lies at the core of the complex and bridges PfRh5 and PfRIPR. This arrangement was also confirmed by chemical cross-linking experiments. The manuscript also reports that whereas the C-terminal region of PfRIPR in PfRCR is conformationally flexible, the interaction of PfRH5 with PfCyRPA in the complex is rigid with no conformational changes in either PfRH5 or PfCyRPA following assembly of the complex. The authors have also used a molecular genetic approach to replace wild type PfRh5 with a mutant in which the N and C-terminal regions of PfRH5 are locked in position by disulfide bonds. Deletion of PfRH5 is lethal for P. falciparum blood stages. However, gene replacement of the wild type PfRH5 gene with a mutant PfRH5 gene that expresses the rigid 'locked in' PfRH5 does not affect parasite growth. These observations confirm that PfRH5 is essential for blood

stage growth but rule out the necessity for extensive conformational changes in PfRH5 following receptor-binding as previously proposed. Moreover, quantitative binding studies using microscale thermophoresis assays showed that PfRH5 and PfRCR bind the ectodomain of Basigin with similar binding affinity, which challenges the previously suggested hypothesis that assembly of PfRH5 in the PfRCR complex results in higher binding affinity for Basigin. The authors do not address the hypothesis that proposes that PfRH5 binding to Basigin leads to a rise in calcium in target RBCs resulting in cytoskeletal changes that are essential for invasion. The authors present a structure of the PfRCR complex at significantly higher resolution (2.9Å) than previously reported which enables a clearer view of the three parasite proteins in the PfRCR complex and how they interact with each other. The manuscript also presents structural analysis of the interaction mAbs against PfCyRPA and proposes hypotheses to explain their binding inhibitory activity.

We thank the reviewer for their accurate and positive assessment of our work.

The following specific points need to be addressed by the authors:
1. The structure of the PfRCR complex resolved to 2.9 Å presented here is a great achievement and shows significantly greater detail compared to previously reported structures of the complex. The results confirm the overall organization of the components within the PfRCR complex as reported earlier. Does this higher resolution structure of the PfRCR complex provide any novel insights into the mechanistic role played by this complex in the process of RBC invasion? The authors should present such novel hypotheses regarding PfRCR function and validate them experimentally.

We thank the reviewer for their very positive assessment of our work. The reviewer asks whether the structure of PfRCR (and by implication, the novel structure of PfRIPR) provides any novel hypothesis about function within RBC invasion. Indeed, we thought deeply about this question as we analysed the structure and the domain composition of PfRIPR. The most exciting mechanistic outcome of our study is that PfRIPR is organised into a “core”, which binds to PfCyRPA/PfRH5 thereby linking it to basigin on the erythrocyte membrane, and a “tail” which links to the merozoite membrane through interaction with PfPTRAMP/PfCSS, thereby bridging erythrocyte and merozoite. This is coupled with our demonstration that previously proposed mechanisms, based around pore formation by PfRH5, are not correct. We have experimentally confirmed these discoveries in the manuscript, through cryoEM, biophysical analysis and a transgenic parasite experiment. These are novel and important mechanistic advances, presented for the first time here and will change the textbook on malaria invasion mechanisms.

PfRIPR is a complex molecule and there may well be more to the story than it acting solely as a bridge. For example, PfRIPR is essential in other *Plasmodium* species which lack PfRH5. Perhaps its complexity allows it to bind to different erythrocyte receptors in different species? Perhaps it allows lateral interactions with other components of the invasion machinery? There are a lot of options here, but these are not “follow-up” experiments which stem from the discoveries made here, but instead require new phases of discovery experiments. We would not be able to complete these in the time frame suitable for publication of the exciting data in this manuscript.

2. The observations based on structural, molecular-genetic and protein-protein interaction studies provide clear evidence against several hypotheses about the functional role of PfRH5 in RBC invasion that were proposed earlier. One key hypothesis is, however, not addressed. Does the interaction of PfRH5 trigger a rise in cytosolic calcium in RBCs? The authors should address this question experimentally using both recombinant PfRH5 as well as PfRCR complex and RBCs labeled with calcium sensitive fluorophores such as Fluo-4AM.

We thank the reviewer for mentioning the clear evidence that we provide against prevailing hypotheses currently in the literature. They are also correct in noting that we did not, in the first version of the manuscript, present data which addresses the issue of whether PfRH5 induces global changes in RBC calcium.

In the PfRH5 literature there are two separate and potentially overlapping claims. One is that PfRH5 directly forms a pore, while the second is that it causes increased intracellular calcium. Changes in calcium concentration may then be due to pore formation or due to a different mechanism, such as PfRH5-induced signalling pathways.

In our manuscript, we have focused on the claim that PfRH5 forms a pore, as this is the mechanism for which our structural data provides insight. We have now strengthened this section of the manuscript by adding a haemolysis assay, previously used (Wong et al, 2019) to indicate pore formation by PfRCR, and in which we observe no haemolysis due to PfRH5/PfRCR. This is shown in Extended Data Figure 4g and lines 167-170. Together with our cysteine-locking experiment, this indicates that PfRH5 does not need to form a pore during invasion.

We have now added additional data addressing the question of whether the formation of an RH5-mediate pore is required for the calcium flux which is observed at the merozoite-erythrocyte junction during invasion. This calcium flux is already known to be dependent on members of the PfPCRCR complex, as knockout lines abolish the calcium flux, showing that they act either at or before the point where the calcium flux occurs. We therefore measured whether the calcium flux occurs in our parasite line with a cysteine-locked PfRH5. When compared with the original strain, without the cysteine-lock, we find no change in the frequency of the calcium flux, with

more than 90% of invasion events showing a clear calcium flux. Therefore, the calcium flux, for which PfRH5 is essential, (i.e. it happens downstream of PfRH5 and may or may not directly involve PfRH5) does not require PfRH5-mediated pore formation. This data is now included in Figure 2h and lines 214-218 and allows us to make an additional mechanistic conclusion.

The reviewer refers in their suggestion to a specific experiment in previous studies (i.e. Aniweh et al, 2017, Cell Microbiol 16 e12747) in which RBCs are loaded with a calcium-sensitive fluorescent dye and the effect of adding PfRH5 to the cell surface is then assessed. PfRH5 induces increased calcium concentrations inside the RBC, which the authors speculate is related to the calcium flux at the merozoite-erythrocyte junction during invasion. The changes in calcium concentration in this experiment are very different in time scale and spatial organisation to the flux observed during an authentic invasion event. The entire process of parasite invasion takes ~25 seconds, with a very localised and transient calcium flux, while the calcium increase in this *in vitro* system grows continuously over minutes and occurs throughout the RBC. It is therefore not definitive that the calcium changes observed in this *in vitro* assay are related to those which occur during parasite invasion. Indeed, membrane fusion events also take place in an invasion process and mixing of compartments may lead to movements in calcium. We have attempted to repeat this experiment, and we see no significant changes in intracellular calcium concentrations with PfRH5, PfRH5ΔN or PfRCR over those observed in media (see below) but we prefer not to include this data in the manuscript as we would rather wait until a conclusive experiment is conducted in a genuine parasite invasion process before we come to a firm conclusion about the origin and importance of the calcium flux.

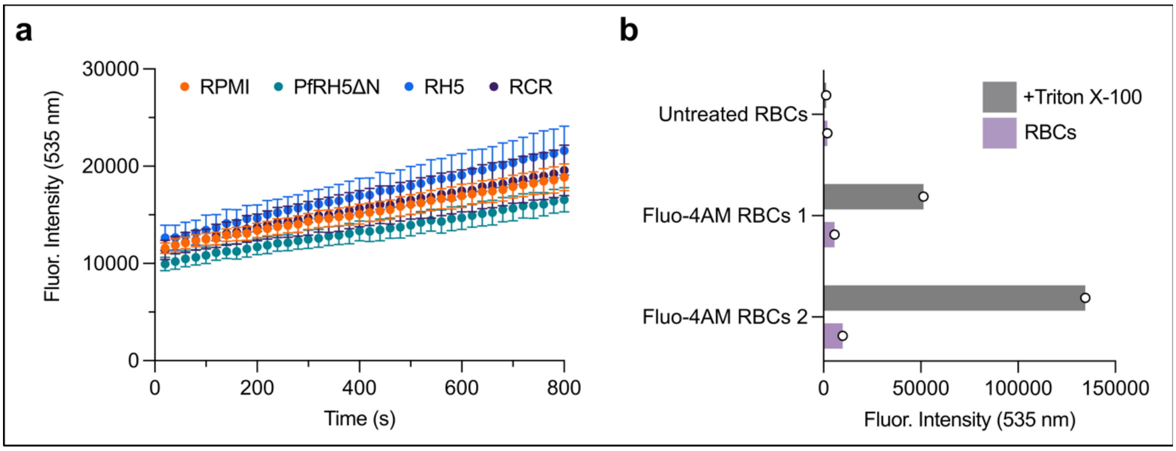

a, O+ RBCs were loaded with Fluo-4 through incubation with 6 μM Fluo-4AM in RPMI (supplemented with 0.5% w/v AlbuMAX™ and 4 g/L glucose) for 1 hour at 37 degrees, then washed and rested for 15 minutes. Protein complexes (PfRH5ΔN, PfRH5 or PfRCR) were added to Fluo-4 loaded RBCs at 2 μM in RPMI, then fluorescence (excitation 488 nm, emission 535 nm) measured over 800 seconds in black-bottomed 384 well plates. RPMI alone was used as a negative control. Measurements were recorded in triplicate. The mean and standard deviation are shown. **b**, To verify the loading of Fluo-4 in to RBCs, the lysis of RBCs was induced by addition of 0.1% v/v Triton X-100 in RPMI and fluorescence measured as in (a). Untreated RBCs were used as a control.

3. The manuscript also presents the structure of anti-PfCyRPA mAb Cy.003 bound to the PfRCR complex. Based on the structure of Cy.003 bound to the PfRCR complex, the authors propose that Cy.003 blocks PfRCR binding by steric hinderance. The authors should test this hypothesis. The authors can test if Cy.003 blocks the binding of PfRCR with Basigin on RBCs (by FACS) and to recombinant Basigin (by SPR).

We have followed the advice of the reviewer and have added a new data set to the manuscript which tests this hypothesis. The outcomes of these experiments are in agreement with our original proposal. Our modelling (Figure 4) does not suggest that Cy.003 will block PfRH5 from binding to basigin in an SPR experiment, but instead predicts a clash between the antibody and the membrane when PfRCR is bound to basigin in a membrane context. We have therefore used a FACS-based approach to test binding of soluble PfRCR to human erythrocytes. We have labelled PfRCR using the non-inhibitory antibody, R5.011, which we have shown does not affect erythrocyte binding (Extended Data Figure 8e). We then assessed the binding of labelled PfRCR to erythrocytes in the presence of four different antibodies - R5.004 which blocks binding of PfRH5 to basigin, and three antibodies which bind to CyRPA. We find that each of the CyRPA-binding antibodies prevents PfRCR from binding to erythrocytes, supporting the model in which they act through steric hinderance. Indeed, we find that their potency is related to the predicted likelihood that they will clash with the membrane, based how they project from PfRCR. These data are in full agreement with our hypothesis that the degree of steric clash of the antibodies with the membrane determines the growth-inhibitory activity of these antibodies. These findings are now presented in Figure 4d, Extended Data Figure 8e, Supplementary Figure 3 and described in lines 332-348.

4. The authors state that better understanding of the structure of the PfRCR complex will inform development of novel immunogens based on its components for use as vaccines. The authors should provide an example of how such novel immunogens based on the improved understanding of the structure of PfRCR complex can be designed. Which antigen and what region of the antigen do the authors propose to target? How will such specific antibodies be generated by immunization?

Currently the only known growth-neutralising PfRIPR-targeting mAbs target the PfRIPR tail. Our finding that the PfRIPR tail points towards the parasite membrane and interacts with parasite-associated PfCSS-PfPTRAMP therefore suggest that these antibodies function differently to those targeting PfRH5 and PfCyRPA, which we show act through a steric mechanism. Our studies open the way to understand how these antibodies work. However, the view that RIPR tail binding antibodies are most effective is based on a small number of antibodies and it is possible that the PfRIPR core will also contain neutralising epitopes. Our structural understanding of PfRIPR will now allow us to make individual domains, designed to fold out of the context of the rest of PfRIPR, which present epitopes which project in different directions from PfRCR (for example, towards the erythrocyte membrane, perpendicular to the membrane or towards the parasite). This will allow future studies to fully understand the antigenic cartography of PfRIPR. We have outlined this argument more clearly in lines 354-366.

Referee #4 (Remarks to the Author):

The manuscript by Farrell and colleagues describes the high resolution cryo-EM structure of the pfRCR complex which was previously shown essential for erythrocyte invasion by malaria-causing Plasmodium falciparum merozoites. The authors define the architecture of a multi-protein complex and the interaction between its components pfRH5, pfCyRPA and pfRIPR by cryo-EM. The authors updated the potential function of pfRH5 as a structural protein as opposed to the previous hypothesis of it being a channel. Furthermore, the authors suggested a model for the action of invasion-neutralizing antibodies based on a steric clashes.

The manuscript contains high quality biochemical and structural data which certainly advance the understanding of the arrangement of the complex. However, the manuscript somehow reveals limited insights into the function of the complex. If pfRCR is just a receptor and acts as a linker between the erythrocytes and merozoites then perhaps a full complex should be analyzed structurally and functionally (molecular simulations and/or biophysics).

We thank the reviewer for their positive comments about the quality of our data and the advances gained. In the latest version of the manuscript, we provide additional insight into the mechanisms of antibody-mediated inhibition, as well as additional data which shows that PfRH5 does not form a pore. Finally, we include the demonstration that the five component PfPCRCR complex forms with all five components coming together simultaneously (Lines 292-295 and Extended Figure 8b). Therefore, in addition to demonstrating the structure of PfRIPR and PfRCR for the first time, the manuscript shows that a prevailing hypothesis - that of PfRH5-mediated pore formation - is not supported by data. Instead, we show how the structure of PfRIPR, divided into a core and a tail, allows a different mode of action, bridging the parasite and erythrocyte membranes. This has substantial implications for both the mechanism of invasion and also for our understanding of how this is inhibited by antibodies. Our view is that this is a substantial functional and mechanistic advance.

Major comments:

1. The authors claim high resolution of their cryo-EM maps, in the range of 3 A with local resolution reaching 2.5 A. The analysis looks overall well done, however the density in the blue areas of higher local resolution in the Extended Data Fig.1e,f does not look like 2.5 A. This is perhaps because the maps are not locally sharpened? As these are the main structures of the manuscript, the authors should demonstrate locally sharpened maps and a gallery of EM density, similarly to what is done in the other figures.

We thank for the reviewer for their positive assessment of the quality of our structural analysis. We have made changes to the maps presented in the manuscript in response to their comments. We have clarified that the map shown in Figure 1 to illustrate resolution is an unsharpened consensus map. In Extended Data Figure 1 e/f we now show the final post-processed map, allowing the reader to better see the resolution. The galleries of density for regions of PfRH5 and PfRIPR are for the same map and are show in Extended Data Figures 4 and 7 and we clarify in the legend which map has been used in each case.

2. I find the conclusion that PfRH5 does not open to get inserted into the erythrocyte membrane to form a pore based on the structures not entirely convincing. The authors solved the structure in several conditions and in complex with PfRCR and a Fab and got mostly ordered structures in all the cases and were even able to build/refine an atomic model into the density. This, however, does not mean that it cannot form a pore in different conditions or in presence of additional factors. The cross-linking experiments, on the other hand, are convincing. The section about pfRH5 should be re-written with the conclusion about the conformational change in the end of the section, not in the beginning

We thank the reviewer for pointing out this lack of clarity in our argument. It was not our intention that the conclusion that PfRH5 doesn't insert into the membrane to form a pore derives from the structural comparison. The history here is that Wong et al (2019) suggested that the tip of PfRH5 is flexible in their 7Å resolution PfRCR structure, and this led them to the pore forming hypothesis. In reality, this is because they had not corrected for global flexibility of PfRCR in their analysis, meaning that the two distal tips of the elongated molecule appear disordered (i.e. the tip of a diving board moves further than the constrained end). When we corrected for this with our higher resolution data, we no longer see disorder at the tip of PfRH5. This is the argument which we were trying to get across. It is then the cysteine-locking experiment, which we agree with the reviewer is convincing, coupled with the new haemolysis assay and demonstration of a calcium flux in cysteine-locked parasites which make us confident that PfRH5 does not form a pore.

To make this clearer for reviewers, we have now divided this section of the manuscript into two separate sections and have clarified the text. In the first we present the evidence (structural and basigin-binding) that PfRH5 structure and function is not affected by assembly into the PfRCR complex. In the second we present data which shows that PfRH5 does not form a pore (haemoloysis assay and cys-locking experiment). We hope that this clarifies for readers from which experiment we are deriving each conclusion.

3. I do not understand the hypothesis for the mechanism of action of invasion-neutralizing antibodies. The authors suggest that the antibodies vs pfRH5 and pfCyRPA prevent the binding of pfRCR to basigin and the other potential receptors. But then figure 4C and ext. figure 8e,f show the complex of pfRCR and basigin. Figure 4c shows a minor clash of Cy.004 with the putative erythrocyte membrane, however I do not believe that it would be sterically prohibitable – a small rotation of the complex will leave enough room for the interactions to happen on the membrane.

Figures 4C and Extended Figures 8 E and F in the original version of the manuscript showed models containing just the Fab fragments of antibodies such as Cy.004 as the structurally characterised complexes were determined with such Fab fragments. *In vivo*, as well as in our new FACS experiment, the relevant form is intact monoclonal antibodies, including Fc fragments. The Fc and Fab fragments are flexibly linked and so it is not possible to present a rigid model for a full antibody in the figure, but we have now adapted the figure to show approximately where the Fc would be based on a full antibody crystal structure. The inclusion of the Fc increases the degree of clash with the membrane. Together with our new FACS data, which shows experimentally that this steric clash takes place and reduces PfRCR binding, we hope that this will convince the reviewer.

4. The models presented in Figure 4 are poorly described, the generation of the models should be documented and the models should be deposited to a publicly available depository such as the pdb. Furthermore, some additional validation of the model should be performed such as the stability in a bilayer and / or ability to bind pfCSS with the potential interface.

We have now included a section in the methods in which we describe the process which was used to produce these models, which is simply done through docking together experimentally-derived structures using their shared components. We also include the PDB codes for the models in supporting information, should authors wish to examine them, but as these involve combinations of the new structure presented here with other, already deposited structures, we do not think that it would be appropriate to deposit them in another form.

Minor comments:
1. In my opinion there is no need to go into details why the previous structures could not achieve resolution higher than ˜7 A. Structural analysis in the current manuscript obviously provides much higher resolution and the maps also seem to slightly suffer from preferred orientation (by looking at the angular distribution and the validation reports).

Our view is that it is important to mention that the limitation in resolution seen in the previous structure was due to preferred orientation as, without this knowledge, readers will not understand the strategy that we used to fix this problem. We have therefore left this information in manuscript. However, the reviewer is correct in that we did not completely abolish preferred orientation. We have therefore adapted line 65 to make this clear.

2. I encourage the authors to consider if the second digit in reporting the resolution and the third digit in reporting RMSDs are meaningful.

We have changed these as requested.

3. From the figure 2B it is hard to appreciate an up to 11.5 A move of the blades 5 and 6. It would be useful to mark Val250 on the structure.

We have made this change.

4. The interface between pfRIPR and pfCyRPA looks rather limited comparing to the i.e. pfCyRPA/pfRH5 interface, it would be interesting to characterize it in more detail.

We have added a little more information about the interface between PfCyRPA and PfRIPR in lines 251-252, to indicate the interface size and chemical nature, which complements the existing table (Extended Data Table 4) which presents the interactions which make up this interface. We also agree that the orientation of Figure 3C, chosen to show the continuation of the β-sheet from PfCyRPA to PfRIPR makes the interface appear smaller than it is. We have added an additional view of this interaction interface in Extended Data Figure 7c.

Reviewer Reports on the First Revision:

Referees' comments:

Referee #2 (Remarks to the Author):

The authors have submitted a revised manuscript and have addressed most of the issues raised by this reviewer. One important issue that the authors would prefer not to address in this paper (although they have performed the experiments) is the role of PfRCR in triggering a rise in Ca2+ in target RBCs during invasion. As previously demonstrated, the interaction of the PfRCR complex with its receptor Basigin on host RBCs is an essential interaction in the invasion process. A key event that follows the interaction of merozoites with RBCs is a rapid rise in Ca2+ in the target RBC. The rise in Ca2+ is essential for successful invasion. It has been proposed that the interaction of PfRCR with its RBC receptor Basigin is responsible for the rapid spike in Ca2+ in the target RBC. However, there is confusion in the field about the precise role of the PfRCR complex in triggering a Ca2+ spike in host RBCs during invasion. It has been previously suggested that PfRH5 may undergo a drastic conformational change following receptor engagement and insert into the RBC membrane to form pores, which could provide a channel for Ca2+ entry. The authors have addressed this possiblility here. Based on structural as well as molecular genetic approaches the authors rule out the possibility of conformational change and formation of pores in the RBC membrane by PfRH5. The authors produced a transgenic P. falciparum line that expresses a mutant PfRH5 with 5 engineered cysteine-cysteine pairs that result in a rigidly locked PfRH5 structure that is incapable of conformational changes. Replacement of wild type PfRH5 with the 'locked PfRH5' version did not affect either invasion efficiency or the ability to trigger a Ca2+ spike in target RBCs. This elegant experiment ruled out the 'pore forming' hypothesis for rise in host cell Ca2+. The authors have also directly tested if recombinant PfRH5 and PfRCR can trigger a rise in Ca2+ when incubated with RBCs. They did not find any evidence for any significant rise in Ca2+ in RBCs in these conditions. Given that PfRH5 as well as other components of PfRCR are functional (bind Basigin on RBCs) and correctly folded, the demonstration that PfRH5 and PfRCR do not trigger a rise in Ca2+ is an important result. It is not clear why the authors prefer not to include this data in the manuscript. It is important that the authors include this result in the manuscript and clearly state that PfRH5 and PfRCR do not directly trigger a rise in Ca2+ following Basigin binding. This result, although negative, would be an important contribution to the field. This observation is not inconsistent with the requirement of the PfRCR – Basigin interaction for a Ca2+ spike in RBCs demonstrated previously. For example, the PfRCR-Basigin interaction may be required for another interaction (eg. pore formation by alternative molecules or an alternative receptor binding event) that directly triggers a Ca2+ spike. It is important that the authors address this issue and include their new data on role of PfRCR in Ca2+ regulation in target RBCs. This would not only clarify an important question in the field but would open the door for discovery of novel interaction(s) more directly related to Ca2+ spike during host cell invasion by P. falciparum. The authors have addressed other questions adequately.

Referee #4 (Remarks to the Author):

Review R2

The manuscript has significantly improved by the incorporation of multiple improvements, clarifications and display items. Importantly, the flow cytometry approach contributed additional evidence supporting the suggested mechanism of action of pfRCR. At this point, while I believe that the core of the manuscript if correct, there are still significant technical concerns about handling structures and atomic models. While I believe that they will not affect the main conclusions of the manuscript, the standards of structure reporting require more work.

1. My take from looking at the maps and the validation reports is that the maps are produced correctly and the reported resolution is overall correct, although the maps are significantly anisotropic which should be mentioned in the text. However, the building of atomic models (which I could not successfully extract from the XLS attachment) seem to be done suboptimaly: the report 1292128303 has 89% residues within the density with an average Q-score of 0.38, while the report 1292128315 has 79% residues accommodated by the density with a Q-score of 0.35 (1-best 0 – poor). Some Q-scores are below zero (part 9.2 or the reports) and many areas of the maps have very low values. I encourage the authors look at the map-model fits or perhaps into local filtering of the maps, and re-build significant parts of the atomics models.

2. Looking at the validation reports for the deposited structures – the authors should deposit half-maps and masks in order to allow the reproducible calculation of resolution.

Minor:

1. Line 138: the authors write: "However, we hypothesised that this is an artifact of single particle averaging for a flexible molecular assembly" referring to the previous cryo-EM structure at subnanometer resolution (reference 20). And then they follow by the description that accounting for flexibility it was possible to obtain the structure at higher resolution. While I agree that the current structure is of higher quality, in order to experimentally test the hypothesis that the previous structure was produced with an artifact the authors would need to reprocess the corresponding dataset. While they are not doing it and there is no need to do so, I encourage the authors to reformulate this part just saying that they used newer algorithms and got a better structure.

2. line 141: the resolution of 4-5 A with some anisotropic resolution does not allow reliably building atomic maps into the maps. It is possible to fit the existing atomic models with some restrains and manual interventions (as done in the current manuscript). Please rewrite.

Author Rebuttals to First Revision:

Referees' comments:

Referee #2 (Remarks to the Author):

The authors have submitted a revised manuscript and have addressed most of the issues raised by this reviewer. One important issue that the authors would prefer not to address in this paper (although they have performed the experiments) is the role of PfRCR in triggering a rise in Ca2+ in target RBCs during invasion. As previously demonstrated, the interaction of the PfRCR complex with its receptor Basigin on host RBCs is an essential interaction in the invasion process. A key event that follows the interaction of merozoites with RBCs is a rapid rise in Ca2+ in the target RBC. The rise in Ca2+ is essential for successful invasion. It has been proposed that the interaction of PfRCR with its RBC receptor Basigin is responsible for the rapid spike in Ca2+ in the target RBC. However, there is confusion in the field about the precise role of the PfRCR complex in triggering a Ca2+ spike in host RBCs during invasion. It has been previously suggested that PfRH5 may undergo a drastic conformational change following receptor engagement and insert into the RBC membrane to form pores, which could provide a channel for Ca2+ entry. The authors have addressed this possiblility here. Based on structural as well as molecular genetic approaches the authors rule out the possibility of conformational change and formation of pores in the RBC membrane by PfRH5. The authors produced a transgenic P. falciparum line that expresses a mutant PfRH5 with 5 engineered cysteine-cysteine pairs that result in a rigidly locked PfRH5 structure that is incapable of conformational changes. Replacement of wild type PfRH5 with the 'locked PfRH5' version did not affect either invasion efficiency or the ability to trigger a Ca2+ spike in target RBCs. This elegant experiment ruled out the 'pore forming' hypothesis for rise in host cell Ca2+. The authors have also directly tested if recombinant PfRH5 and PfRCR can trigger a rise in Ca2+ when incubated with RBCs. They did not find any evidence for any significant rise in Ca2+ in RBCs in these conditions. Given that PfRH5 as well as other components of PfRCR are functional (bind Basigin on RBCs) and correctly folded, the demonstration that PfRH5 and PfRCR do not trigger a rise in Ca2+ is an important result. It is not clear why the authors prefer not to include this data in the manuscript. It is important that the authors include this result in the manuscript and clearly state that PfRH5 and PfRCR do not directly trigger a rise in Ca2+ following Basigin binding. This result, although negative, would be an important contribution to the field. This observation is not inconsistent with the requirement of the PfRCR – Basigin interaction for a Ca2+ spike in RBCs demonstrated previously. For example, the PfRCR-Basigin interaction may be required for another interaction (eg. pore formation by alternative molecules or an alternative receptor binding event) that directly triggers a Ca2+ spike. It is important that the authors address this issue and include their new data on role of PfRCR in Ca2+ regulation in target RBCs. This would not only clarify an important question in the field but would open the door for discovery of novel interaction(s) more directly related to Ca2+ spike during host cell invasion by P. falciparum. The authors have addressed other questions adequately.

We thank the referee for their supportive comments about our work.

Our decision not to include data in the manuscript for an assay in which we assessed changes in intracellular calcium concentrations of dye-loaded RBCs after incubating with PfRH5 and/or PfRCR was solely due to uncertainty about the relevance of this previously described assay to authentic erythrocyte invasion. In the assay, changes in calcium concentration caused by PfRH5 take place steadily over hundreds of seconds, while the calcium flux observed at the parasite-erythrocyte junction occurs for only seconds, during a ~25 second long invasion process. Rather than repeating assays from others, we therefore preferred to take our own approach, studying invasion processes using our cys-locked PfRH5. However, as the reviewer mentions, we did perform this previously described *in vitro* calcium flux assay and we did not observe significant changes to intracellular calcium concentration, despite using PfRH5 and PfRIPR which were functional in other assays. We had chosen to describe these results in the publicly available responses to referees.

However, at the referee's request we have now included this data in the manuscript in Extended Data Figure 4h-i, have mentioned our observations in the text in lines 172-174, and have added methods for this assay under the section 'Calcium flux assay' at lines 795-805.

Referee #4 (Remarks to the Author):

Review R2

The manuscript has significantly improved by the incorporation of multiple improvements, clarifications and display items. Importantly, the flow cytometry approach contributed additional evidence supporting the suggested mechanism of action of pfRCR. At this point, while I believe that the core of the manuscript if correct, there are still significant technical concerns about handling structures and atomic models. While I believe that they will not affect the main conclusions of the manuscript, the standards of structure reporting require more work.

We thank the referee for their supportive comment about the improvements to the manuscript. We are confident about the quality of our structure, which we have now double-checked. We have made some changes to our reporting, as described below.

1. My take from looking at the maps and the validation reports is that the maps are produced correctly and the reported resolution is overall correct, although the maps are significantly anisotropic which should be mentioned in the text.

We thank the reviewer for their positive comments about map quality. We have now carried out a quantitative assessment of the degree of anisotropy of the maps using 3DFSC analysis (Tan et al, 2017) and Fourier shell occupancy (FSO) analysis (Vilas et al, 2023). We have added the outcomes of these analyses in the image processing section of the methods at lines 511-515 and lines 533-534. The conclusion of this analysis is that, while there is some variation in resolution due to anisotropy in the maps, this is smaller than the variation in local resolution across the map. The level of anisotropy is not a cause for concern and has not had major effects on the interpretability of maps.

However, the building of atomic models (which I could not successfully extract from the XLS attachment) seem to be done suboptimaly: the report 1292128303 has 89% residues within the density with an average Q-score of 0.38, while the report 1292128315 has 79% residues accommodated by the density with a Q-score of 0.35 (1- best 0 – poor). Some Q-scores are below zero (part 9.2 or the reports) and many areas of the maps have very low values. I encourage the authors look at the map-model fits or perhaps into local filtering of the maps, and re-build significant parts of the atomics models.

For clarity, the only .xls file provided with the manuscript was the source data file. This does not contain the models associated with the new PfRCR-Cy.003 and PfCyRPA-PfRIPR-Cy.003 structures, but instead contains PDB files for the AlphaFold2 model of the PfRIPR tail and the composite models prepared for assemblies in Figure 4b-c and Extended Data Figure 8f-g. These can be converted into openable PDB files by copying the relevant column and pasting into a plain text file. We had shared the PDB files for the PfRCR-Cy.003 and PfCyRPA-PfRIPR-Cy.003 structures with the maps, as requested, but it seems as though these did not make it through in a form in which they could be identified?

We have checked the models for the PfRCR-Cy.003 and PfCyRPA-PfRIPR-Cy.003 structures and are confident that these are well built and are appropriate.

In our EMDB depositions, we provide both the unsharpened composite maps and the DeepEMhancer post-processed composite maps, previously using the latter for structure validation in the reports provided and as referred to here. We have since amended this, as while the post-processed map was used to aid with map interpretation for model building, structure refinement was performed against the unsharpened map, which we have now specified as the main map. These new validation reports indicate 91% atom inclusion and a Q-score of 0.43 for PfRCR-Cy.003, and 85% atom inclusion and a Q-score of 0.41 for PfRIPR-PfCyRPA-Cy.003.

To aid the referee, we present below an analysis of the Q-scores for PfRCR-Cy.003, calculated using the MapQ plug-in for Chimera (Pintilie et al, 2020), and have coloured the structural model according to Q-score. We find that regions of the model with lower Q-score correlate with regions of our EM map which have lower local resolution, consistent with the reported observation from the original publication that Q-score correlates with resolution. We are therefore confident that the Q-scores obtained for our models are appropriate and reflect an correctly built and refined structure.

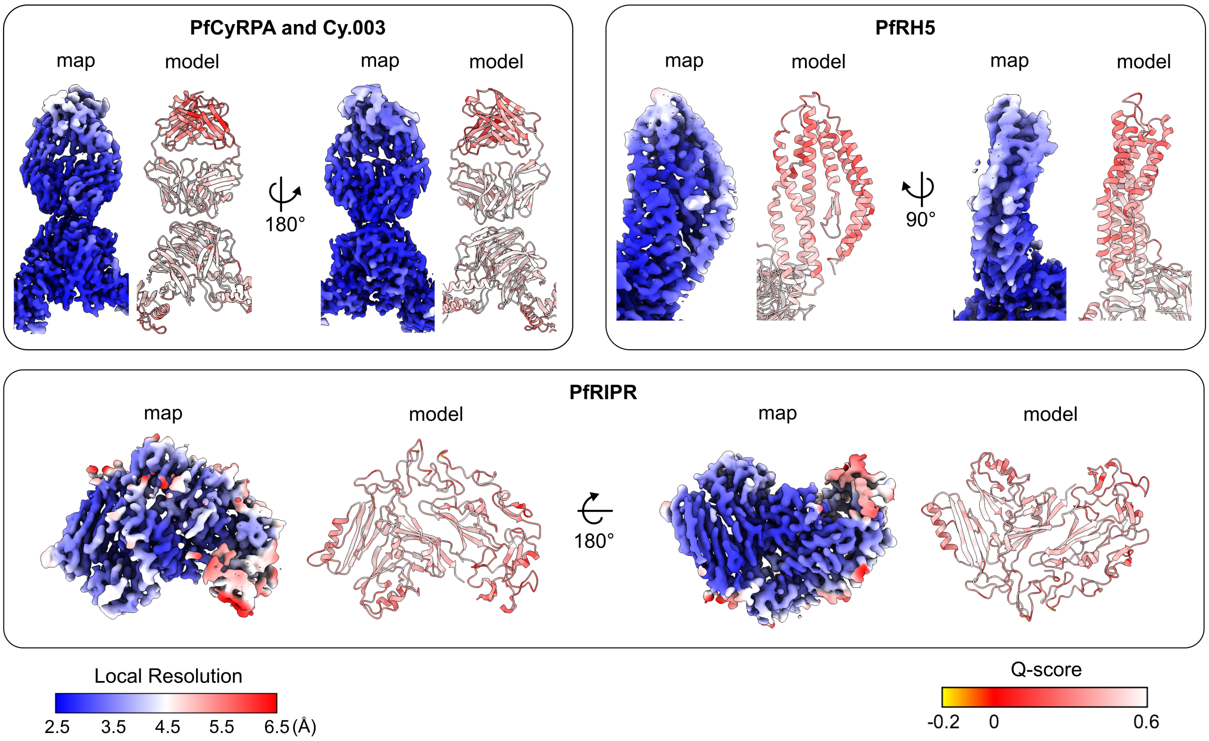

We also present, below, graphs showing the calculated Q-scores plotted for each residue in each chain, both for entire residues and for their backbones only. We have labelled the regions in each chain displaying lower Q-scores and draw thresholds indicating the expected Q-score for structures at different resolutions as calculated using the Chimera MapQ plugin (Pintilie et al, 2020). These show that for the Fab fragment of antibody Cy.003 (chains D and E), the constant domain of the Fab has a noticeably lower Q-score. This correlates with lower local resolution of this region of the map, and likely reflects flexibility between the variable and constant domains of the antibody. The structure of the constant domains of the antibody have not been used to derive new biological insights, but it makes more sense to include them as the density is of sufficient quality to place these. Some regions of PfRIPR (the N-terminus, EGF domains 1-2 and EGFs 3-4) and PfRH5 (regions at the tip of PfRH5 distant from the PCyRPA binding interface) also have lower Q-scores. These regions are of lower resolution in the map and are often surface exposed. We had previously described in the methods that some parts of the map corresponding to PfRIPR required AlphaFold models to be fully interpreted; we now clarify this further in lines 552-555, describing regions of PfRIPR which were modelled this way.

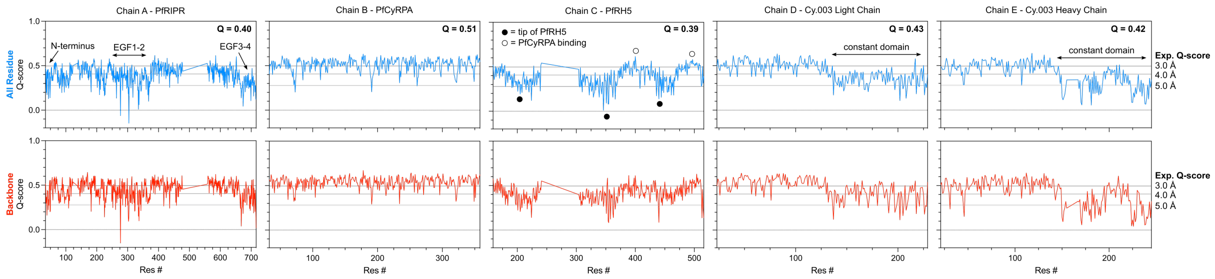

While the global resolution for the PfCyRPA-PfRIPR-Cy.003 map is only marginally lower than that for PfRCR-Cy.003, the region corresponding to PfRIPR has the same local resolution variation as that described above and is overall slightly poorer. We had described this in the methods and now provide a few additional details (lines 558-562). As described, PfRIPR in this case was primarily built by docking the model of PfRIPR from PfRCR-Cy.003 into the PfRIPR-PfCyRPA-Cy.003 map. The main biological insight from this overall model, and the reason we include it in the manuscript, is to show the conformational changes in PfCyRPA which occur on PfRH5 binding. The Q-score analysis for this model shows that PfCyRPA has an average Q-score of 0.47, giving confidence that our biological conclusions are valid.

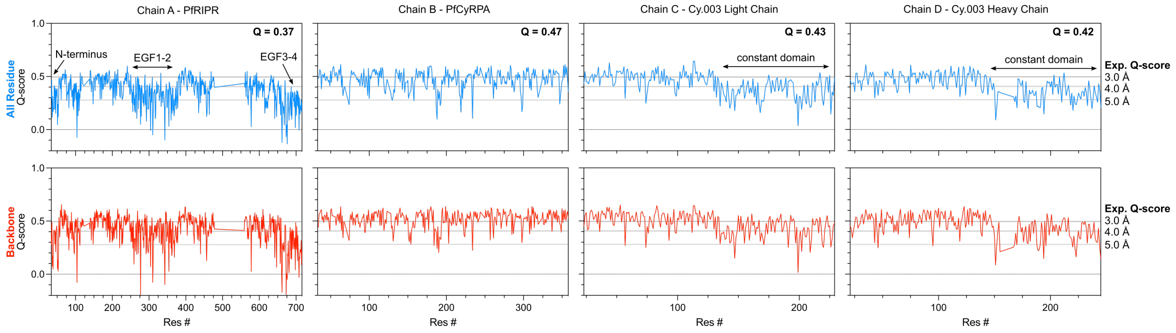

Therefore, while we acknowledge that the Q-scores are not uniform across our models, impacting the average Q-score reported during validation, we believe they reflect the variable resolution and interpretability of the cryo-EM maps and are consistent with a correctly built and refined structure. We are confident that our models are built and refined well, and that we have taken an appropriate approach to using them to derive biological insight.

2. Looking at the validation reports for the deposited structures – the authors should deposit half-maps and masks in order to allow the reproducible calculation of resolution.

We had already deposited the half-maps for consensus and local refinements in their EMDB entries, and have now also deposited the masks used for FSC to calculate resolution.

Minor:

1. Line 138: the authors write: “However, we hypothesised that this is an artifact of single particle averaging for a flexible molecular assembly” referring to the previous cryo-EM structure at subnanometer resolution (reference 20). And then they follow by the description that accounting for flexibility it was possible to obtain the structure at higher resolution. While I agree that the current structure is of higher quality, in order to experimentally test the hypothesis that the previous structure was produced with an artifact the authors would need to reprocess the corresponding dataset. While they are not doing it and there is no need to do so, I encourage the authors to reformulate this part just saying that they used newer algorithms and got a better structure.

We think that this comment has stemmed from a misunderstanding, as the map referred to in line 138 is our own PfRCR-Cy.003 consensus map which has its highest resolution at PfCyRPA at the centre of the PfRCR complex, and our observation of global rigid body motions along the length of PfRCR in 3D variability analysis (as shown in Supplementary Video 1). We did not intend to refer to the map from Wong et al here. As our map did improve through local refinement, this statement is correct. However, we see that this is possibly ambiguous in the original text and have therefore clarified this statement by citing Figure 1a (line 138) and Extended Data Fig 4c (line 143).

2. line 141: the resolution of 4-5 A with some anisotropic resolution does not allow reliably building atomic maps into the maps. It is possible to fit the existing atomic models with some restrains and manual interventions (as done in the current manuscript). Please rewrite.

The referee is correct and we have rephrased (lines 140-144).

Reviewer Reports on the Second Revision:

Referees' comments:

Referee #4 (Remarks to the Author):

The manuscript has again improved and my comments on the reporting of cryo-EM data have been addressed. From my side - I think that it can be published now.
